# Immunoinformatics based designing of a broad-spectrum multi-epitope vaccine against co-infection of *human metapneumovirus*,* respiratory syncytial virus*, and *influenza A virus*

**DOI:** 10.1038/s41598-026-40812-z

**Published:** 2026-02-23

**Authors:** Lu Li, Yong Chen, Shaoyong Wu, Chunyan Wu, Junhong Xie, Abdullah Shah, Xin Xie, Junyin Tan, Yudie Qin, Yuanlei Zeng, Amin Ullah Jan, Tianci Yang, Sadeeq Ullah

**Affiliations:** 1 Guangdong Provincial Key Laboratory of Medical Immunology and Molecular Diagnostics, The First Dongguan Affiliated Hospital, School of Medical Technology, Guangdong Medical University, Dongguan, 523808 China; 2 Department of Medical Laboratory, Affiliated Cancer Hospital of Chengdu Medical College, Chengdu Seventh People’s Hospital, Chengdu, 610231 China; 3 Department of Anesthesiology, State Key Laboratory of Oncology in South China, Guangdong Provincial Clinical Research Center for Cancer, Sun Yat- sen University Cancer Center, Guangzhou, 510000 China; 4 Department of Biochemistry, University of Veterinary and Animal sciences Swat (UVAS-Swat), Swat, 01923 Pakistan; 5 Department of Biotechnology, Shaheed Benazir Bhutto University Sheringal Dir Upper, Sheringal, 18300 Pakistan; 6 Institute of Infectious Disease, School of Medicine, Xiamen University, Xiamen, 361102 China

**Keywords:** Human metapneumovirus, Respiratory syncytial virus, Influenza A virus, Co-infection, Vaccine, Computational biology and bioinformatics, Immunology, Microbiology

## Abstract

Co-infections involving *human metapneumovirus* (*hMPV*), *respiratory syncytial virus* (*RSV*), and *influenza A virus* (*IAV*) often exacerbate disease severity in vulnerable populations. Here, we employed a structure-based immunoinformatics approach to design a multi-epitope subunit vaccine targeting these pathogens. The construct incorporated two epitopes each for cytotoxic T lymphocytes (CTLs), helper T lymphocytes (HTLs), and B cells, derived from the fusion proteins of *hMPV* and *RSV*, as well as the neuraminidase protein of *IAV*. These epitopes were linked with an adjuvant and optimized spacers to enhance immunogenicity and structural stability. Structural modeling confirmed correct folding, and molecular docking predicted a stable interaction with Toll-Like Receptor 4 (TLR4) − 277.43 kcal/mol. Molecular dynamics simulations indicated a compact and stable complex with restricted conformational motions, while MM/GBSA analysis yielded a favorable binding free energy (–121.72 kcal/mol) dominated by electrostatic and van der Waals interactions. Immune simulations predicted strong humoral and cellular responses, including high antibody titers, IFN-γ and IL-2 production, and durable memory formation. Codon optimization achieved a codon adaptation index (CAI) of 0.98 and a GC content of 51.24%, suggesting efficient expression in *Escherichia coli*. These findings highlight the construct as a structurally stable, immunogenic, and expression-ready vaccine candidate, warranting experimental validation against *hMPV*, *RSV*, and *IAV*.

**Supplementary Information:**

The online version contains supplementary material available at 10.1038/s41598-026-40812-z.

## Introduction

Co-infections are characterized by the simultaneous invasion and replication of multiple distinct pathogenic organisms within a single host, which can modulate host-pathogen interactions, alter immune responses, and complicate both the clinical and therapeutic management of infectious diseases^1–3^. Viral co-infections often involve complex interactions such as synergy, interference, mutual dependence, or competitive exclusion. Among these, interference between one virus suppresses the replication of another is commonly observed^4,5^.

Epidemiological studies report that co-infection with multiple respiratory viruses occurs in approximately 10–20% of clinical cases, highlighting its clinical significance^6,7^. However, the exact impact of viral co-infection on disease progression remains debated, as some studies report exacerbated severity or altered clinical course^5,8^, while others find no significant differences^6^.

Respiratory viral infections represent a major global health burden. Influenza alone causes nearly one billion cases yearly, leading to 3–5 million hospitalizations and an estimated 290,000–650,000 deaths, particularly among vulnerable populations such as children and the elderly^9^. Beyond mortality, these infections can also have a financial impact on patients, families, and the healthcare system^10^. Acute respiratory infections represent one of the leading causes of mortality among children worldwide^11,12^, and a large fraction of these infections is attributable to viral pathogens, especially *human metapneumovirus* (*hMPV*), *respiratory syncytial virus* (*RSV*), and influenza viruses^13^.

*Human respiratory syncytial virus* (*hRSV*) is an enveloped, negative-sense, single-stranded RNA virus belonging to the *Paramyxoviridae* family and the *Pneumovirus* genus. Its size ranges from 100 to 200 nm in diameter and exhibits either spherical or filamentous morphology^14^. Its single-stranded RNA genome is encapsidated and linked with a lipid bilayer that integrates the surface glycoproteins G (responsible for receptor binding) and F (facilitating membrane fusion). The virus makes several proteins from its genome. The hRSV genome encodes multiple proteins, including nonstructural proteins NS1, NS2, and M2, a phosphoprotein (P), and the RNA-dependent RNA polymerase (L), all important for viral replication and transcription^15^. *hRSV* is one of the leading viral pathogens globally, mostly responsible for acute lower respiratory tract infections in infants, young children, and the elderly^16^. It infects both the upper and lower parts of the airway. It can block airflow, cause bronchiolitis, and lead to apnea, pneumonia, or even respiratory failure^16^.

Similarly, *human metapneumovirus* (*hMPV*), genetically divided into two subgroups A and B, is a major cause of respiratory tract infections across age groups and often co-circulates with *RSV* and influenza. Co-infection involving *hMPV*, *RSV*, and *IAV* has been reported to elicit more complex and severe immune responses than single virus infections^17^. These viruses may interact competitively or synergistically, disrupting host immune regulation and resulting in delayed viral clearance, prolonged disease duration, and a heightened risk of complications, particularly in immunocompromised individuals^18^.

Both cellular and humoral immune responses are essential for antiviral defense. CD8^+^ cytotoxic T lymphocytes (CTLs) play a pivotal role in clearing infected cells, while CD4^+^ helper T lymphocytes (HTLs) assist in antibody production and coordinate adaptive immunity. CD4^+^ T cells also contribute to antiviral defense by secreting interferon-gamma (IFN-γ), a key cytokine in the antiviral response. These mechanisms highlight the importance of eliciting both arms of the immune system in vaccine development^19,20^.

Immunoinformatics, an interdisciplinary field combining immunology and computational biology, enables the prediction and modeling of immune responses using in silico tools. It offers a rapid and cost-effective alternative to conventional laboratory methods for identifying vaccine targets. Notably, this approach proved highly valuable during the COVID-19 pandemic, as it accelerated the development of subunit vaccines against SARS-CoV-2^21,22^.

In the context of viral co-infections, immunoinformatics enables the identification of conserved antigenic epitopes, prediction of CTL, HTL, and B-cell responses, and rational design of multi-epitope subunit vaccines (MESVs)^16,17^. Molecular-level simulations of host-pathogen interactions further enhance our understanding of immune complexity, supporting the development of targeted immunotherapies and multi-pathogen vaccine strategies^17^.

In this study, an immunoinformatics-based strategy was employed to design multi-epitope subunit vaccines (MESVs) targeting conserved regions of fusion and neuraminidase proteins from *RSV*, *hMPV*, and *IAV*. Neuraminidase (NA) plays a key role in the release of influenza virions by cleaving sialic acid residues, and immune responses directed against this protein can lessen viral shedding and disease impact while offering broader protection that is less influenced by antigenic drift^23^. Current research and health authorities highlight NA-based antigens as valuable candidates for next-generation “universal” influenza vaccines because of their capacity to induce cross-protective neuraminidase-inhibiting antibodies^24^. The fusion (F) protein of *hMPV* and *RSV* is a highly conserved antigen crucial for viral entry and a dominant target of neutralizing antibodies, making it ideal for vaccine development. Stabilizing the F protein in its prefusion conformation significantly enhances immunogenicity, enabling potent and cross-protective immune responses observed in both clinical and preclinical studies^25^. Computational tools were used to predict CTL, HTL, and B-cell epitopes, which were linked with appropriate adjuvants and spacers. Structural modeling, molecular dynamics simulations, and TLR-4 docking analyses validated the stability, immunogenic potential, and receptor-binding affinity of the designed construct. Compared to conventional vaccine approaches, this in silico strategy offers enhanced safety, precision, and cost-effectiveness for combating viral co-infections^26^.

## Methodology

**Fig. 1:**
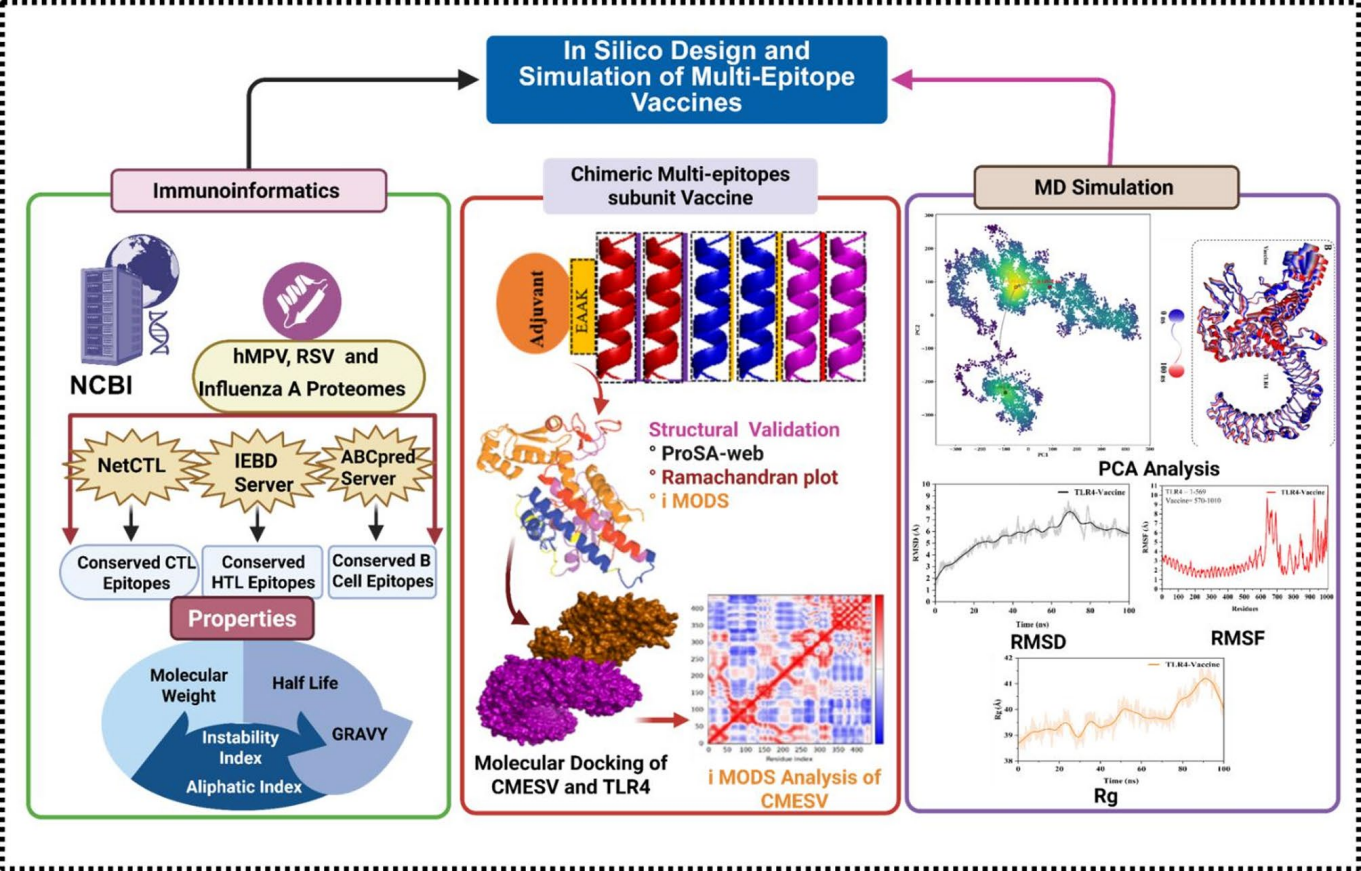
Overview of the computational-based steps used to design a chimeric multi-epitope vaccine against *hMPV*, *RSV*, and *IAV*.

Figure 1 illustrates the overall computational workflow employed to design and evaluate the chimeric multi-epitope vaccine construct against *hMPV*, *RSV*, and *IAV*. The process began with the retrieval of complete proteomes of the target viruses from the NCBI database, followed by immunoinformatics-based prediction of conserved CTL, HTL, and B-cell epitopes using NetCTL, IEDB, and ABCPred servers. The shortlisted epitopes were evaluated for key physicochemical properties such as molecular weight, half-life, instability index, aliphatic index, and GRAVY score to ensure stability and immunogenic potential. These epitopes were then assembled into a chimeric multi-epitope subunit vaccine (CMESV) construct, incorporating suitable linkers and an adjuvant sequence to enhance immune responsiveness. The tertiary structure of the vaccine model was built and validated through ProSA-web, Ramachandran plot analysis, and iMODS-based structural refinement. The final construct was subjected to molecular docking with TLR4 to assess receptor binding interactions, followed by molecular dynamics (MD) simulations for conformational stability, including PCA, RMSD, RMSF, and radius of gyration (Rg) analyses.

### Proteins sequences retrieval

The full-length proteomes of *Human Respiratory Syncytial Virus* (*RSV*), *Human Metapneumovirus* (*hMPV*), and *Influenza A Virus* (*IAV*), comprising nine, ten, and fourteen proteins, respectively were retrieved in FASTA format from the NCBI database (https://www.ncbi.nlm.nih.gov/) using the accession numbers of *RSV* (KF826817.1, KF826821.1 and KF530269.1), *hMPV* (KJ627434.1, KJ627437.1 and KJ627436.1), and *IAV* (CY146835.1 and EF190976.1) and saved for subsequent analysis to identify highly antigenic proteins for chimeric multiepitope subunit vaccine development. Individual protein accession numbers used in this study are listed in Supplementary Fig. S (1–3) and Table S (1–3).

### Prediction of antigenic and non-toxic proteins

The complete proteome of the *Human respiratory syncytial virus* (*RSV*), *Human Metaneumovirus* (*hMPV*), and *Influenza A virus* (*IAV*) (Nine, ten, and fourteen proteins respectively) were submitted to the VaxiJen v2.0 server (http://www.ddgpharmfac.net/vaxijen/VaxiJen/VaxiJen.html) to find out their antigenicity. The fusion (F) proteins of *RSV* and *hMPV* were selected because they are highly conserved, surface-exposed proteins essential for viral entry and major targets of neutralizing antibodies and T-cell responses. Similarly, the neuraminidase (NA) of *IAV* plays a vital role in viral release and cross-protective immunity. These proteins showed higher antigenicity scores (> 0.5) compared to other viral proteins, showing their better potential to induce strong and broad immune responses, thus making them ideal candidates for chimeric vaccine design. The ToxDL server (http://www.csbio.sjtu.edu.cn/bioinf/ToxDL/index.html) was utilized to evaluate the potential toxicity of the protein. This tool applies an interpretable deep learning based algorithm that classifies proteins into two categories, i.e., toxic **or** non-toxic, based on their sequence features.

### Identification of the conserved regions in antigenic proteins

The highly antigenic fusion protein of *RSV*, *hMPV*, and neuraminidase of *IAV* were fed to Clustal Omega (https://www.ebi.ac.uk/Tools/msa/clustalo/) online server for the identification of the conserved regions. The predicted epitopes for B and T cells were investigated in the aligned retrieved fusion protein of *RSV*, *hMPV*, and neuraminidase of *IAV* for conservation. The highly conserved region was chosen for the selection of each CTL, HTL, and BCL epitope.

### Prediction and screening of cytotoxic T-lymphocytes (CTL) epitopes

CTL epitopes help the immune system remove virus-infected cells and lower the viral load. To find these, we used the NetCTL 1.2 server (https://services.healthtech.dtu.dk/services/NetCTL-1.2/) to predict epitopes from the fusion proteins of *RSV* and *hMPV*, and the neuraminidase of *IAV*. The aforementioned tool checks MHC class I binding, proteasome cleavage sites, and TAP transport. We selected epitopes with a score above 0.75.

### Prediction of HTL epitopes

We used the Immune Epitope Database (IEDB) (http://tools.iedb.org/mhcii/) to find helper T cell (HTL) epitopes from the fusion proteins of *RSV* and *hMPV*, and the neuraminidase of *IAV*. Epitopes with the lowest percentile ranks showing strong MHC class II binding were chosen for the vaccine design.

### Prediction of B-cell epitopes

B-cell epitopes were predicted using the ABCPred server (http://crdd.osdd.net/raghava/abcpred/), which utilizes a recurrent neural network-based algorithm to identify sequences likely to elicit antibody responses^27^. Epitopes were selected based on high prediction scores, with a threshold value of 0.80 applied to ensure the inclusion of highly probable antigenic regions.

### Multiple epitope vaccine designing and evaluation

The selected epitopes comprising cytotoxic T lymphocytes (CTLs), helper T lymphocytes (HTLs), and B-cell epitopes derived from the fusion proteins of *RSV* and *hMPV*, as well as the neuraminidase of *IAV*, were assembled into a chimeric multi-epitope subunit vaccine construct. To ensure proper immunogenic presentation and structural flexibility, AAY, GPGPG, and KK linkers were employed to join CTL, HTL, and B-cell epitopes, respectively. Moreover, the human-derived β-defensin-2 was selected as an adjuvant for its potent antimicrobial and immunomodulatory properties, acting as a TLR4 agonist that promotes dendritic cell maturation and cytokine secretion crucial for adaptive immune activation. Its incorporation enhances antigen presentation and stimulates robust humoral and cellular immune responses. To further optimize immune stimulation and structural stability, β-defensin-2 was conjugated to the N-terminus of the vaccine construct via an EAAK linker, ensuring spatial separation and efficient epitope exposure. This strategic design strengthens the overall immunogenic efficacy of the multi-epitope vaccine against viral co-infections^28^.

### Antigenicity and allergenicity of construct

We used the VaxiJen v2.0 server (https://www.ddg-pharmfac.net/vaxijen/VaxiJen/VaxiJen.html) to test if the vaccine is likely to activate an immune response. A score above 0.4 was considered antigenic. We also checked allergenicity using AllerTOP v2.0 (https://www.ddg-pharmfac.net/AllerTOP/) to make sure the vaccine was safe and not likely to cause allergies.

### Physicochemical properties of the construct

We used the ProtParam tool (https://web.expasy.org/protparam/) on the ExPASy server to analyze the vaccine’s properties, including amino acid content, molecular weight, pI, stability, half-life, aliphatic index, and GRAVY score.

### 3-D structure prediction and validation

The accurate 3D structure of the proteins is crucial for vaccine development, as it enables precise epitope mapping, stable construct modeling, and effective prediction of antigen-receptor interactions essential for immune activation. Thus, we used the Robetta server (https://robetta.bakerlab.org/) to model the 3D structure of the vaccine using its amino acid sequence in FASTA format. The chosen model was checked with ProSA-Web (https://prosa.services.came.sbg.ac.at/prosa.php) and PROCHECK (https://saves.mbi.ucla.edu/).

### Population coverage analysis

The population coverage of the selected epitopes was evaluated using the IEDB Population Coverage tool (https://tools.iedb.org/population/). Predicted HLA-binding epitopes were assessed to estimate their potential recognition among global and regional human populations. The analysis incorporated the frequency distribution of HLA class I and class II alleles across diverse ethnic groups. Overall and overlapping population coverages were computed to determine the vaccine construct’s ability to induce broad immune responses worldwide.

### Molecular docking with TLR4

Effective stimulation of the human immune response requires the vaccine to interact with host immune receptors. Molecular docking serves as a crucial in silico approach to predict the binding affinity and three-dimensional (3-D) orientation between a ligand and its target receptor. For this purpose, the 3-D structure of Toll-like receptor 4 (TLR-4; PDB ID: 2Z63) was retrieved from the RCSB Protein Data Bank (https://www.rcsb.org/). Before docking, all heteroatoms and water molecules were removed using PyMOL to prepare the receptor structure. The refined TLR-4 structure and the modeled vaccine concept were submitted to the HDOCK server (http://hdock.phys.hust.edu.cn/) to evaluate their binding interactions. The top-ranked docked complex, determined based on docking score, was selected for further structural and interaction analysis by using online server (https://bio.tools/pdbsum_generate).

### Codon optimization and vaccine in silico cloning

Codon optimization is a computer-based method used to improve protein expression by adjusting codons to match the host organism. We used the JCat tool (http://www.jcat.de/) to convert the vaccine’s amino acid sequence into a DNA sequence optimized for *E. coli K12*. JCat also gave CAI and GC content values to check if the gene would express well. NdeI and XhoI sites were added to the ends of the sequence for cloning into the pET-28a(+) vector. The cloning setup was checked using SnapGene.

### Immune simulation

The C-immSim online server (https://kraken.iac.rm.cnr.it/C-IMMSIM/) was used for immune simulation to assess the human immune response to the constructed vaccine. The aforementioned server evaluated the antigenicity of the developed vaccine and its capacity to elicit immunological responses upon administration. The server evaluated the quantity of immune cells like helper T-cells 1 and 2 (Th1 and Th2), respectively. Additionally, the server measured various other immunological responses, including the production of antibodies, cytokines, and interferon, in response to administered vaccines.

### Molecular dynamics simulation of the constructed vaccine and human TLR4 complex

Molecular dynamics (MD) simulations were performed using AMBER v24^29^ to assess the structural stability and conformational dynamics of the chimeric multi-epitope vaccine in complex with human Toll-Like Receptor 4 (TLR4; PDB ID: 2Z63). The initial complex was prepared in PyMOL by removing crystallographic water molecules and heteroatoms. The ff19SB force field was used for protein parametrization^30^. The system was solvated in an octahedral box of OPC water molecules with a 12.0 Å buffer and neutralized with Na^+^ and Cl^−^ ions. NaCl was added to a final concentration of 0.15 M to mimic physiological conditions^31^. Energy minimization was carried out in two stages: 10,000 steps with positional restraints (10 kcal/mol·Å²) on protein heavy atoms, followed by 50,000 steps without restraints. The system was gradually heated from 0 K to 300 K over 500 ps under constant volume (NVT), using a Langevin thermostat (collision frequency of 2 ps^− 1^). This was followed by 1 ns of equilibration at 1 atm under constant pressure (NPT), using the Berendsen barostat, during which positional restraints were progressively removed. A 100 ns production run was performed at 300 K and 1 atm using periodic boundary conditions. Long-range electrostatics were handled with the particle mesh Ewald method. SHAKE constraints were applied to hydrogen-containing bonds, allowing a 2 fs timestep. Snapshots were saved every 10 ps for analysis. Analysis was performed using CPPTRAJ^32^. Root mean square deviation (RMSD) was calculated to assess the global stability of the complex over time. Root mean square fluctuation (RMSF) was calculated to examine residue-level flexibility. The radius of gyration (Rg) was calculated to monitor overall structural compactness. Intramolecular hydrogen bonds were analyzed to evaluate the complex stability. Principal component analysis (PCA) was conducted to identify dominant motions during the simulation. The binding free energy between the vaccine and TLR4 was estimated using the MM/GBSA method from extracted snapshots of the production phase. VMD and PyMOL were used for structural visualization.

## Results

Vaccines provoke the immune system and play a crucial role in fighting against different diseases. Small antigenic determinants can efficiently set off immune reactions rather than entire virus proteins^33^. By determining possible immunogenic sites, immunoinformatics provides a fast and accurate method of vaccine design^34^. The fusion protein of RSV and hMPV and neuraminidase of IAV strains were analyzed using an immunoinformatics method in order to find immunogenic, non-allergenic CTL, HTL, and B-cell epitopes. Broad protection against RSV, hMPV, and IAV is sought by designing chimeric multi-epitope subunit vaccines from these epitopes^35^.

### Identification of antigenic proteins and conserved regions in RSV, hMPV, and IAV for chimeric multiepitope vaccine

Among the seven proteins of *RSV* and *hMPV* and fourteen proteins of *IAV* analyzed for antigenicity, the fusion proteins of *RSV* and *hMPV*, along with the neuraminidase protein of *IAV*, showed the highest antigenicity scores (0.5219 for *RSV*, 0.4775 for *hMPV*, and 0.5024 for *IAV*). Thus, these proteins were selected for the construction of the chimeric multi-epitope subunit vaccine (Supplementary Table S4). The fusion and neuraminidase proteins of the selected viruses presented highly conserved regions across multiple isolates and strains (Supplementary Figs. S1–S3). These conserved regions served as the basis for identifying HTL, CTL, and BCL epitopes incorporated into the final vaccine construct. Targeting such conserved viral regions reduces the likelihood of immune escape and enhances vaccine efficacy against diverse viral strains.

### Cytotoxic T cell epitope prediction and screening

CTL epitopes play a critical role in recognizing virus-infected cells and eliciting cellular immune responses. From the fusion and neuraminidase proteins of *hMPV*, *RSV*, and *IAV*, a total of 531, 566, and 461 CTL epitopes were predicted, respectively. The top ten epitopes with the highest prediction scores were shortlisted from each protein (Supplementary Tables S5–S7). Among these, two highly antigenic, non-allergenic, non-toxic, and conserved epitopes from each viral protein were selected for incorporation into the final chimeric multi-epitope vaccine construct (Table 1).

**Table 1:** Selected CTL epitopes of *hMPV*, *RSV*, and *IAV* used in the proposed chimeric multi-epitope subunit vaccine.

Virus	Protein	Peptide sequence	Peptide position	Conserved region	Combined score	Antigenicity score	Toxicity	Allergenicity
*hMPV*	Fusion	LSVLRTGWY	35–44	Yes	1.6130	0.8622	NO	Non-allergenic
		FSDNAGITP	88–97	Yes	0.8963	0.7563	NO	Non-allergenic
*RSV*	Fusion	NIDIFNPKY	382–391	Yes	2.5413	0.7826	NO	Non-allergenic
		LIAVGLLLY	540–549	Yes	2.3421	0.8288	NO	Non-allergenic
*IAV*	Neuraminidase	WTSASSISF	421–430	Yes	1.5752	1.0334	NO	Non-allergenic
		ELNAPNSHY	252–261	Yes	1.1526	0.1266	NO	Non-allergenic

### Helper T lymphocytes (HTL) epitopes prediction

HTLs play a vital role in the coordination of immune responses by the secretion of cytokines and the activation of other immune cells. Accordingly, a total of ten top-ranked HTL epitopes were selected from the fusion proteins of *hMPV*, *RSV*, and the neuraminidase protein of *IAV* (Supplementary Table S3). Among these, two epitopes from each virus exhibited high antigenicity, non-allergenicity, and non-toxicity and were selected for inclusion in the final chimeric multi-epitope vaccine construct (Table 2).

**Table 2:** Selected HTL epitopes of *hMPV*, *RSV*, and *IAV* used in the proposed chimeric multi-epitope subunit vaccine.

Virus	Protein	Allele	Peptide position	Peptide sequence	*P*. Rank	Antigenicity score	Toxicity	Allergenicity
*hMPV*	Fusion	HLA-DRB1*07:01	229–243	RAVSYMPTSAGQIKL	0.06	0.8830	NO	Non-allergenic
		HLA-DRB1*15:01	119–133	TAGIAIAKTIRLESE	0.12	0.8439	NO	Non-allergenic
*RSV*	Fusion	HLA-DRB3*02:02	234–248	TREFSVNAGVTTPVS	0.04	0.2788	NO	Non-allergenic
		HLA-DRB1*03:01	263–277	DMPITNDQKKLMSN	0.04	0.2076	NO	Non-allergenic
*IAV*	Neuraminidase	HLA-DRB3*01:01	214–228	SNRPWVSFDQNLDYQ	1.30	0.6043	NO	Non-allergenic
		HLA-DRB5*01:01	334–348	RPCFWVELIRGRPKE	1.30	0.9819	NO	Non-allergenic

### B-Cell epitopes prediction

B lymphocytes play a crucial role in adaptive immunity due to the production of antibodies that neutralize pathogens, thus offering long-term protection. A total of 21, 23, and 17 B-cell epitopes were identified from the fusion proteins of *hMPV* and *RSV* and the neuraminidase protein of *IAV*, respectively (Supplementary Table S4). Ten top-scoring epitopes from each protein were shortlisted, and two highly antigenic, non-allergenic, non-toxic, and conserved epitopes from each virus were selected for inclusion in the final chimeric multi-epitope vaccine construct (Table 3).

**Table 3:** BCL epitopes of *hMPV*, *RSV*, and *IAV* used in the proposed chimeric multi-epitope subunit vaccine.

Virus	Protein	Epitope	Start position	Score	Antigenicity score	Toxicity	Conserved region
*hMPV*	Fusion	QHVIKGRPVSNSFDPI	434	0.88	0.9931	NO	Non-allergenic
		CWIIKAAPSCSEKDGN	283	0.92	0.8104	NO	Non-allergenic
*RSV*	Fusion	NIDIFNPKYDCKIMTS	383	0.89	0.7806	NO	Non-allergenic
		SCSISNIETVIEFQQK	211	0.86	1.0332	NO	Non-allergenic
*IAV*	Neuraminidase	IGYICSGVFGDNPRPK	299	0.91	0.5897	NO	Non-allergenic
		EECSCYPDTGKVMCVC	261	0.91	0.4128	NO	Non-allergenic

### Chimeric multi-epitope vaccine construction

**Fig. 2:**
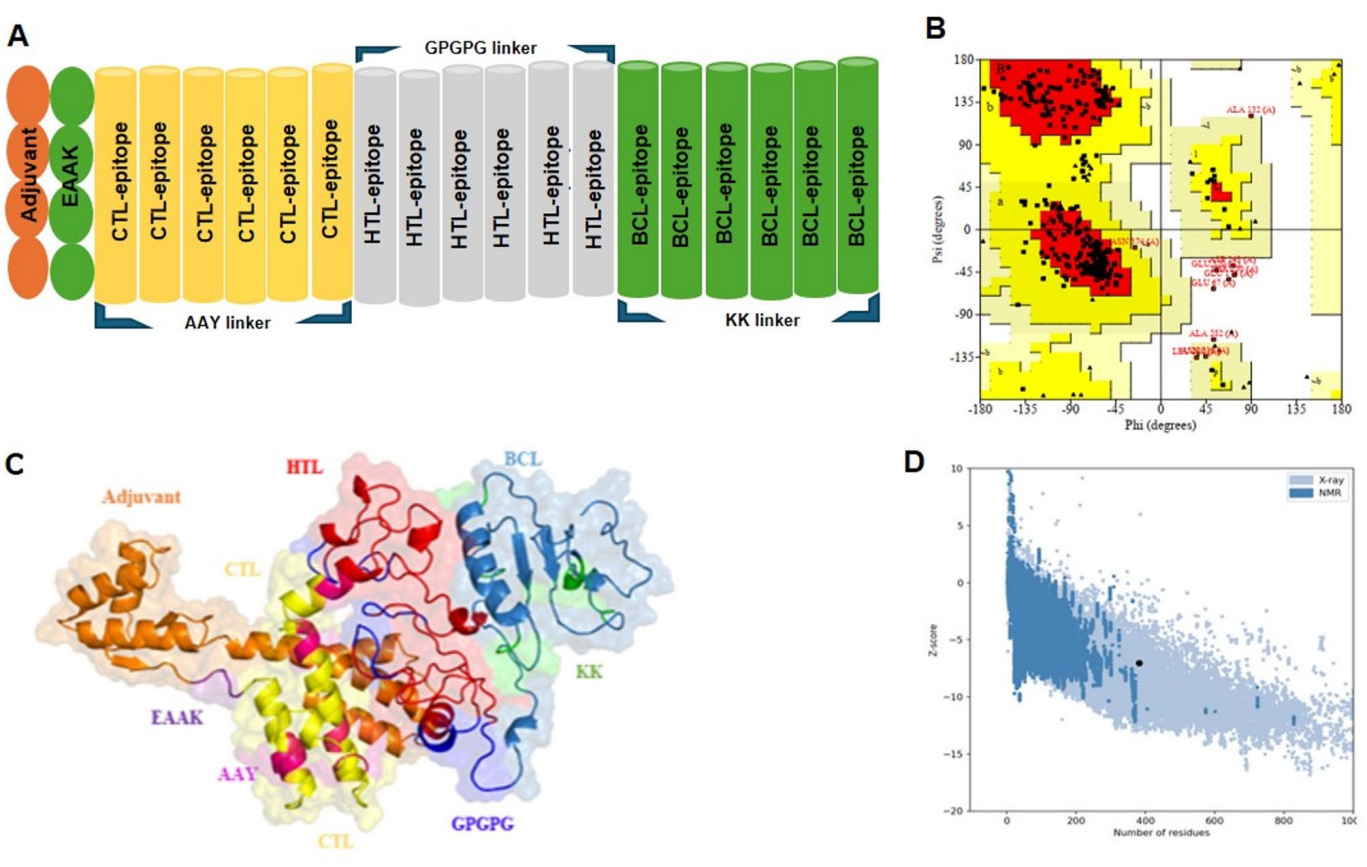
3-D structure modeling and validation of the chimeric multi-epitope vaccine. (**A**) Chosen epitopes and linkers used in the chimeric subunit vaccine design, (**B**) Ramachandran plot analysis showing the distribution of backbone dihedral angles, indicating model stereochemical quality. (**C**) Predicted 3-D structure of the proposed chimeric multiepitope vaccine, (**D**) Z-score analysis from ProSA-web, representing the overall model quality compared to experimentally determined structures.

To assemble the final chimeric multi-epitope subunit vaccine (MESV), selected immunodominant epitopes were used. These epitopes were derived from the fusion proteins of *hMPV* and *RSV*, as well as the neuraminidase of *IAV*. They were joined using specific linkers to ensure structural flexibility and optimal immune presentation (Fig. 2A). Cytotoxic T lymphocyte (CTL) epitopes were linked via AAY linkers, helper T lymphocyte (HTL) epitopes via GPGPG linkers, and B-cell epitopes via KK linkers. The AAY linker facilitates efficient proteasomal processing and MHC class I presentation. GPGPG linkers enhance the presentation and immunogenicity of HTL and B-cell epitopes. KK linkers maintain structural separation, minimizing epitope interference and boosting immunogenicity. An immunostimulatory adjuvant, human β-defensin 2, was conjugated to the N-terminus of the construct using an EAAK linker to further enhance immune activation. Human β-defensin 2 is part of the body’s natural defense system and can activate specific immune responses against antigens. The finalized MESV construct comprised 442 amino acids in total. The specific epitopes incorporated into the vaccine design are detailed in Tables 1, 2 and 3.

### 3-D structure prediction and validation

Five 3D structural models of the designed chimeric vaccine were generated, and the first model demonstrated the most stable and well-defined conformation (Fig. 2A, C). This model was selected for subsequent structural and immunological analyses. Each epitope, including CTL, HTL, and B-cell epitopes, is represented in distinct colors and depicted as a ribbon. A surface shade has been applied for enhanced depiction (Fig. 2C). The selected model was validated by ProSA-web (Fig. 2D), ERRAT, and Ramachandran plot (Fig. 2B). The ProSA-web analysis revealed a score of − 8.3 for the 3D model of the proposed vaccine design. The overall quality factor reported by ERRAT was 96.31%, indicating high model accuracy (Supplementary Fig. S4). The Ramachandran plot study indicated that the majority of amino acids (98.4%) are located in the favored zone, whilst a minimal proportion (1.6%) is found in the prohibited region (Fig. 2B). The aforementioned results demonstrated that the quality of the chosen vaccine construct designs is appropriate and suitable for the next steps, including molecular docking, modelling, and free energy calculations.

### Physicochemical properties, allergenicity, and antigenicity of the vaccine construct

The antigenic potential of the designed vaccine construct was evaluated using the VaxiJen v2.0 server. It yielded a score of 0.50, which exceeds the threshold value of 0.4 and indicates a strong capacity to produce an immune response. Allergenicity assessment was conducted using the AllerTOP v2.0 server. The construct was predicted to be non-allergenic, suggesting its potential safety in immunization applications. We used the ProtParam tool to check properties like molecular weight, half-life, aliphatic index, pI, stability, and GRAVY score. The vaccine’s molecular weight is 46,715.05 Da. Its predicted half-life is 30 h in human cells, 20 h in yeast, and over 10 h in *E. coli*, showing good stability. The instability index is 28.79, which means the construct is stable. An aliphatic index of 74.28 suggested good thermostability, while a theoretical pI supported its acidic nature. The GRAVY score of -0.276 reflected the overall hydrophilic character of the vaccine, favoring solubility and biological interaction potential (Table 4).

**Table 4:** The physicochemical properties of the designed chimeric multi-epitope subunit vaccine.

S. No	Parameter	Value	Remarks
1	Number of amino acids	442	Suitable
2	Molecular weight	46715.05	Average Weight
3	Number of atoms	6559	Satisfactory
4	Theoretical pI	6.77	Satisfactory
5	The estimated half-life (Mammalian reticulocytes, in vitro)	30 h	Satisfactory
6	The estimated half-life (yeast, in vivo)	> 20 h	Satisfactory
7	The estimated half-life (*Escherichia coli*, in vivo)	> 10 h	Satisfactory
8	Instability index	28.79	Stable
9	Aliphatic index	74.28	Satisfactory
10	GRAVY	-0.276	Satisfactory

### Molecular docking with TLR4 receptor

**Fig. 3:**
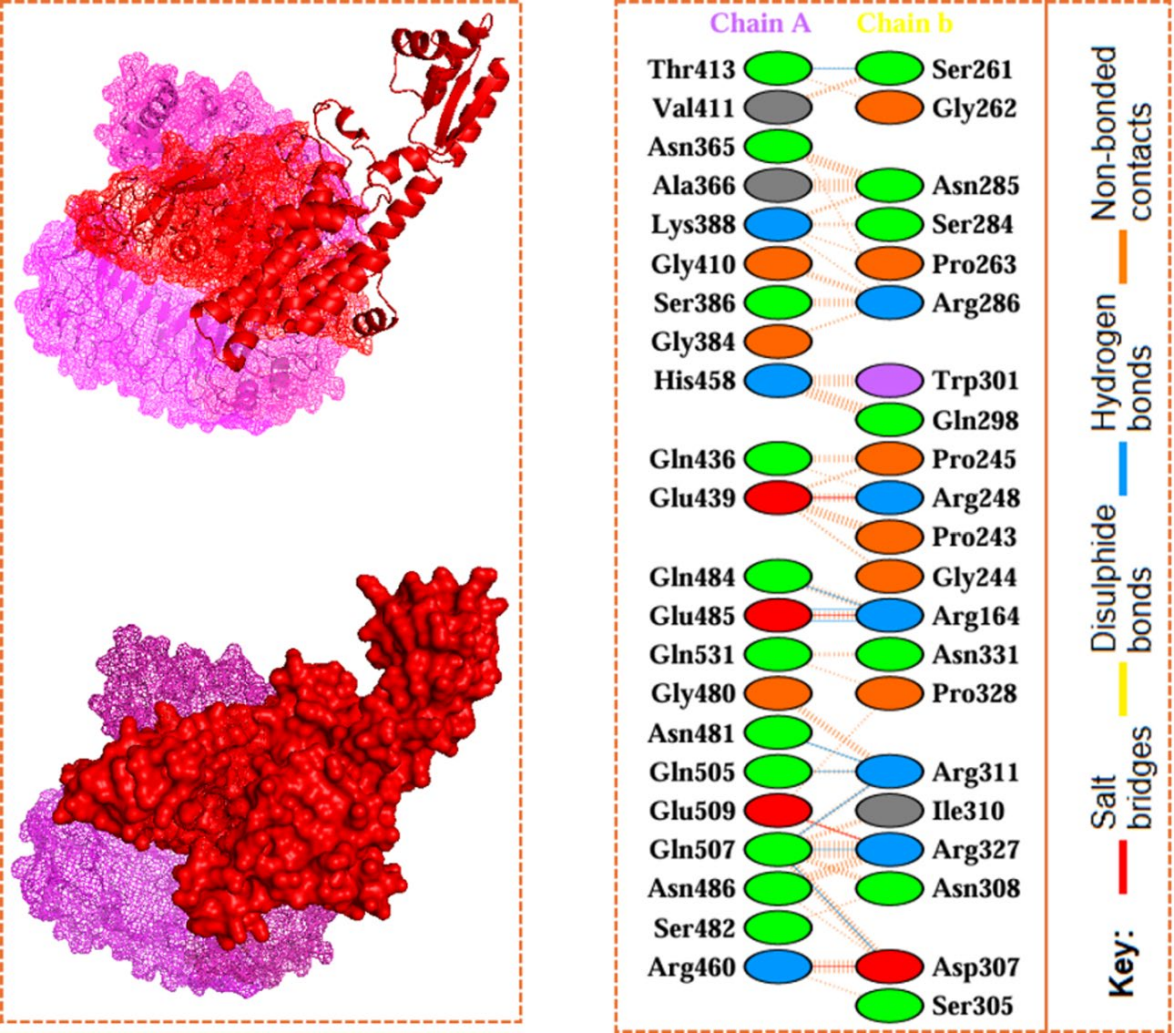
Binding interaction between the docked vaccine and TLR-4 receptor.

An effective immunological response from a vaccine depends on its high binding affinity to the host immune receptors. Toll-like receptors (TLRs) play a crucial role in controlling inflammatory pathways. They help regulate immune responses to infections. TLRs recognize pathogen-associated molecular patterns (PAMPs). This recognition initiates intracellular signaling pathways and modulates gene expression. As a result, innate and adaptive immune responses are activated^36^. Toll-like receptors play a critical role in recognizing many viral components, including nucleic acids and envelope glycoproteins. This recognition initiates a cascade of events that leads to the synthesis of type I interferons (IFN-I), inflammatory cytokines, and chemokines. TLRs help activate and mature dendritic cells, linking the body’s early (innate) and later (adaptive) immune responses. We used the HDOCK server to test how the chimeric vaccine binds to the host’s immune receptors. The docking analysis assessed the binding affinity of the vaccine construct with human Toll-like receptor 4 (TLR-4; PDB ID: 2Z63). The resulting docking score was − 277.43 kcal/mol, suggesting a strong and stable interaction between the vaccine and TLR-4, which may support effective immune activation (Fig. 3). As per the PDBsum server, TLR-4 (Chain A) and the chimeric constructed vaccine (Chain B) have 9 hydrogen bonds: Thr413-Ser261, Gln484-Arg164, Glu485-Arg164 (two hydrogen bonds), Asn481-Arg311, Gln505-Arg311, Arg311-Gln507, Gln507-Arg327, and Gln507-Asp307. Additionally, four salt bridges; Glu439-Arg248, Glu485-Arg164, Glu509-Arg327, and Arg460-Asp307, and 183 non-bonded interactions were found in the constructed vaccine and TLR4 complex (Fig. 3).

### Population coverage analysis

**Fig. 4:**
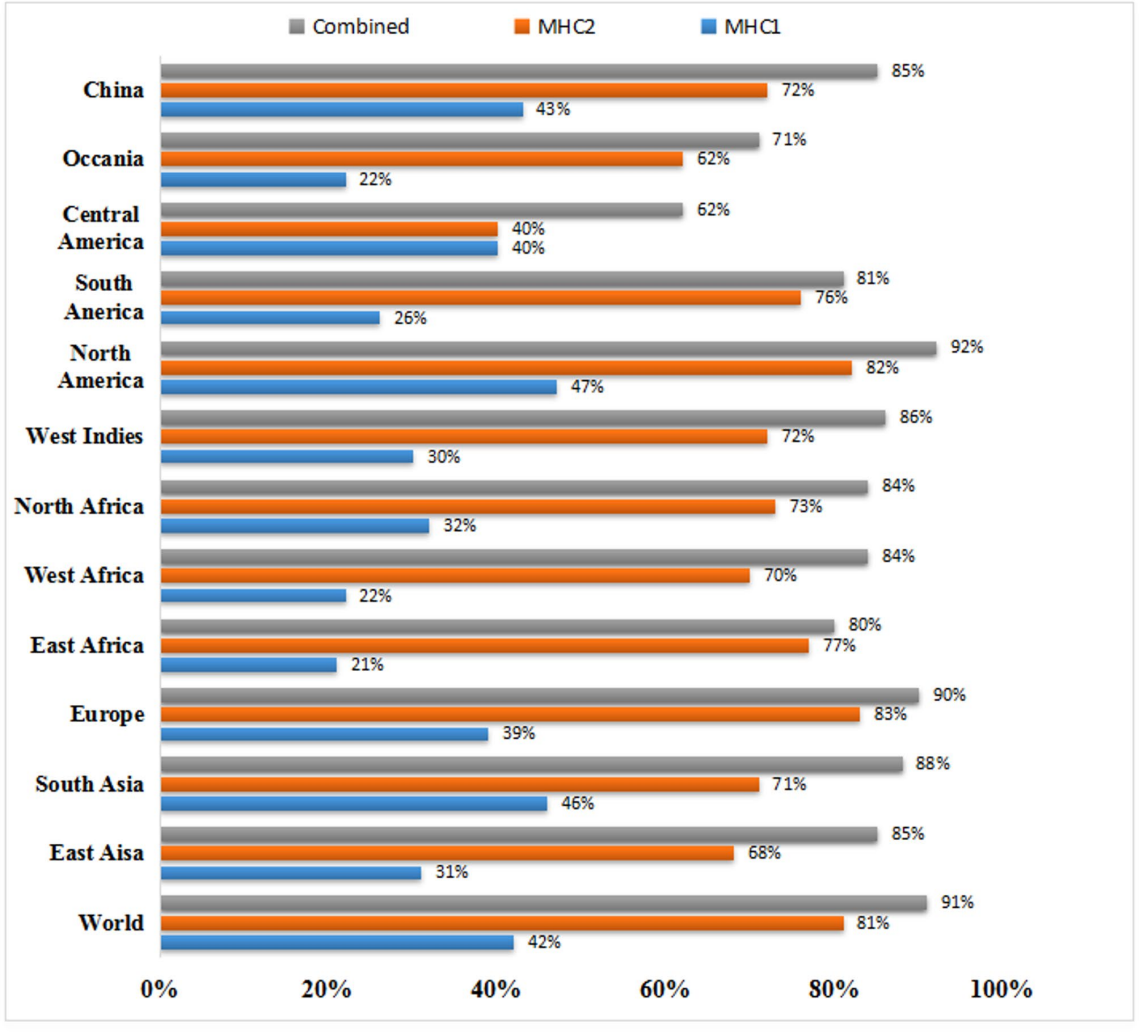
The estimated population coverage analysis of individual and combined MHC-I and MHC-II epitopes.

Population coverage analysis was performed using the IEDB Population Coverage tool. The objective was to identify the most representative epitopes across distinct immune recognition strategies. The analysis utilized curated data from the IEDB database. It accounted for regional variations in the global distribution of HLA and MHC alleles. These variations arise from complex environmental and genetic influences. Population coverage is a crucial factor in assessing the potential efficacy and broad applicability of a vaccine candidate. The IEDB analysis predicted a global coverage of 42% for MHC class I T-cell epitopes. For MHC class II T-cell epitopes, the predicted coverage was 81%. When both classes were considered together, the overall coverage increased to 91%. Figure 4 shows the regions with the highest combined epitope coverage worldwide.

### Normal mode analysis of the constructed vaccine with TLR-4

**Fig. 5:**
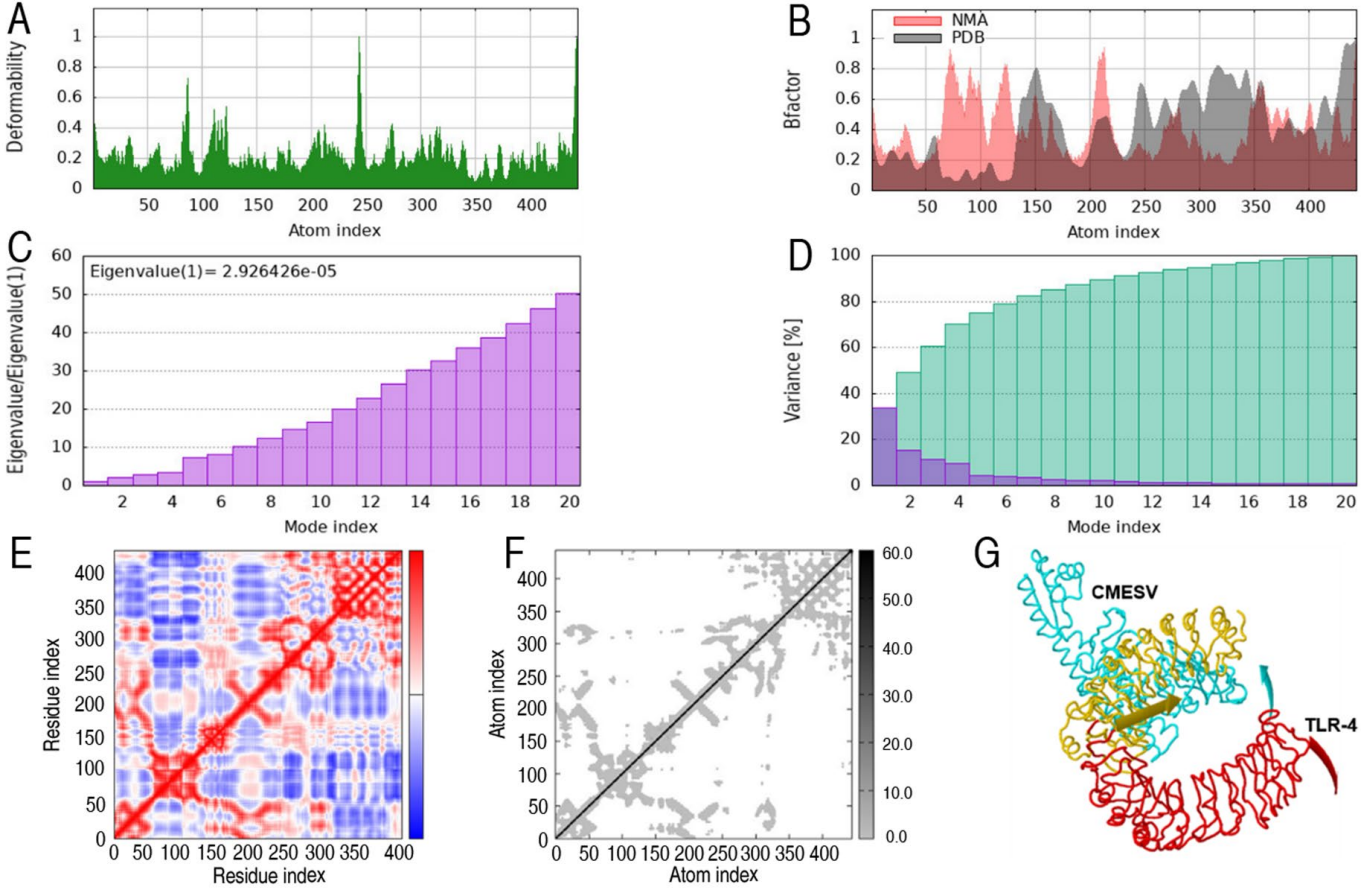
Normal mode analysis (NMA) of the chimeric multi-epitope vaccine construct in complex with TLR-4. (**A**) Deformability plot showing flexible regions within the complex. (**B**) B-factor plot comparing experimental and predicted residue fluctuations. (**C**) Eigenvalue representing the overall structural stiffness. (**D**) Variance plot showing individual and cumulative variances of the modes. (**E**) The covariance matrix of residue movements indicates correlated (red), uncorrelated (white), and anti-correlated (blue) motions. (**F**) Elastic network model illustrating inter-residue connections as springs. (**G**) Summary plot of the overall mobility and interaction dynamics derived from iMODS.

The iMODS analysis revealed reduced structural distortions and high conformational stability of the vaccine–receptor complex, indicating a stable interaction and favorable structural dynamics between the vaccine construct and the immune receptor (Fig. 4). Molecular simulations estimated the flexibility and motion of atoms within the antigen–receptor interface. Figure 5A displays the deformability profile of the MEVC-TLR4 complex, showing a localized peak within a flexible region of the vaccine build. B-factor analysis, as shown in (Fig. 5B), compares the intrinsic mobility of residues derived from both the PDB and NMA models. The eigenvalue, depicted in (Fig. 5C), reflects the stiffness of the structure; a lower value indicates greater flexibility. (Fig. 5D) presents the variance associated with individual and cumulative motions, represented in purple and green, respectively. The covariance matrix (Fig. 5E) shows the dynamic correlations between residue pairs, where red denotes correlated motion, blue indicates anti-correlated motion, and white represents uncorrelated motion. Figure 5F represents the elastic network model, resembling a spring map, which visualizes the strength of interatomic interactions within the complex. Lastly, Fig. 5G summarizes the overall results of the iMODS analysis, confirming the structural integrity and potential functional stability of the vaccine TLR4 complex.

### In silico cloning of chimeric multi-epitope subunit vaccine in the pET-28a(+) expression vector

**Fig. 6:**
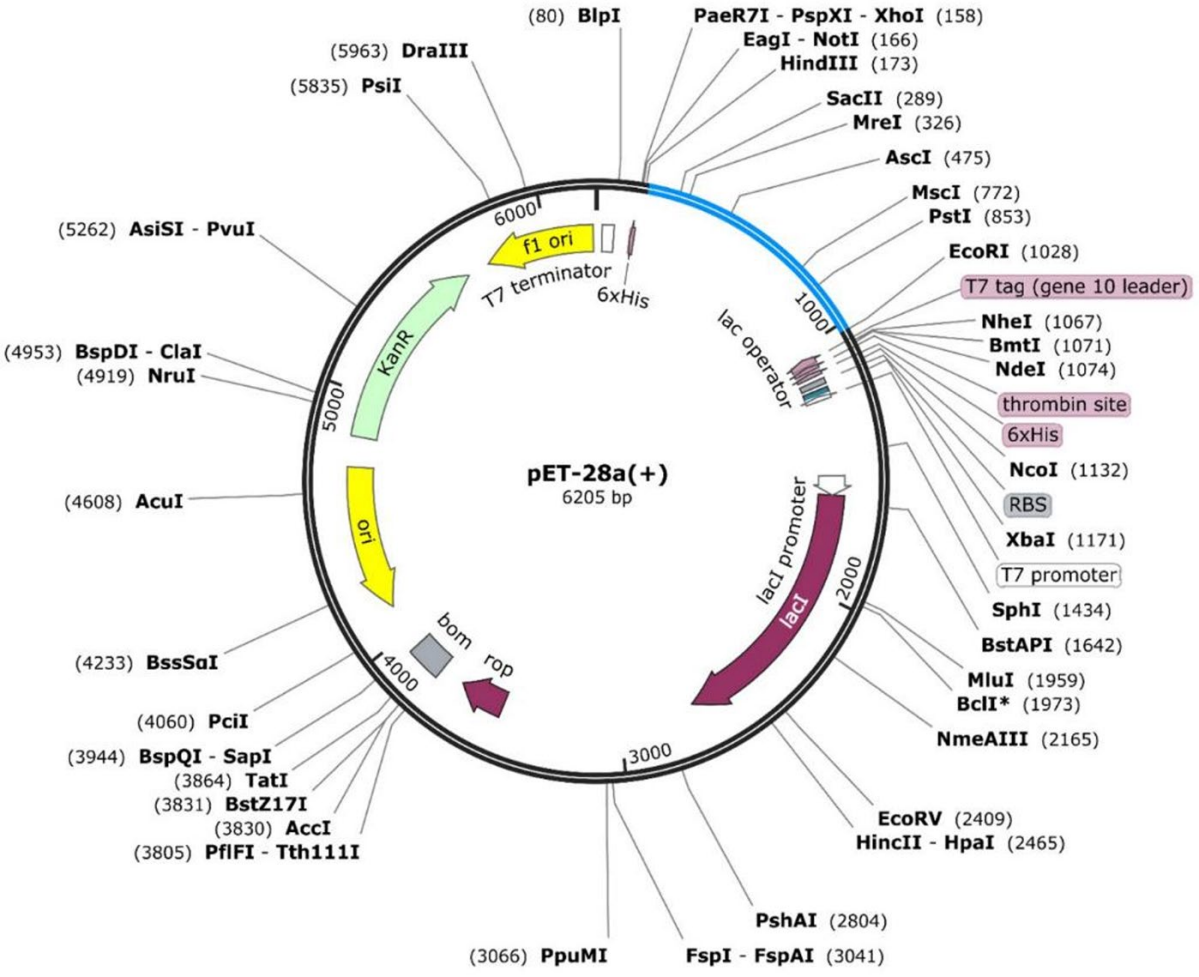
In silico cloning of the codon-optimized chimeric vaccine into the pET-28a(+) vector. The diagram shows the successful insertion of the gene using *Sca I* and *Bmt I* sites, confirming it can be expressed in *E. coli*.

The codon optimization of the 1,326-nucleotide chimeric multi-epitope subunit vaccine for *E. coli* K12 resulted in improved expression potential. The optimized sequence showed a high Codon Adaptation Index (CAI) and balanced GC content, indicating efficient transcription and translation compatibility within the selected host system. The optimized sequence yielded a CAI value of 1.0 and a GC content of 51.24%, both of which suggest high compatibility and potential for robust expression in the *E. coli* system. Subsequently, SnapGene software (v3.3.4) was used to simulate the cloning strategy. ScaI and BmtI restriction sites were added to the sequence ends to help insert it into the *E. coli* pET-28a(+) vector. The software confirmed the absence of internal restriction sites that could interfere with the cloning process. As illustrated in (Fig. 6), the optimized gene sequence was successfully integrated into the vector map, validating the construct’s readiness for downstream expression and purification.

### Immune response simulation analysis

**Fig. 7:**
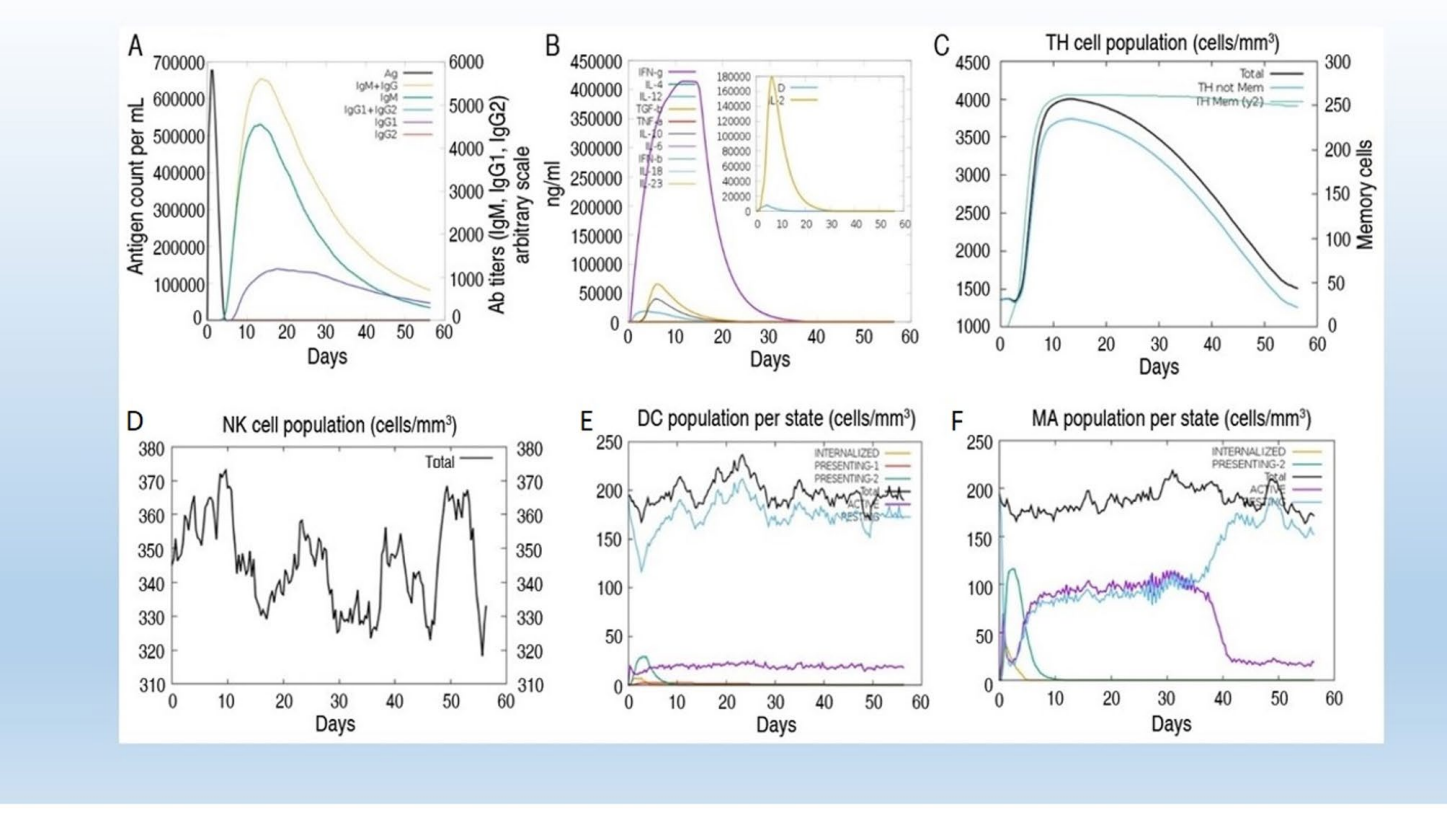
In silico immune simulation results for the constructed multi-epitope vaccine, generated using the C-ImmSim server. (**A**) Primary, secondary, and tertiary immune responses following the 3-D vaccine show elevated levels of IgM, IgG1, and IgG2 antibodies. (**B**) B cell population dynamics, including memory B cell generation and clonal expansion. (**C**) T-helper (CD4^+^) and cytotoxic T lymphocyte (CD8^+^) response profiles, including active and memory cell populations. (**D**) Cytokine expression levels (e.g., IFN-γ, IL-2) indicate activation of cell-mediated immunity. (**E**) Macrophage and dendritic cell activation over time, reflecting innate immune involvement. (**F**) Macrophage population in response to the vaccine. The simulation supports the immunogenic potential of the vaccine construct in eliciting strong, long-lasting humoral and cellular immune responses.

Vaccines provoke the immune system of the host without causing disease. The immune system can be stimulated by vaccines through different mechanisms. Co-administration of protein-based antigens with immunologic adjuvants enhances local innate immune activation, leading to the functioning of antigen-presenting cells and the release of pro-inflammatory cytokines by macrophages. These macrophages, often referred to as dendritic cells, internalize the antigen and present it on their surface via major histocompatibility complex (MHC) molecules. T cells recognize the MHC-antigen complex through their T-cell receptors, thereby activating adaptive immunity and generating memory T cells. In contrast, polysaccharide antigens typically induce antibody responses without T-cell involvement. These antigens bind directly to mature B cells, initiating their differentiation into antibody-producing plasma cells. Immune simulation was conducted to assess the immunogenicity of the constructed vaccine. In the C-ImmSim simulation, three injections were administered at time steps 1, 84, and 168, corresponding biologically to days 0, 28, and 56, respectively, assuming each time step represents 8 h of real time. This simulation evaluated the immune system’s response, particularly antibody production, following the administration of the constructs. Notably, the immune system exhibited a robust response, with significantly elevated antibody production observed after each vaccine injection (Fig. 7). The chimeric multiepitope subunit vaccine construct was administered, and antigen titers were initially low until the seventh day. Subsequently, they progressively increased, reaching their maximum on the 12th day. Nevertheless, a rise in antibody titers was observed beginning on the 15th day. The activation of supplementary immune system components was accompanied by the complete neutralisation of antigens by the 17th day. The combined IgM and IgG titers were 4.5 × 10^7^, and the combined IgG1 + IgG2 and IgG1 titers were also elevated post-vaccination (Fig. 7A). The evaluation of interleukin (IL) and cytokine responses, as shown in (Fig. 7B), indicates a notable increase in the levels of IFN-γ and IL-2. The results demonstrate a reliable and strong immune response subsequent to the administration of the vaccine. The cellular immune response upon re-exposure to pathogens was significantly robust, marked by the development of memory cells. T cell populations were found to be greater than 1500 cells/mm³, with peak concentrations of phagocytic natural killer cells, dendritic cells, and phagocytic macrophages reported at levels exceeding 380 cells/mm^3^ and 200 cells/mm^3^, respectively (Fig. 7B–F).

### Molecular synamics simulation analysis of the TLR4-vaccine complex

**Fig. 8:**
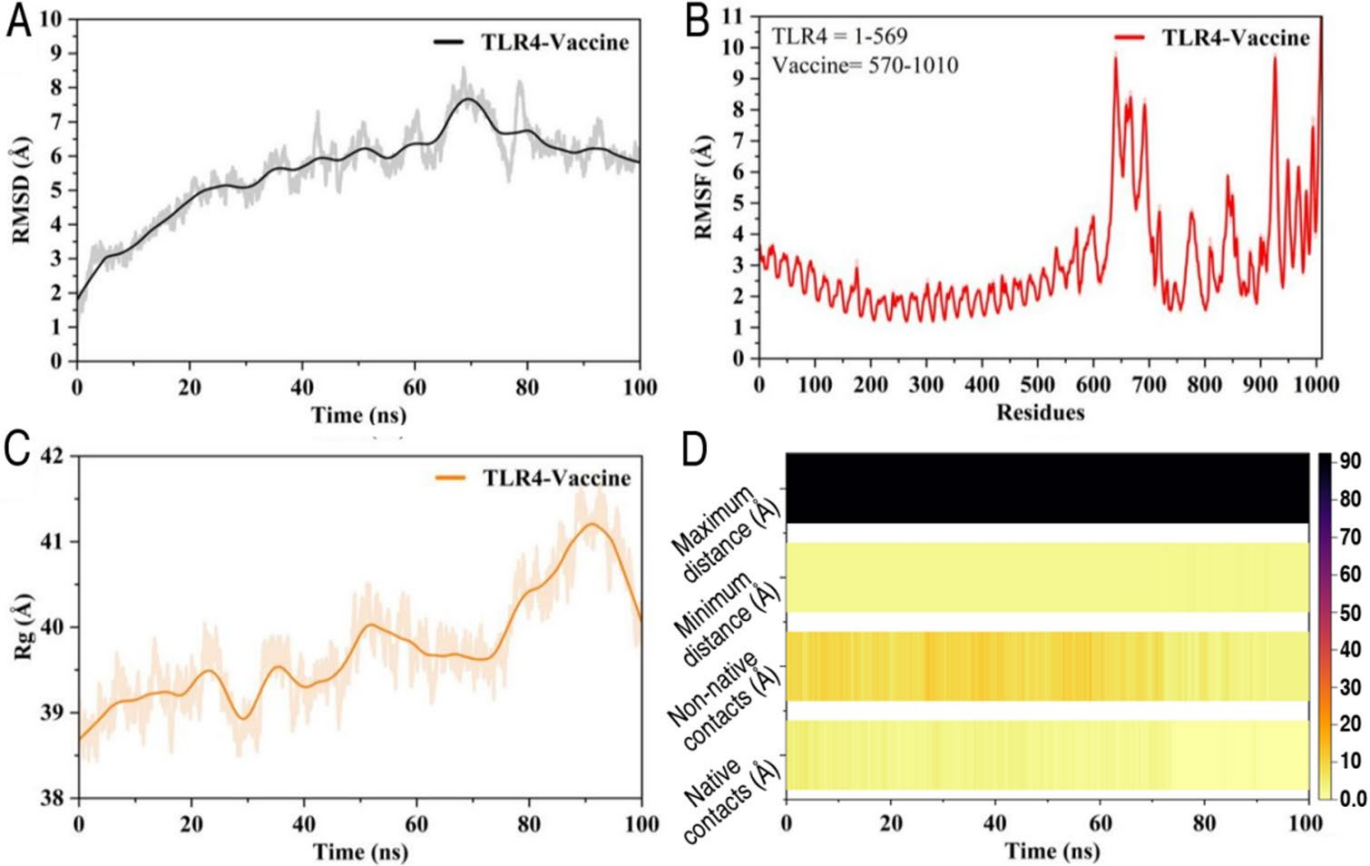
Structural stability and conformational analysis of the TLR4-vaccine complex during 100 ns molecular dynamics simulation. (**A**) Root mean square deviation (RMSD) of backbone atoms, showing system equilibration after ~ 65 ns. (**B**) Root mean square fluctuation (RMSF) per residue, highlighting localized flexibility in the vaccine region (residues 570–1010). (**C**) Radius of gyration (Rg), indicating overall compactness with slight expansion after 75 ns. (**D**) Contact analysis, showing stable native contacts and consistent minimum distances, with no significant unfolding events detected throughout the simulation.

To evaluate the structural stability and conformational behavior of the TLR4-vaccine complex, several parameters were monitored across the 100 ns production phase (Fig. 8A-D) and (Table S8). The RMSD profile (Fig. 8A) was used to assess global stability over time. The system showed a gradual rise in RMSD during the first 60 ns, reflecting initial structural adjustments. After ~ 65 ns, RMSD values stabilized around 5–6 Å, indicating that the complex reached equilibrium. Minor fluctuations observed after 70 ns suggest localized rearrangements without global instability. RMSF analysis (Fig. 8B) captured residue-wise flexibility across the complex. The TLR4 region (residues 1–569) exhibited limited fluctuations, mostly under 3 Å, indicating a stable backbone throughout the simulation. In contrast, the vaccine region (residues 570–1010) showed increased flexibility, with peaks exceeding 9 Å at certain loop regions, likely reflecting inherent flexibility in the designed construct. The Rg (Fig. 8C) was calculated to monitor the compactness of the TLR4-vaccine complex. The Rg remained steady around 39–40 Å during the initial 70 ns, suggesting stable folding. A gradual increase after 75 ns, reaching ~ 41.5 Å, indicates slight structural expansion, possibly due to flexible segments in the vaccine construct. No abrupt changes were observed, supporting overall structural retention. A contact map analysis (Fig. 8D) tracked native and non-native contacts, minimum/maximum distances, and contact stability across the trajectory. Native contacts remained consistently populated throughout, while non-native contacts fluctuated without showing dominance, suggesting preserved structural integrity. Maximum distance trends remained stable, with no significant expansion or dissociation events during the simulation. Collectively, RMSD stabilization after 65 ns, stable Rg, maintained native contacts, and localized flexibility observed in RMSF profiles indicate that the TLR4-vaccine complex remained structurally stable under simulated conditions, with expected mobility confined to vaccine regions.

### Conformational dynamics and principal component analysis

**Fig. 9:**
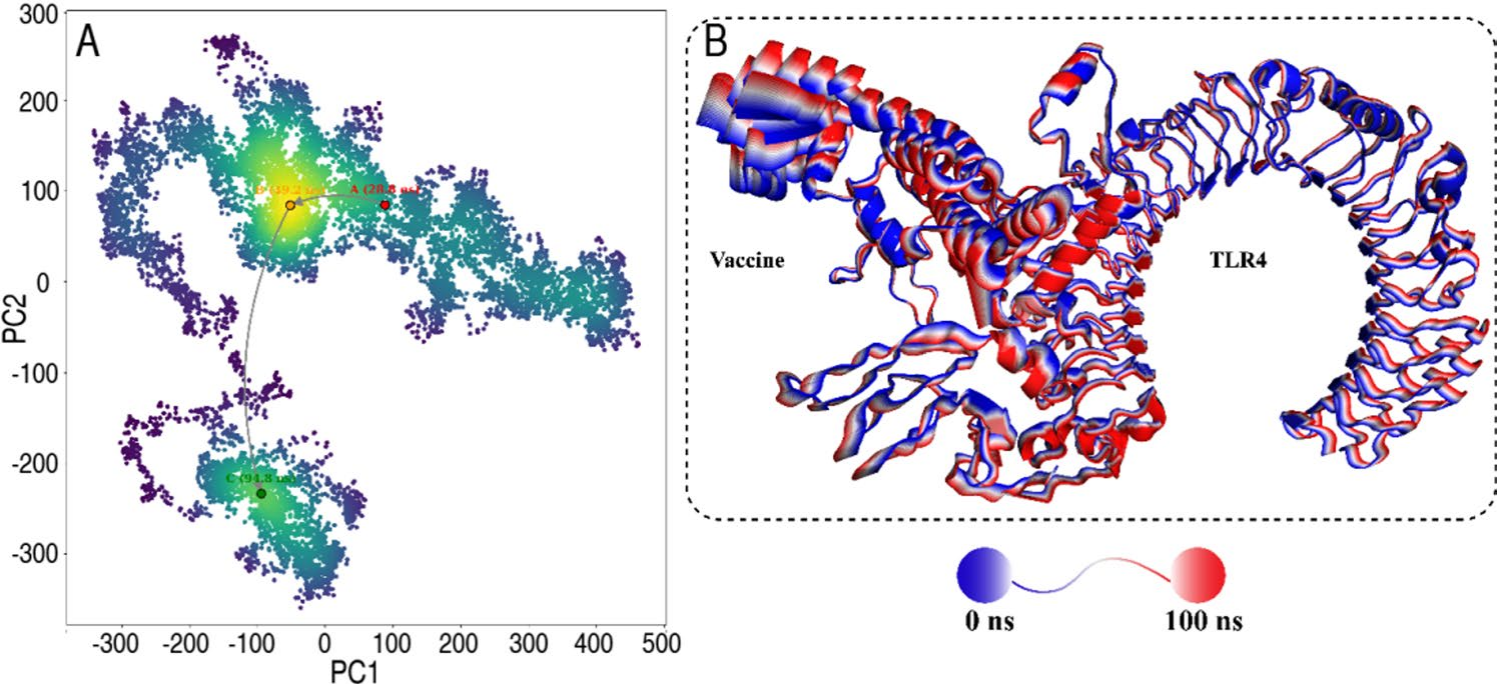
(**A**) Principal component analysis (PCA) projection of the TLR4-vaccine complex along PC1 and PC2 during the 100 ns MD simulation. Three distinct conformational states (A: 28.3 ns, B: 49.2 ns, C: 94.8 ns) are indicated. (**B**) Structural superposition of the complex at 0 ns (blue) and 100 ns (red), highlighting the overall structural preservation, with minor adjustments localized to the vaccine region.

Principal component analysis (PCA) was conducted to investigate large-scale conformational changes in the TLR4-vaccine complex over the course of the simulation (Fig. 9A). The trajectory was projected along the first two principal components (PC1 and PC2), which captured the dominant motions within the system. The PCA plot revealed three distinct conformational states sampled during the simulation. The first state (A) was identified at ~ 28.3 ns, the second state (B) at ~ 49.2 ns, and the third state (C) at ~ 94.8 ns. The transitions between these states suggest gradual exploration of the conformational space rather than abrupt structural shifts. The presence of distinct clusters indicates that the complex underwent coordinated motions but remained within a restricted conformational landscape, supporting the structural stability observed in RMSD and Rg analyses. To visualize structural changes, representative snapshots corresponding to the early (blue) and late (red) stages of the simulation were superimposed (Fig. 9B). This comparison showed minimal deviation in the TLR4 region, consistent with its structural rigidity, while subtle shifts were observed in the vaccine region, reflecting expected flexibility. The PCA confirmed that the TLR4-vaccine complex explored a limited conformational space during the simulation, without significant structural rearrangement, supporting the stability of the complex under simulated conditions.

### Binding free energy analysis of the TLR4-vaccine complex

The binding free energy of the TLR4-vaccine complex was estimated using the Molecular Mechanics Generalized Born Surface Area (MM/GBSA) method based on snapshots from the production phase. The calculated energy components are summarized in Table 5. The total binding free energy (ΔG_TOTAL_) was estimated as − 121.72 kcal/mol, indicating a stable and favorable interaction between the vaccine construct and TLR4. The major contribution came from electrostatic interactions (ΔE_EL_: − 378.08 kcal/mol) and van der Waals forces (ΔE_VDW_: − 64.00 kcal/mol). The non-polar solvation term (ΔE_SASA_: − 8.85 kcal/mol) contributed marginally to stabilization. In contrast, the polar solvation energy (ΔEGB: 329.22 kcal/mol) opposed binding, as expected due to desolvation penalties upon complex formation. Despite this, the gas-phase interaction energy (ΔG_GAS_: − 442.09 kcal/mol) effectively compensated, resulting in an overall favorable binding profile. The low standard error values across all components confirmed the stability and consistency of the calculated energy terms throughout the sampled frames. Overall, MM/GBSA analysis supports strong binding affinity between the multi-epitope vaccine construct and TLR4, driven primarily by electrostatic and van der Waals interactions.

**Table 5:** MM/GBSA binding free energy components for the TLR4-vaccine complex. Energies are reported as average values (kcal/mol) with standard deviation and standard error.

Energy Component	Average	Standard deviation	Standard error
ΔE_VDW_ (kcal/mol)	− 64.00	11.91	0.37
ΔE_EL_ (kcal/mol)	− 378.08	44.13	1.39
ΔE_GB_ (kcal/mol)	329.22	42.51	1.34
ΔE_SASA_ (kcal/mol)	− 8.85	1.94	0.06
ΔG_GAS_ (kcal/mol)	− 442.09	45.13	1.42
ΔG_SOLV_ (kcal/mol)	320.36	42.25	1.33
ΔG _TOTAL_ (kcal/mol)	− 121.72	8.24	0.26
ΔE_VDW_, van der Waals free energy; ΔE_EL_, Electrostatic free energy; ΔE_GB_, The polar component of solvation-free energy; ΔE_SASA_, Non-polar components of solvation energy; ΔG_GAS_, Binding free energy without solvent; ΔG_SOLV_, Binding free energy with solvent; ΔG_TOTAL_, Total binding free energy.			

## Discussion

The growing burden of multidrug-resistant pathogens highlights the urgent need for novel preventive strategies, including effective vaccines^37^. In this context, the use of predicted epitopes enables the rational design of multi-epitope vaccines that address key limitations of conventional single-peptide vaccines, such as limited immune coverage and suboptimal immunogenicity^38^. Combining multiple B-cell and T-cell epitopes within a single vaccine construct is envisioned to promote a broader engagement of the adaptive immune system, encompassing both humoral and cellular responses^39,40^. In contrast to currently licensed monovalent or bivalent respiratory vaccines, which are based on whole antigens or inactivated pathogens and often drive immunity toward a limited set of dominant surface epitopes, multi-epitope strategies seek to focus immune recognition on conserved regions. Such a design may offer advantages in terms of cross-reactivity and reduced vulnerability to antigenic drift. The success of such an approach relies on careful epitope selection, sufficient HLA coverage, and appropriate immunostimulatory support to ensure effective immune activation in the absence of native protein structure^41–43^. The approach enables modular updating and recombinant scalability, while the absence of native antigen architecture requires optimized antigen presentation and adjuvant-mediated immune activation to achieve immunogenicity comparable to conventional vaccine platforms^41^.

In this study, a structure-based immunoinformatics framework was used to design a multi-epitope vaccine targeting Human Metapneumovirus, Respiratory Syncytial Virus, and Influenza A Virus. The final construct incorporated cytotoxic T-lymphocyte (CTL), helper T-lymphocyte (HTL), and B-cell epitopes with high predicted antigenicity, strong MHC binding affinity, and broad population coverage, suggesting the potential to induce coordinated cellular and humoral immunity^44^. The inclusion of an N-terminal adjuvant significantly enhanced the predicted immune profile, as evidenced by elevated IFN-γ, IgG, and memory T-cell responses in immune simulations^38–40^. Flexible linkers further improved epitope accessibility and construct stability, as supported by secondary and tertiary structural analyses^41^. The vaccine also exhibited favorable physicochemical properties, including predicted stability, non-allergenicity, and solubility, indicating feasibility for recombinant production and downstream experimental validation.

Accurate structural modeling is critical for predicting immune recognition and receptor binding in multi-epitope vaccines^8^. However, the chimeric construct lacks a close structural homolog, classical homology-based modeling is inherently unreliable. When sequence identity falls below the 30% “twilight zone,” alignment errors increase sharply, and both core folds and loop regions may be mis-predicted^45^. Recent benchmarks confirm that under these conditions, homology models are unsuitable for atomic-level interpretation, necessitating the use of threading or hybrid de novo approaches^46^. In this study, the refined tertiary structure showed acceptable stereochemical quality, supporting the likelihood that CTL, HTL, and B-cell epitopes remain accessible for immune recognition.

To estimate innate immune activation potential, the interaction between the vaccine construct and human Toll-like receptor 4 (TLR4) was evaluated. Docking analysis indicated that the vaccine engages TLR4 in a manner consistent with known ligand–receptor recognition mechanisms^44^, which is biologically relevant because TLR4 activation drives dendritic cell maturation, cytokine secretion, and T-cell priming. Because docking provides only a static view, molecular dynamics (MD) simulations were used to evaluate the stability and behavior of the vaccine–TLR4 complex under near-physiological conditions.

The MD trajectory demonstrated that the complex stabilized after ~ 65 ns, with no major structural rearrangements thereafter^8^. The consistent radius of gyration indicated maintenance of compactness, while RMSF analysis showed rigidity of the TLR4 chain and expected flexibility within vaccine linker and loop regions. Contact analysis confirmed preservation of native interactions, and principal component analysis identified only three dominant conformational states, indicating restricted and coordinated motion within a stable conformational landscape^34^. Together, these results support the structural robustness of the vaccine TLR4 interaction.

Binding free energy calculations using MM/GBSA further supported these findings. Although MM/GBSA is known to overestimate absolute affinities due to simplified solvation and entropy treatment, its use across multiple MD snapshots improves the reliability of relative binding trends^47^. The total binding energy was favorable, driven primarily by electrostatic and van der Waals interactions, despite counteracting polar solvation effects, consistent with known mechanisms of TLR4–peptide recognition^46,47^. These results indicate that the vaccine forms a stable and energetically favorable complex with TLR4, supporting its potential to function as an immunostimulatory agent.

Population-coverage analysis using IEDB revealed high global and regional HLA representation driven by prevalent alleles such as HLA-A02:01, HLA-A24:02, and HLA-B07:02 (MHC-I), together with HLA-DRB1*04:01*,* DRB1*07:01, and DRB1*15:01 (MHC-II), which dominate antigen presentation across Europe, East Asia, South Asia, and the Americas^48^. However, reduced representation of certain HLA-B and HLA-DR alleles common in West and East African and indigenous populations suggests a potential coverage gap, highlighting the need for additional epitopes to improve global equity^49^.

The immunogenic potential of the construct was further evaluated using C-ImmSim immune simulations. The predicted induction of class-switched antibodies and Th1-associated cytokines, including IFN-γ and IL-2, indicates activation of immune pathways associated with viral clearance and memory formation^50,51^. These findings suggest that the construct promotes a balanced humoral and cellular response, which is essential for durable protection against viral infections. However, molecular-level simulations do not capture tissue-specific immunity. MD modeling evaluates epitope accessibility and receptor binding but cannot distinguish systemic IgG responses from mucosal IgA-dominated immunity, which is critical for respiratory pathogens^8^. Likewise, C-ImmSim provides qualitative and semi-quantitative insights into immune dynamics but cannot fully represent host-pathogen interactions, immune evasion, or organ-specific responses^52,53^. Therefore, in vivo and ex vivo validation remains essential.

From a translational perspective, favorable codon adaptation, in-silico cloning, and predicted solubility suggest feasibility for recombinant expression, particularly in *E. coli*. Nevertheless, computational solubility correlates only moderately with experimental outcomes and is influenced by host-specific folding, stress responses, and post-translational effects^54,55^. Alternative expression systems may therefore be required. Despite these limitations, similar computational pipelines have successfully produced viable vaccine candidates, validating this approach as a powerful early-stage screening strategy^56,57^.

Overall, the integration of epitope selection, structural modeling, molecular docking, molecular dynamics simulation, immune simulation, and population-coverage analysis provides a comprehensive in-silico validation of this multi-epitope vaccine candidate. By targeting conserved regions of the fusion proteins of hMPV and RSV and conserved neuraminidase regions of IAV, the construct is designed to elicit both neutralizing antibodies and cross-reactive T-cell responses, potentially enabling broader and more durable protection than single-pathogen designs^53–57^. These results provide a strong rationale for advancing this candidate to experimental validation.

## Conclusion

Emerging respiratory viral co-infections caused by *hMPV*, *RSV*, and *IAV* remain a significant public health concern, especially in vulnerable populations. In this study, a multi-epitope vaccine was designed using a structure-based immunoinformatics approach to target these pathogens. The designed construct incorporated carefully selected CTL, HTL, and B-cell epitopes, along with an appropriate adjuvant, aiming to stimulate both innate and adaptive immunity. Structural modeling and molecular docking confirmed favorable binding to human TLR4, while molecular dynamics simulations demonstrated the stability of the vaccine-receptor complex under physiological conditions. Binding free energy analysis supported a strong interaction, primarily driven by electrostatic and van der Waals forces. In silico immune simulation predicted robust immune activation, and codon optimization confirmed the construct’s suitability for expression in *Escherichia coli*. These results support the designed multi-epitope vaccine as a promising candidate for experimental validation and further development.

### Limitations and future directions

Designing a multi-epitope vaccine targeting multiple pathogens entails several significant challenges. One of the primary concerns is achieving an optimal balance of immunogenicity among diverse antigenic components to prevent potential immune interference. Additionally, ensuring broad human leukocyte antigen (HLA) coverage while minimizing the risk of autoimmune cross-reactivity is critical for achieving population-wide efficacy and safety. Furthermore, the development of a stable vaccine formulation that maintains consistent epitope expression and minimizes antigen degradation or loss during manufacturing and storage remains a considerable technical hurdle. While our study provides an in-silico assessment of the designed multi-epitope vaccine, experimental validation remains essential to confirm its immunogenicity and safety. The structural stability, binding affinity, and immune stimulation potential demonstrated computationally should now be verified through in vitro assays, such as protein expression, receptor binding, and cytokine profiling. Further in vivo studies will be required to evaluate immunogenicity, protective efficacy, and safety under physiological conditions. Experimental validation will be essential to confirm the immunogenicity and protective efficacy of the designed vaccine. Nonetheless, this computational framework provides an efficient strategy for accelerating early-stage vaccine discovery and candidate selection.

## Supplementary Information

Below is the link to the electronic supplementary material.

Supplementary Material 1

Supplementary Material 2

## Data Availability

All data is publicly available on the FDA website, the original contributions presented in the study are included in the article/Supplementary Material. Further inquiries can be directed to the corresponding author.
