# Supplementary material for: Immunoinformatics based designing of a broad-spectrum multi-epitope vaccine against co-infection of human metapneumovirus, respiratory syncytial virus, and influenza A virus
Source: Sci Rep. 2026 Feb 23;16:10244. doi: 10.1038/s41598-026-40812-z (PMC13031529; doi:10.1038/s41598-026-40812-z)
Supplement: Supplementary file 1 — Supplementary Material 1 [file 41598_2026_40812_MOESM1_ESM.pdf]

Immunoinformatics based designing of a Broad-Spectrum Multi-Epitope Vaccine against co-infection of *human Metapneumovirus, Respiratory Syncytial Virus*, and *Influenza A Virus*

Lu Li[1, #], Yong Chen[2, #], Shaoyong Wu[3, #], Chunyan Wu[1], Junhong Xie[1], Abdullah Shah[3], Xin Xie[1], Junyin Tan[1], Yudie Qin[1], Yuanlei Zeng[1], Amin Ullah Jan[4,*], Tianci Yang[5,*], and Sadeeq Ullah[1, *]

[1] Guangdong Provincial Key Laboratory of Medical Immunology and Molecular Diagnostics, The Second Affiliated Hospital, School of Medical Technology, Guangdong Medical University, Dongguan 523808, China

[2] Department of Medical Laboratory, Affiliated Cancer Hospital of Chengdu Medical College, Chengdu Seventh People's Hospital, Chengdu 610231, China

[3] Department of Biochemistry, University of Veterinary and Animal sciences Swat (UVAS-Swat), Swat 01923, Pakistan

[4] Department of Biotechnology, Shaheed Benazir Bhutto University Sheringal Dir Upper, Sheringal 18300, Pakistan

[5] Institute of Infectious Disease, School of Medicine, Xiamen University, Xiamen 361102, China

# These authors contributed equally to this work.

*** Correspondence:**

Sadeeq Ullah, anjum_sadiq@yahoo.com

Amin Ullah Jan, aminjan@sbbu.edu.pk

Tianci Yang, yangtianci@xmu.edu.cn

Supplementary figures:

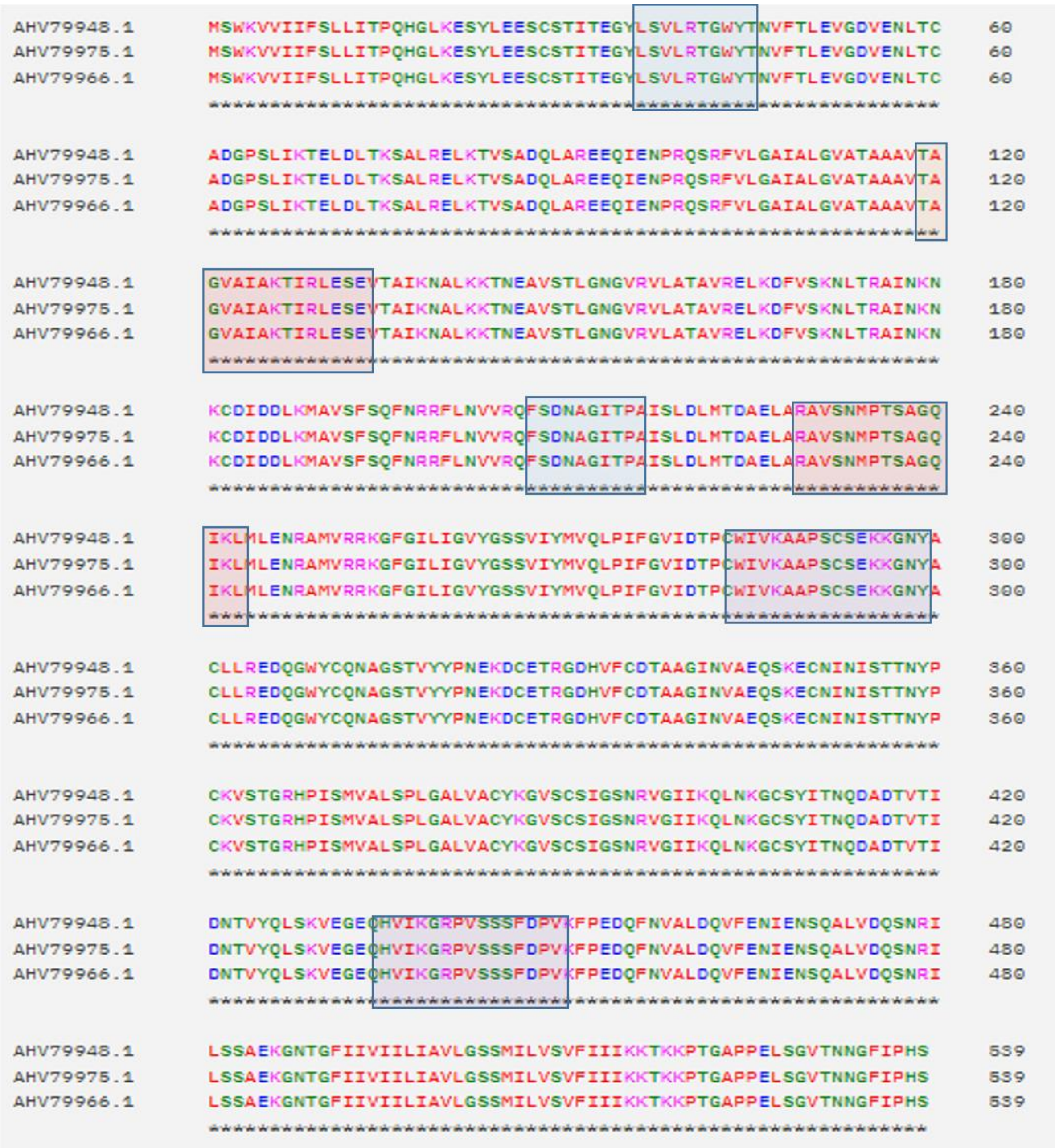

Figure S1. Shows the CTL, HTL and BCL epitopes in *human Metapneumovirus*

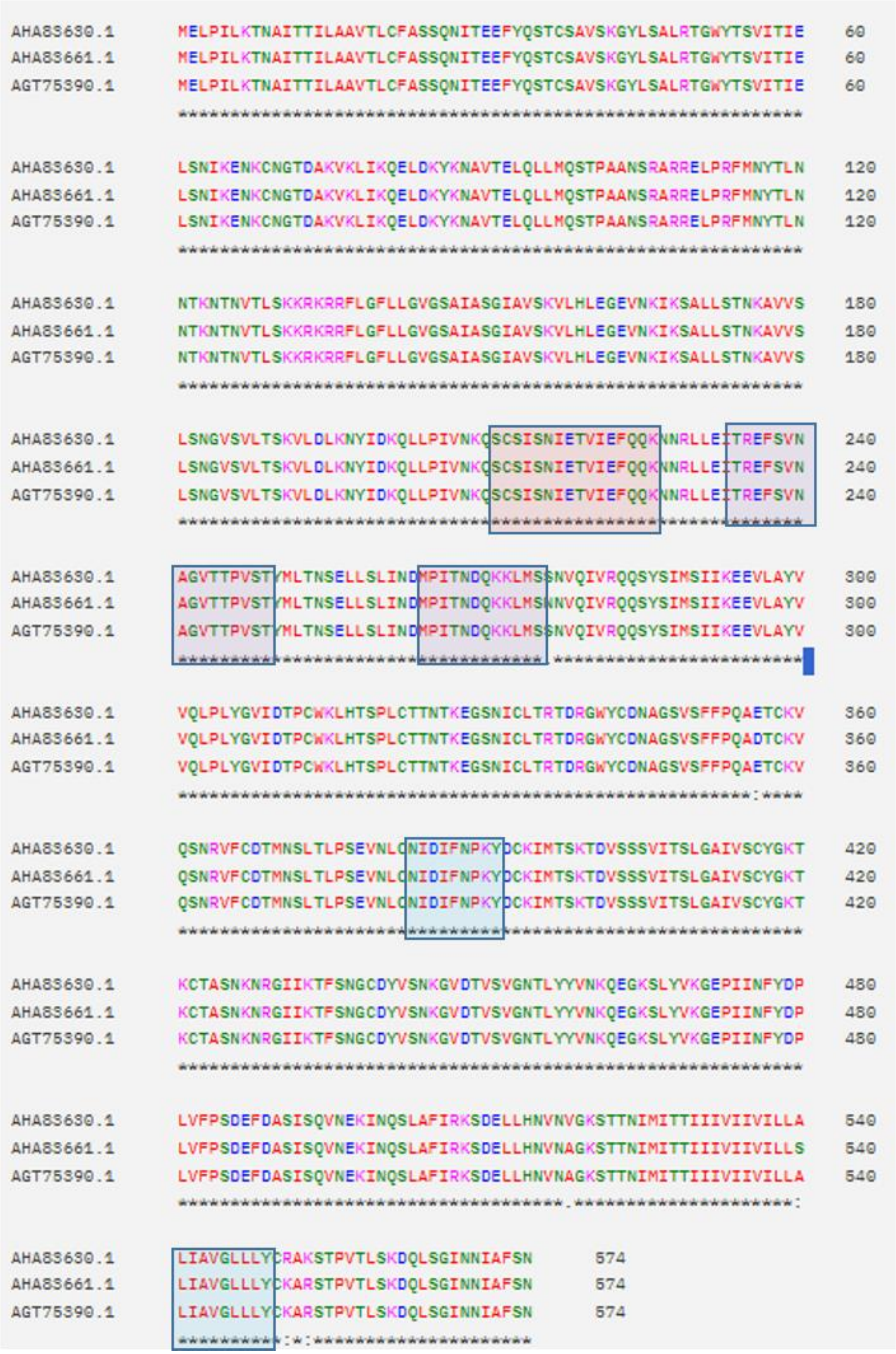

Figure S2. Shows the CTL, HTL and BCL epitopes in *Respiratory Syncytial Virus*

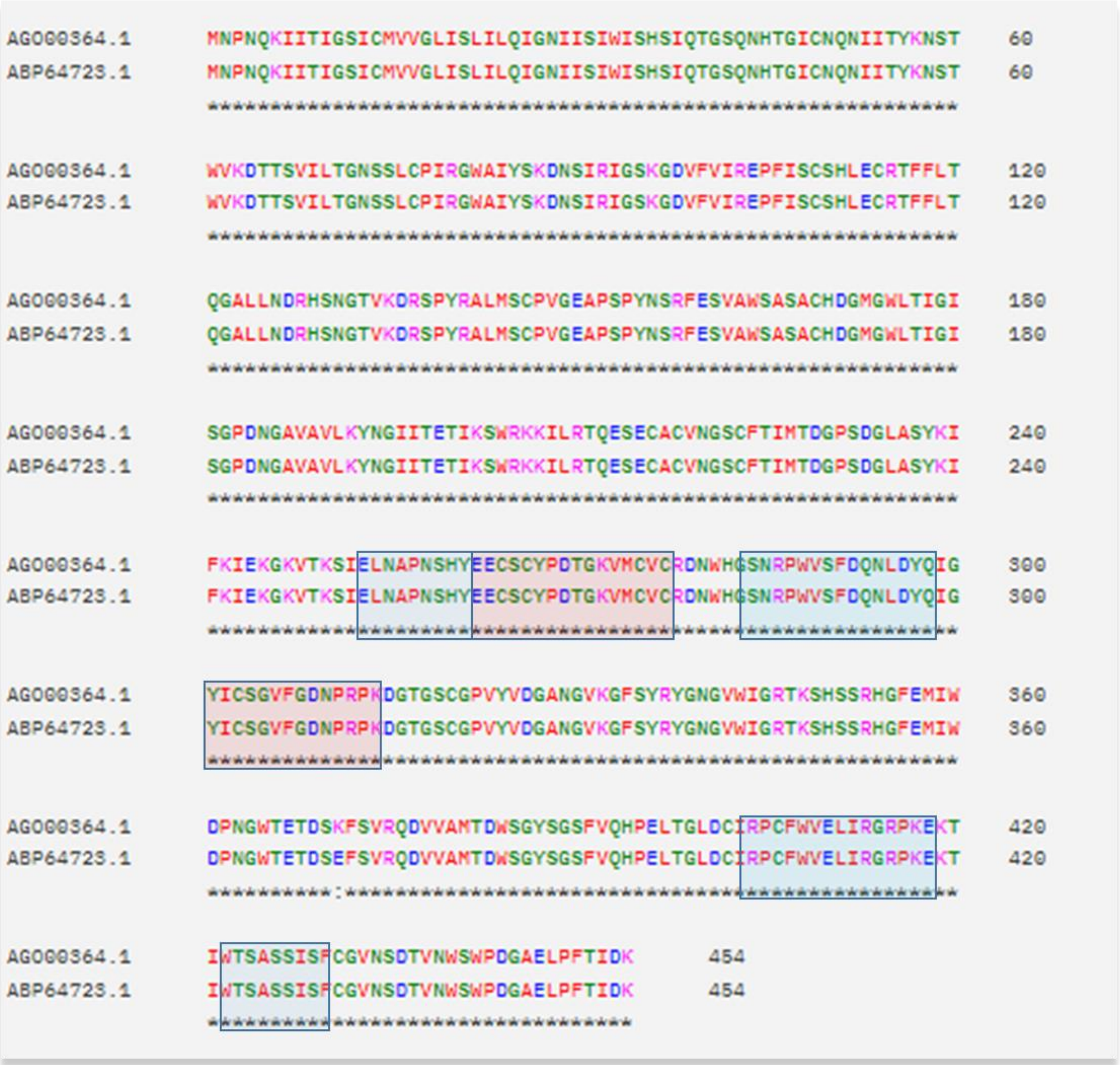

Figure S3. Shows the CTL, HTL, and BCL epitopes in the *Influenza A Virus*

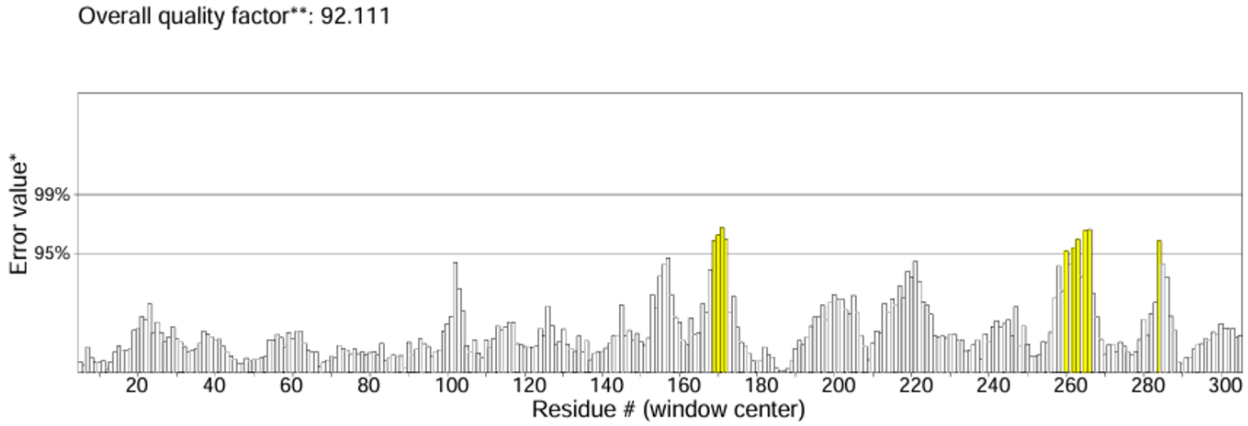

Figure S4. Shows the overall quality of the construct
