# Supplementary material for: Immunoinformatics based designing of a broad-spectrum multi-epitope vaccine against co-infection of human metapneumovirus, respiratory syncytial virus, and influenza A virus
Source: Sci Rep. 2026 Feb 23;16:10244. doi: 10.1038/s41598-026-40812-z (PMC13031529; doi:10.1038/s41598-026-40812-z)
Supplement: Supplementary file 2 — Supplementary Material 2 [file 41598_2026_40812_MOESM2_ESM.pdf]

Immunoinformatics based designing of a Broad-Spectrum Multi-Epitope Vaccine against co-infection of *human Metapneumovirus, Respiratory Syncytial Virus*, and *Influenza A Virus*

Lu Li[1, #], Yong Chen[2, #], Shaoyong Wu[3, #], Chunyan Wu[1], Junhong Xie[1], Abdullah Shah[3], Xin Xie[1], Junyin Tan[1], Yudie Qin[1], Yuanlei Zeng[1], Amin Ullah Jan[4,*], Tianci Yang[5,*], and Sadeeq Ullah[1, *]

[1] Guangdong Provincial Key Laboratory of Medical Immunology and Molecular Diagnostics, The Second Affiliated Hospital, School of Medical Technology, Guangdong Medical University, Dongguan 523808, China

[2] Department of Medical Laboratory, Affiliated Cancer Hospital of Chengdu Medical College, Chengdu Seventh People's Hospital, Chengdu 610231, China

[3] Department of Biochemistry, University of Veterinary and Animal sciences Swat (UVAS-Swat), Swat 01923, Pakistan

[4] Department of Biotechnology, Shaheed Benazir Bhutto University Sheringal Dir Upper, Sheringal 18300, Pakistan

[5] Institute of Infectious Disease, School of Medicine, Xiamen University, Xiamen 361102, China

# These authors contributed equally to this work.

*** Correspondence:**

Sadeeq Ullah, anjum_sadiq@yahoo.com

Amin Ullah Jan, aminjan@sbbu.edu.pk

Tianci Yang, yangtianci@xmu.edu.cn

Supplementary tables:

Table S1. *Respiratory Syncytial Virus* Fusion Protein

>AGT75335.1<br>MELPILKTNAITTIFAAVTLCFASSQNITEEFYQSTCSAVSKGYLSALRTGWYTSVITIELSNIKENKCN<br>GTDAKVKLIKQELDKYKNAVTELQLLMQSTPAANNRARRELPRFMNYTLNNTKNNNVTLSKKRKRRFLGF<br>LLGVGSAIASGIAVSKVLHLEGEVNKIKSALLSTNKAVVSLSNGVSVLTSKVLDLKNYIDKQLLPIVNKQ<br>SCSISNIETVIEFQQKNNRLLEITREFSVNAGVTTPVSTYMLTNSELLSLINDMPITNDQKKLMSNNVQI<br>VRQQSYSIMSIIKEEVLAYVVQLPLYGVIDTPCWKLHTSPLCTTNTKEGSNICLTRTDRGWYCDNAGSVS<br>FFPQAETCKVQSNRVFCDTMNSLTLPSEVNLCNVDIFNPKYDCKIMTSKTDVSSSVITSLGAIVSCYGKT<br>KCTASNKNRGIIKTFSNGCDYVSNKGVDTVSVGNTLYYVNKQEGKSLYVKGEPIINFYDPLVFPSDEFDA<br>SISQVNEKINQSLAFIRKSDELLHNVNVGKSTTNIMITTIIIVIIVILLLLIAVGLFLYCKARSTPVTLS<br>KDQLSGINNIAFSN
>AGT75346.1<br>MELPILKTNAITTILAAVTLCFASSQNITEEFYQSTCSAVSKGYLSALRTGWYTSVITIELSNIKENKCN<br>GTDAKVKLIKQELDKYKNAVTELQLLMQSTPAANSRARRELPRFMNYTLNNTKNTNVTLSKKRKRRFLGF<br>LLGVGSAIASGIAVSKVLHLEGEVNKIKSALLSTNKAVVSLSNGVSVLTSKVLDLKNYIDKQLLPIVNKQ<br>SCSISNIETVIEFQQKNNRLLEITREFSVNAGVTTPVSTYMLTNSELLSLINDMPITNDQKKLMSSNVQI<br>VRQQSYSIMSIIKEEVLAYVVQLPLYGVIDTPCWKLHTSPLCTTNTKEGSNICLTRTDRGWYCDNAGSVS<br>FFPQAETCKVQSNRVFCDTMNSLTLPSEVNLCNIDIFNPKYDCKIMTSKTDVSSSVITSLGAIVSCYGKT<br>KCTASNKNRGIIKTFSNGCDYVSNKGVDTVSVGNTLYYVNKQEGKSLYVKGEPIINFYDPLVFPSDEFDA<br>SISQVNEKINQSLAFIRKSDELLHNVNAGKSTTNIMITTIIIVIIVILLALIAVGLLLYCKARSTPVTLS<br>KDQLSGINNIAFSN
>AGT75379.1<br>MDLPILKTNAITAILAAVSLCFASSQNITEEFYQSTCSAVSKGYLSALRTGWYTSVITIELSNIKENKCN<br>GTDAKVKLIKQELDKYKNAVTELQLLMQSTPAANNRARRELPRFMNYTLNNTKNNNVTLSKKRKRRFLGF<br>LLGVGSAIASGIAVSKVLHLEGEVNKIKSALLSTNKAVVSLSNGVSVLTSKVLDLKNYIDKQLLPIVNKQ<br>SCSISNIETVIEFQQKNNRLLEITREFSVNAGVTTPVSTYMLTNSELLSLINDMPITNDQKKLMSNNVQI<br>VRQQSYSIMSIIKEEVLAYVVQLPLYGVIDTPCWKLHTSPLCTTNTKEGSNICLTRTDRGWYCDNAGSVS<br>FFPQAETCKVQSNRVFCDTMNSLTLPSEVNLCNIDIFNPKYDCKIMTSKTDVSSSVITSLGAIVSCYGKT<br>KCTASNKNRGIIKTFSNGCDYVSNKGVDTVSVGNTLYYVNKQEGKSLYVKGEPIINFYDPLVFPSDEFDA<br>SISQVNEKINQSLAFIRKSDELLHNVNVGKSTTNIMITTIIIVIIVILLLLIAVGLFLYCKARSTPVTLS<br>KDQLSGINNIAFSN
>AGT75390.1<br>MELPILKTNAITTILAAVTLCFASSQNITEEFYQSTCSAVSKGYLSALRTGWYTSVITIELSNIKENKCN<br>GTDAKVKLIKQELDKYKNAVTELQLLMQSTPAANSRARRELPRFMNYTLNNTKNTNVTLSKKRKRRFLGF<br>LLGVGSAIASGIAVSKVLHLEGEVNKIKSALLSTNKAVVSLSNGVSVLTSKVLDLKNYIDKQLLPIVNKQ<br>SCSISNIETVIEFQQKNNRLLEITREFSVNAGVTTPVSTYMLTNSELLSLINDMPITNDQKKLMSSNVQI<br>VRQQSYSIMSIIKEEVLAYVVQLPLYGVIDTPCWKLHTSPLCTTNTKEGSNICLTRTDRGWYCDNAGSVS<br>FFPQAETCKVQSNRVFCDTMNSLTLPSEVNLCNIDIFNPKYDCKIMTSKTDVSSSVITSLGAIVSCYGKT<br>KCTASNKNRGIIKTFSNGCDYVSNKGVDTVSVGNTLYYVNKQEGKSLYVKGEPIINFYDPLVFPSDEFDA<br>SISQVNEKINQSLAFIRKSDELLHNVNAGKSTTNIMITTIIIVIIVILLALIAVGLLLYCKARSTPVTLS<br>KDQLSGINNIAFSN

>AHA83621.1]
MDLPILKTNAITAILAAVSLCFASSQNITEEFYQSTCSAVSKGYLSALRTGWYTSVITIELSNIKENKCN
GTDAKVKLIKQELDKYKNAVTELQLLMQSTPAANNRARRELPRFMNYTLNNTKNNNVTLSKKRKRRFLGF
LLGVGSAIASGIAVSKVLHLEGEVNKIKSALLSTNKAVVSLSNGVSVLTSKVLDLKNYIDKQLLPIVNKQ
SCSISNIETVIEFQQKNNRLLEITREFSVNAGVTTPVSTYMLTNSELLSLINDMPITNDQKKLMSNNVQI
VRQQSYSIMSIIKEEVLAYVVQLPLYGVIDTPCWKLHTSPLCTTNTKEGSNICLTRTDRGWYCDNAGSVS
FFPQAETCKVQSNRVFCDTMNSLTLPSEVNLCNIDIFNPKYDCKIMTSKTDVSSSVITSLGAIVSCYGKT
KCTASNKNRGIIKTFSNGCDYVSNKGVDTVSVGNTLYYVNKQEGKSLYVKGEPIINFYDPLVFPSDEFDA
SISQVNEKINQSLAFIRKSDELLHNVNVGKSTTNIMITTIIIVIIVILLLLIAVGLFLYCKARSTPVTLS
KDQLSGINNIAFSN

>AHA83630.1
MELPILKTNAITTILAAVTLCFASSQNITEEFYQSTCSAVSKGYLSALRTGWYTSVITIELSNIKENKCN
GTDAKVKLIKQELDKYKNAVTELQLLMQSTPAANSRARRELPRFMNYTLNNTKNTNVTLSKKRKRRFLGF
LLGVGSAIASGIAVSKVLHLEGEVNKIKSALLSTNKAVVSLSNGVSVLTSKVLDLKNYIDKQLLPIVNKQ
SCSISNIETVIEFQQKNNRLLEITREFSVNAGVTTPVSTYMLTNSELLSLINDMPITNDQKKLMSSNVQI
VRQQSYSIMSIIKEEVLAYVVQLPLYGVIDTPCWKLHTSPLCTTNTKEGSNICLTRTDRGWYCDNAGSVS
FFPQAETCKVQSNRVFCDTMNSLTLPSEVNLCNIDIFNPKYDCKIMTSKTDVSSSVITSLGAIVSCYGKT
KCTASNKNRGIIKTFSNGCDYVSNKGVDTVSVGNTLYYVNKQEGKSLYVKGEPIINFYDPLVFPSDEFDA
SISQVNEKINQSLAFIRKSDELLHNVNVGKSTTNIMITTIIIVIIVILLALIAVGLLLYCRARSTPVTLS
KDQLSGINNIAFSN

>AHA83661.1
MELPILKTNAITTILAAVTLCFASSQNITEEFYQSTCSAVSKGYLSALRTGWYTSVITIELSNIKENKCN
GTDAKVKLIKQELDKYKNAVTELQLLMQSTPAANSRARRELPRFMNYTLNNTKNTNVTLSKKRKRRFLGF
LLGVGSAIASGIAVSKVLHLEGEVNKIKSALLSTNKAVVSLSNGVSVLTSKVLDLKNYIDKQLLPIVNKQ
SCSISNIETVIEFQQKNNRLLEITREFSVNAGVTTPVSTYMLTNSELLSLINDMPITNDQKKLMSNNVQI
VRQQSYSIMSIIKEEVLAYVVQLPLYGVIDTPCWKLHTSPLCTTNTKEGSNICLTRTDRGWYCDNAGSVS
FFPQADTCKVQSNRVFCDTMNSLTLPSEVNLCNIDIFNPKYDCKIMTSKTDVSSSVITSLGAIVSCYGKT
KCTASNKNRGIIKTFSNGCDYVSNKGVDTVSVGNTLYYVNKQEGKSLYVKGEPIINFYDPLVFPSDEFDA
SISQVNEKINQSLAFIRKSDELLHNVNAGKSTTNIMITTIIIVIIVILLSLIAVGLLLYCKARSTPVTLS
KDQLSGINNIAFSN

>AHA83683.1
MELPILKTNAITTILAAVTLCFASSQNITEEFYQSTCSAVSKGYLSALRTGWYTSVITIELSNIKENKCN
GTDAKVKLIKQELDKYKNAVTELQLLMQSTPAANNRARRELPRFMNYTLNNTKNNNVTLSKKRKRRFLGF
LLGVGSAIASGIAVSKVLHLEGEVNKIKSALLSTNKAVVSLSNGVSVLTSKVLDLKNYIDKQLLPIVNKQ
SCSISNIETVIEFQQKNNRLLEITREFSVNAGVTTPVSTYMLTNSELLSLINDMPITNDQKKLMSNNVQI
VRQQSYSIMSIIKEEVLAYVVQLPLYGVIDTPCWKLHTSPLCTTNTKEGSNICLTRTDRGWYCDNAGSVS
FFPQAETCKVQSNRVFCDTMNSLTLPSEVNLCNIDIFNPKYDCKIMTSKTDVSSSVITSLGAIVSCYGKT
KCTASNKNRGIIKTFSNGCDYVSNKGVDTVSVGNTLYYVNKQEGKSLYVKGEPIINFYDPLVFPSDEFDA
SISQVNEKINQSLAFIRKSDELLHNVNVGKSTTNIMITTIIIVIIVILLLLIAVGLFLYCKARSTPVTLS
KDQLSGINNIAFSN

>AHA83694.1
MELPILKTNAITTILAAVTLCFASSQNITEEFYQSTCSAVSKGYLSALRTGWYTSVITIELSNIKENKCN
GTDAKVKLIKQELDKYKNAVTELQLLMQSTPAANNRARRELPRFMNYTLNNTKNNNVTLSKKRKRRFLGF
LLGVGSAIASGIAVSKVLHLEGEVNKIKSALLSTNKAVVSLSNGVSVLTSKVLDLKNYIDKQLLPIVNKQ
SCSISNIETVIEFQQKNNRLLEITREFSVNAGVTTPVSTYMLTNSELLSLINDMPITNDQKKLMSNNVQI
VRQQSYSIMSIIKEEVLAYVVQLPLYGVIDTPCWKLHTSPLCTTNTKEGSNICLTRTDRGWYCDNAGSVS
FFPQAETCKVQSNRVFCDTMNSLTLPSDVNLCNIDIFNPKYDCKIMTSKTDVSSSVITSLGAIVSCYGKT
KCTASNKNRGIIKTFSNGCDYVSNKGVDTVSVGNTLYYVNKQEGKSLYVKGEPIINFYDPLVFPSDEFDA
SISQVNEKINQSLAFIRKSDELLHNVNVGKSTTNIMITTIIIVIIVILLLLIAVGLFLYCKARSTPVTLS
KDQLSGINNIAFSN

>AHA83716.1
MDLPILKTNAITAILAAVSLCFASSQNITEEFYQSTCSAVSKGYLSALRTGWYTSVITIELSNIKENKCN
GTDAKVKLIKQELDKYKNAVTELQLLMQSTPAANNRARRELPRFMNYTLNNTKNNNVTLSKKRKRRFLGF
LLGVGSAIASGIAVSKVLHLEGEVNKIKSALLSTNKAVVSLSNGVSVLTSKVLDLKNYIDKQLLPIVNKQ
SCSISNIETVIEFQQKNNRLLEITREFSVNAGVTTPVSTYMLTNSELLSLINDMPITNDQKKLMSNNVQI
VRQQSYSIMSIIKEEVLAYVVQLPLYGVIDTPCWKLHTSPLCTTNTKEGSNICLTRTDRGWYCDNAGSVS
FFPQAETCKVQSNRVFCDTMNSLTLPSEVNLCNIDIFNPKYDCKIMTSKTDVSSSVITSLGAIVSCYGKT
KCTASNKNRGIIKTFSNGCDYVSNKGVDTVSVGNTLYYVNKQEGKSLYVKGEPIINFYDPLVFPSDEFDA
SISQVNEKINQSLAFIRKSDELLHNVNVGKSTTNIMITTIIIVIIVILLLLIAVGLFLYCKARSTPVTLS
KDQLSGINNIAFSN

>AHA83727.1
MDLPILKTNAITAILAAVSLCFASSQNITEEFYQSTCSAVSKGYLSALRTGWYTSVITIELSNIKENKCN
GTDAKVKLIKQELDKYKNAVTELQLLMQSTPAANNRARRELPRFMNYTLNNTKNNNVTLSKKRKRRFLGF
LLGVGSAIASGIAVSKVLHLEGEVNKIKSALLSTNKAVVSLSNGVSVLTSKVLDLKNYIDKQLLPIVNKQ
SCSISNIETVIEFQQKNNRLLEITREFSVNAGVTTPVSTYMLTNSELLSLINDMPITNDQKKLMSNNVQI
VRQQSYSIMSIIKEEVLAYVVQLPLYGVIDTPCWKLHTSPLCTTNTKEGSNICLTRTDRGWYCDNAGSVS
FFPQAETCKVQSNRVFCDTMNSLTLPSEVNLCNIDIFNPKYDCKIMTSKTDVSSSVITSLGAIVSCYGKT
KCTASNKNRGIIKTFSNGCDYVSNKGVDTVSVGNTLYYVNKQEGKSLYVKGEPIINFYDPLVFPSDEFDA
SISQVNEKINQSLAFIRKSDELLHNVNVGKSTTNIMITTIIIVIIVILLLLIAVGLFLYCKARSTPVTLS
KDQLSGINNIAFSN

>AHA83738.1
MDLPILKTNAITAILAAVSLCFASSQNITEEFYQSTCSAVSKGYLSALRTGWYTSVITIELSNIKENKCN
GTDAKVKLIKQELDKYKNAVTELQLLMQSTPAANNRARRELPRFMNYTLNNTKNNNVTLSKKRKRRFLGF
LLGVGSAIASGIAVSKVLHLEGEVNKIKSALLSTNKAVVSLSNGVSVLTSKVLDLKNYIDKQLLPIVNKQ
SCSISNIETVIEFQQKNNRLLEITREFSVNAGVTTPVSTYMLTNSELLSLINDMPITNDQKKLMSNNVQI
VRQQSYSIMSIIKEEVLAYVVQLPLYGVIDTPCWKLHTSPLCTTNTKEGSNICLTRTDRGWYCDNAGSVS
FFPQAETCKVQSNRVFCDTMNSLTLPSEVNLCNIDIFNPKYDCKIMTSKTDVSSSVITSLGAIVSCYGKT
KCTASNKNRGIIKTFSNGCDYVSNKGVDTVSVGNTLYYVNKQEGKSLYVKGEPIINFYDPLVFPSDEFDA
SISQVNEKINQSLAFIRKSDELLHNVNVGKSTTNIMITTIIIVIIVILLLLIAVGLFLYCKARSTPVTLS
KDQLSGINNIAFSN

>AHA83760.1
MELPIINTNAITTILAAVTLCFASSQNITEEFYQSTCSAVSKGYLSALRTGWYTSVITIELSNIKENKCN
GTDAKVKLIKQELDKYKNAVTELQLLMQSTPAANSRARRELPRFMNYTLNNTKNTNVTLSKKRKRRFLGF
LLGVGSAIASGIAVSKVLHLEGEVNKIKSALLSTNKAVVSLSNGVSVLTSKVLDLKNYMDKQLLPIVNKQ
SCSISNIETVIEFQQKNNRLLEITREFSVNAGVTTPVSTYMLTNNELLSLINDMPITNDQKKLMSNNVQI
VRQQSYSIMSIIKEEVLAYVVQLPLYGVIDTPCWKLHTSPLCTTNTKEGSNICLTRTDRGWYCDNAGSVS
FFPQAETCKVQSNRVFCDTMNSLTLPSEVNLCNIDIFNPKYDCKIMTSKTDVSSSVITSLGAIVSCYGKT
KCTASNKNRGIIKTFSNGCDYVSNKGVDTVSVGNTLYYVNKQEGKSLYVKGEPIINFYDPLVFPSDEFDA
SISQVNEKINQSLAFIRKSDELLHNVNAGKSTTNIMITTIIIVIIVILLSLIAVGLLLYCKARSTPVTLS
KDQLSGINNIAFSN

>AHA83771.1
MELPILKTNAITTILAAVTLCFASSQNITEEFYQSTCSAVSKGYLSALRTGWYTSVITIELSNIKENKCN
GTDAKVKLIKQELDKYKNAVTELQLLMQSTPAANSRARRELPRFMNYTLNNTKNTNVTLSKKRKRRFLGF
LLGVGSAIASGIAVSKVLHLEGEVNKIKSALLSTNKAVVSLSNGVSVLTSKVLDLKNYIDKQLLPIVNKQ
SCSISNIETVIEFQQKNNRLLEITREFSVNAGVTTPVSTYMLTNSELLSLINDMPITNDQKKLMSNNVQI
VRQQSYSIMSIIKEEVLAYVVQLPLYGVIDTPCWKLHTSPLCTTNTKEGSNICLTRTDRGWYCDNAGSVS
FFPQAETCKVQSNRVFCDTMNSLTLPSEVNLCNIDIFNPKYDCKIMTSKTDVSSSVITSLGAIVSCYGKT
KCTASNKNRGIIKTFSNGCDYVSNKGVDTVSVGNTLYYVNKQEGKSLYVKGEPIINFYDPLVFPSDEFDA
SISQVNEKINQSLAFIRKSDELLHNVNAGKSTTNIMITTIIIVIIVILLSLIAVGLLLYCKARSTPVTLS
KDQLSGINNIAFSN

>AHA83782.1<br>MDLPILKTNAITAILAAVSLCFASSQNITEEFYQSTCSAVSKGYLSALRTGWYTSVITIELSNIKENKCN<br>GTDAKVKLIKQELDKYKNAVTELQLLMQSTPAANNRARRELPRFMNYTLNNTKNNNITLSKKRKRRFLGF<br>LLGVGSAIASGIAVSKVLHLEGEVNKIKSALLSTNKAVVSLSNGVSVLTSKVLDLKNYIDKQLLPIVNKQ<br>SCSISNIETVIEFQQKNNRLLEITREFSVNAGVTTPVSTYMLTNSELLSLINDMPITNDQKKLMSNNVQI<br>VRQQSYSIMSIIKEEVLAYVVQLPLYGVIDTPCWKLHTSPLCTTNTKEGSNICLTRTDRGWYCDNAGSVS<br>FFPQAETCKVQSNRVFCDTMNSLTLPSEVNLCNIDIFNPKYDCKIMTSKTDVSSSVITSLGAIVSCYGKT<br>KCTASNKNRGIIKTFSNGCDYVSNKGVDTVSVGNTLYYVNKQEGKSLYVKGEPIINFYDPLVFPSDEFDA<br>SISQVNEKINQSLAFIRKSDELLHNVNVGKSTTNIMITTIIIVIIVILLLLIAVGLFLYCKARSTPVTLS<br>KDQLSGINNIAFSN
>AHA83793.1<br>MELPILKTNAITTILAAVTLCFASSQNITEEFYQSTCSAVSKGYLSALRTGWYTSVITIELSNIKENKCN<br>GTDAKVKLIKQELDKYKNAVTELQLLMQSTPAANSRARRELPRFMNYTLNNTKNTNVTLSKKRKRRFLGF<br>LLGVGSAIASGIAVSKVLHLEGEVNKIKSALLSTNKAVVSLSNGVSVLTSKVLDLKNYIDKQLLPIVNKQ<br>SCSISNIETVIEFQQKNNRLLEITREFSVNAGVTTPVSTYMLTNSELLSLINDMPITNDQKKLMSSNVQI<br>VRQQSYSIMSIIKEEVLAYVVQLPLYGVIDTPCWKLHTSPLCTTNTKEGSNICLTRTDRGWYCDNAGSVS<br>FFPQAETCKVQSNRVFCDTMNSLTLPSEVNLCNIDIFNPKYDCKIMTSKTDVSSSVITSLGAIVSCYGKT<br>KCTASNKNRGIIKTFSNGCDYVSNKGVDTVSVGNTLYYVNKQEGKSLYVKGEPIINFYDPLVFPSDEFDA<br>SISQVNEKINQSLAFIRKSDELLHNVNAGKSTTNIMITTIIIVIIVILLALIAVGLLLYCKARSTPVTLS<br>KDQLSGINNIAFSN
>AHA83826.1<br>MDLPILKTNAITAILAAVSLCFASSQNITEEFYQSTCSAVSKGYLSALRTGWYTSVITIELSNIKENKCN<br>GTDAKVKLIKQELDKYKNAVTELQLLMQSTPAANNRARRELPRFMNYTLNNTKNNNVTLSKKRKRRFLGF<br>LLGVGSAIASGIAVSKVLHLEGEVNKIKSALLSTNKAVVSLSNGVSVLTSKVLDLKNYIDKQLLPIVNKQ<br>SCSISNIETVIEFQQKNNRLLEITREFSVNAGVTTPVSTYMLTNSELLSLINDMPITNDQKKLMSNNVQI<br>VRQQSYSIMSIIKEEVLAYVVQLPLYGVIDTPCWKLHTSPLCTTNTKEGSNICLTRTDRGWYCDNAGSVS<br>FFPQAETCKVQSNRVFCDTMNSLTLPSEVNLCNIDIFNPKYDCKIMTSKTDVSSSVITSLGAIVSCYGKT<br>KCTASNKNRGIIKTFSNGCDYVSNKGVDTVSVGNTLYYVNKQEGKSLYVKGEPIINFYDPLVFPSDEFDA<br>SISQVNEKINQSLAFIRKSNELLHNVNVGKSTTNIMITTIIIVIIVILLLLIAVGLFLYCKARSTPVTLS<br>KDQLSGINNIAFSN
>AHA83837.1<br>MDLPILKTNAITAILAAVSLCFASSQNITEEFYQSTCSAVSKGYLSALRTGWYTSVITIELSNIKENKCN<br>GTDAKVKLIKQELDKYKNAVTELQLLMQSTPAANNRARRELPRFMNYTLNNTKNNNVTLSKKRKRRFLGF<br>LLGVGSAIASGIAVSKVLHLEGEVNKIKSALLSTNKAVVSLSNGVSVLTSKVLDLKNYIDKQLLPIVNKQ<br>SCSISNIETVIEFQQKNNRLLEITREFSVNAGVTTPVSTYMLTNSELLSLINDMPITNDQKKLMSNNVQI<br>VRQQSYSIMSIIKEEVLAYVVQLPLYGVIDTPCWKLHTSPLCTTNTKEGSNICLTRTDRGWYCDNAGSVS<br>FFPQAETCKVQSNRVFCDTMNSLTLPNEVNLCNIDIFNPKYDCKIMTSKTDVSSSVITSLGAIVSCYGKT<br>KCTASNKNRGIIKTFSNGCDYVSNKGVDTVSVGNTLYYVNKQEGKSLYVKGEPIINFYDPLVFPSDEFDA<br>SISQVNEKINQSLAFIRKSDELLHNVNVGKSTTNIMITTIIIVIIVILLLLIAVGLFLYCKARSTPVTLS<br>KDQLSGINNIAFSN
>AHA83848.1<br>MELPILKTNAITTILAAVTLCFASSQNITEEFYQSTCSAVSKGYLSALRTGWYTSVITIELSNIKENKCN<br>GTDAKVKLIKQELDKYKNAVTELQLLMQSTPAANSRARRELPRFMNYTLNNTKNTNVTLSKKRKRRFLGF<br>LLGVGSAIASGIAVSKVLHLEGEVNKIKSALLSTNKAVVSLSNGVSVLTSKVLDLKNYIDKQLLPIVNKQ<br>SCSISNIETVIEFQQKNNRLLEITREFSVNAGVTTPVSTYMLTNSELLSLINDMPITNDQKKLMSNNVQI<br>VRQQSYSIMSIIKEEVLAYVVQLPLYGVIDTPCWKLHTSPLCTTNTKEGSNICLTRTDRGWYCDNAGSVS<br>FFPQAETCKVQSNRVFCDTMNSLTLPSEVNLCNIDIFNPKYDCKIMTSKTDVSSSVITSLGAIVSCYGKT<br>KCTASNKNRGIIKTFSNGCDYVSNKGVDTVSVGNTLYYVNKQEGKSLYVKGEPIINFYDPLVFPSDEFDA<br>SISQVNEKINQSLAFIRKSDELLHNVNAGKSTTNIMITTIIIVIIVILLSLIAVGLLLYCKARSTPVTLS<br>KDQLSGINNIAFSN

>AHA83869.1
MELPILKTNAITTILAAVTLCFASSQNITEEFYQSTCSAVSKGYLSALRTGWYTSVITIELSNIKENKCN
GTDAKVKLIKQELDKYKNAVTELQLLMQSTPAANSRARRELPRFMNYTLNNTKNTNVTLSKKRKRRFLGF
LLGVGSAIASGIAVSKVLHLEGEVNKIKSALLSTNKAVVSLSNGVSVLTSKVLDLKNYIDKQLLPIVNKQ
SCSISNIETVIEFQQKNNRLLEITREFSVNAGVTTPVSTYMLTNSELLSLINDMPITNDQKKLMSNNVQI
VRQQSYSIMSIIKEEVLAYVVQLPLYGVIDTPCWKLHTSPLCTTNTKEGSNICLTRTDRGWYCDNAGSVS
FFPQADTCKVQSNRVFCDTMNSLTLPSEVNLCNIDIFNPKYDCKIMTSKTDVSSSVITSLGAIVSCYGKT
KCTASNKNRGIIKTFSNGCDYVSNKGVDTVSVGNTLYYVNKQEGKSLYVKGEPIINFYDPLVFPSDEFDA
SISQVNEKINQSLAFIRKSDELLHNVNAGKSTTNIMITTIIIVIIVILLSLIAVGLLLYCKARSTPVTLS
KDQLSGINNIAFSN

>AHA83880.1
MDLPILKINAITAILAAVSLCFASSQNITEEFYQSTCSAVSKGYLSALRTGWYTSVITIELSNIKENKCN
GTDAKVKLIKQELDKYKNAVTELQLLMQSTPAANNRARRELPRFMNYTLNNTKNNNVTLSKKRKRRFLGF
LLGVGSAIASGIAVSKVLHLEGEVNKIKSALLSTNKAVVSLSNGVSVLTSKVLDLKNYIDKQLLPIVNKQ
SCSISNIETVIEFQQKNNRLLEITREFSVNAGVTTPVSTYMLTNSELLSLINDMPITNDQKKLMSNNVQI
VRQQSYSIMSIIKEEVLAYVVQLPLYGVIDTPCWKLHTSPLCTTNTKEGSNICLTRTDRGWYCDNAGSVS
FFPQAETCKVQSNRVFCDTMNSLTLPSEVNLCNIDIFNPKYDCKIMTSKTDVSSSVITSLGAIVSCYGKT
KCTASNKNRGIIKTFSNGCDYVSNKGVDTVSVGNTLYYVNKQEGKSLYVKGEPIINFYDPLVFPSDEFDA
SISQVNEKINQSLAFIRKSDELLHNVNVGKSTTNIMITTIIIVIIVILLLLIAVGLFLYCKARSTPVTLS
KDQLSGINNIAFSN

>AHA83935.1
MDLPILKTNAITAILAAVSLCFASSQNITEEFYQSTCSAVSKGYLSALRTGWYTSVITIELSNIKENKCN
GTDAKVKLIKQELDKYKNAVTELQLLMQSTPAANNRARRELPRFMNYTLNNTKNNNVTLSKKRKRRFLGF
LLGVGSAIASGIAVSKVLHLEGEVNKIKSALLSTNKAVVSLSNGVSVLTSKVLDLKNYIDKQLLPIVNKQ
SCSISNIETVIEFQQKNNRLLEITREFSVNAGVTTPVSTYMLTNSELLSLINDMPITNDQKKLMSNNVQI
VRQQSYSIMSIIKEEVLAYVVQLPLYGVIDTPCWKLHTSPLCTTNTKEGSNICLTRTDRGWYCDNAGSVS
FFPQAETCKVQSNRVFCDTMNSLTLPSEVNLCNIDIFNPKYDCKIMTSKTDVSSSVITSLGAIVSCYGKT
KCTASNKNRGIIKTFSNGCDYVSNKGVDTVSVGNTLYYVNKQEGKSLYVKGEPIINFYDPLVFPSDEFDA
SISQVNEKINQSLAFIRKSDELLHNVNVGKSTTNIMITTIIIVIIVILLLLIAVGLFLYCKARSTPVTLS
KDQLSGINNIAFSN

>AHA83946.1
MELPILKTNAITTIFAAVTLCFASSQNITEEFYQSTCSAVSKGYLSALRTGWYTSVITIELSNIKENKCN
GTDAKVKLIKQELDKYKNAVTELQLLMQSTPAANNRARRELPRFMNYTLNNTKNNNVTLSKKRKRRFLGF
LLGVGSAIASGIAVSKVLHLEGEVNKIKSALLSTNKAVVSLSNGVSVLTSKVLDLKNYIDKQLLPIVNKQ
SCSISNIETVIEFQQKNNRLLEITREFSVNAGVTTPVSTYMLTNSELLSLINDMPITNDQKKLMSNNVQI
VRQQSYSIMSIIKEEVLAYVVQLPLYGVIDTPCWKLHTSPLCTTNTKEGSNICLTRTDRGWYCDNAGSVS
FFPQAETCKVQSNRVFCDTMNSLTLPSEVNLCNIDIFNPKYDCKIMTSKTDVSSSVITSLGAIVSCYGKT
KCTASNKNRGIIKTFSNGCDYVSNKGVDTVSVGNTLYYVNKQEGKSLYVKGEPIINFYDPLVFPSDEFDA
SISQVNEKINQSLAFIRKSDELLHNVNVGKSTTNIMITTIIIVIIVILLLLIAVGLFLYCKARSTPVTLS
KDQLSGINNIAFSN

>AHA83957.1
MELPILKTNAITTILAVVTLCFASSQNITEEFYQSTCSAVSKGYLSALRTGWYTSVITIELSNIKENKCN
GTDAKVKLIKQELDKYKNAVTELQLLMQSTPASNNRARRELPRFMNYTLNNTKNTNVTLSKKRKRRFLGF
LLGVGSAIASGIAVSKVLHLEGEVNKIKSALLSTNKAVVSLSNGVSVLTSKVLDLKNYIDKQLLPIVNKQ
SCSISNIETVIEFQQKNNRLLEITREFSVNAGVTTPVSTYMLTNSELLSLINDMPITNDQKKLMSNNVQI
VRQQSYSIMSIIKEEVLAYVVQLPLYGVIDTPCWKLHTSPLCTTNTKEGSNICLTRTDRGWYCDNAGSVS
FFPQAETCKVQSNRVFCDTMNSLTLPSEVNLCNIDIFNPKYDCKIMTSKTDVSSSVITSLGAIVSCYGKT
KCTASNKNRGIIKTFSNGCDYVSNKGVDTVSVGNTLYYVNKQEGKSLYVKGEPIINFYDPLVFPSDEFDA
SISQVNEKINQSLAFIRKSDELLHNVNAGKSTTNIMITTIIIVIVVILLSLIAVGLLLYCKARSTPVTLS
KDQLSGINNIAFSS

>AHA83968.1
MELPILKTNAITTILAAVTLCFASSQNITEEFYQSTCSAVSKGYLSALRTGWYTSVITIELSNIKENKCN
GTDAKVKLIKQELDKYKNAVTELQLLMQSTPAANSRARRELPRFMNYTLNNTKNTNVTLSKKRKRRFLGF
LLGVGSAIASGIAVSKVLHLEGEVNKIKSALLSTNKAVVSLSNGVSVLTSKVLDLKNYIDKQLLPIVNKQ
SCSISNIETVIEFQQKNNRLLEITREFSVNAGVTTPVSTYMLTNSELLSLINDMPITNDQKKLMSSNVQI
VRQQSYSIMSIIKEEVLAYVVQLPLYGVIDTPCWKLHTSPLCTTNTKEGSNICLTRTDRGWYCDNAGSVS
FFPQAETCKVQSNRVFCDTMNSLTLPSEVNLCNIDIFNPKYDCKIMTSKTDVSSSVITSLGAIVSCYGKT
KCTASNKNRGIIKTFSNGCDYVSNKGVDTVSVGNTLYYVNKQEGKSLYVKGEPIINFYDPLVFPSDEFDA
SISQVNEKINQSLAFIRKSDELLHNVNAGKSTTNIMITTIIIVIIVILLALIAVGLLLYCKARSTPVTLS
KDQLSGINNIAFSN

>AHA83979.1
MDLPILKTNAITAILAAVSLCFASSQNITEEFYQSTCSAVSKGYLSALRTGWYTSVITIELSNIKENKCN
GTDAKVKLIKQELDKYKNAVTELQLLMQSTPAANNRARRELPRFMNYTLNNTKNNNVTLSKKRKRRFLGF
LLGVGSAIASGIAVSKVLHLEGEVNKIKSALLSTNKAVVSLSNGVSVLTSKVLDLKNYIDKQLLPIVNKQ
SCSISNIETVIEFQQKNNRLLEITREFSVNAGVTTPVSTYMLTNSELLSLINDMPITNDQKKLMSNNVQI
VRQQSYSIMSIIKEEVLAYVVQLPLYGVIDTPCWKLHTSPLCTTNTKEGSNICLTRTDRGWYCDNAGSVS
FFPQAETCKVQSNRVFCDTMNSLTLPSEVNLCNIDIFNPKYDCKIMTSKTDVSSSVITSLGAIVSCYGKT
KCTASNKNRGIIKTFSNGCDYVSNKGVDTVSVGNTLYYVNKQEGKSLYVKGEPIINFYDPLVFPSDEFDA
SISQVNEKINQSLAFIRKSDELLHNVNVGKSTTNIMITTIIIVIIVILLLLIAVGLFLYCKARSTPVTLS
KDQLSGINNIAFSN

>AHA84001.1
MDLPILKTNAITAILAAVSLCFASSQNITEEFYQSTCSAVSKGYLSALRTGWYTSVITIELSNIKENKCN
GTDAKVKLIKQELDKYKNAVTELQLLMQSTPAANNRARRELPRFMNYTLNNTKNNNVTLSKKRKRRFLGF
LLGVGSAIASGIAVSKVLHLEGEVNKIKSALLSTNKAVVSLSNGVSVLTSKVLDLKNYIDKQLLPIVNKQ
SCSISNIETVIEFQQKNNRLLEITREFSVNAGVTTPVSTYMLTNSELLSLINDMPITNDQKKLMSNNVQI
VRQQSYSIMSIIKEEVLAYVVQLPLYGVIDTPCWKLHTSPLCTTNTKEGSNICLTRTDRGWYCDNAGSVS
FFPQAETCKVQSNRVFCDTMNSLTLPSEVNLCNIDIFNPKYDCKIMTSKTDVSSSVITSLGAIVSCYGKT
KCTASNKNRGIIKTFSNGCDYVSNKGVDTVSVGNTLYYVNKQEGKSLYVKGEPIINFYDPLVFPSDEFDA
SISQVNEKINQSLAFIRKSDELLHNVNVGKSTTNIMITTIIIVIIVILLLLIAVGLFLYCKARSTPVTLS
KDQLSGINNIAFSN

>AHA84023.1
MELPILKTNAITTIFATVTLCFASSQNITEEFYQSTCSAVSKGYLSALRTGWYTSVITIELSNIKENKCN
GTDAKVKLIKQELDKYKNAVTELQLLMQSTPAANNRARRELPRFMNYTLNNTKNNNVTLSKKRKRRFLGF
LLGVGSAIASGIAVSKVLHLEGEVNKIKSALLSTNKAVVSLSNGVSVLTSKVLDLKNYIDKQLLPIVNKQ
SCSISNIETVIEFQQKNNRLLEITREFSVNAGVTTPVSTYMLTNSELLSLINDMPITNDQKKLMSNNVQI
VRQQSYSIMSIIKEEVLAYVVQLPLYGVIDTPCWKLHTSPLCTTNTKEGSNICLTRTDRGWYCDNAGSVS
FFPQAETCKVQSNRVFCDTMNSLTLPSEVNLCNVDIFNPKYDCKIMTSKTDVSSSVITSLGAIVSCYGKT
KCTASNKNRGIIKTFSNGCDYVSNKGVDTVSVGNTLYYVNKQEGKSLYVKGEPIINFYDPLVFPSDEFDA
SISQVNEKINQSLAFIRKSDELLHNVNVGKSTTNIMITTIIIVIIVILLLLIAVGLFLYCKARSTPVTLS
KDQLSGINNIAFSN

>AHA84034.1
MELPILKTNAITTILAAVTLCFASSQNITEEFYQSTCSAVSKGYLSALRTGWYTSVITIELSNIKENKCN
GTDAKVKLIKQELDKYKNAVTELQLLMQSTPAANSRARRELPRFMNYTLNNTKNTNVTLSKKRKRRFLGF
LLGVGSAIASGIAVSKVLHLEGEVNKIKSALLSTNKAVVSLSNGVSVLTSKVLDLKNYIDKQLLPIVNKQ
SCSISNIETVIEFQQKNNRLLEITREFSVNAGVTTPVSTYMLTNSELLSLINDMPITNDQKKLMSSNVQI
VRQQSYSIMSIIKEEVLAYVVQLPLYGVIDTPCWKLHTSPLCTTNTKEGSNICLTRTDRGWYCDNAGSVS
FFPQAETCKVQSNRVFCDTMNSLTLPSEVDLCNIDIFNPKYDCKIMTSKTDVSSSVITSLGAIVSCYGKT
KCTASNKNRGIIKTFSNGCDYVSNKGVDTVSVGNTLYYVNKQEGKSLYVKGEPIINFYDPLVFPSDEFDA
SISQVNEKINQSLAFIRKSDELLHNVNAGKSTTNIMITTIIIVIIVILLALIAVGLLLYCKARSTPVTLS
KDQLSGINNIAFSN

>AHA84045.1
MELPILKTNAITTILAAVTLCFASSQNITEEFYQSTCSAVSKGYLSALRTGWYTSVITIELSNIKENKCN
GTDAKVKLIKQELDKYKNAVTELQLLMQSTPAANSRARRELPRFMNYTLNNTKNTNVTLSKKRKRRFLGF
LLGVGSAIASGIAVSKVLHLEGEVNKIKSALLSTNKAVVSLSNGVSVLTSKVLDLKNYIDKQLLPIVNKQ
SCSISNIETVIEFQQKNNRLLEITREFSVNAGVTTPVSTYMLTNSELLSLINDMPITNDQKKLMSSNVQI
VRQQSYSIMSIIKEEVLAYVVQLPLYGVIDTPCWKLHTSPLCTTNTKEGSNICLTRTDRGWYCDNAGSVS
FFPQAETCKVQSNRVFCDTMNSLTLPSEVNLCNIDIFNPKYDCKIMTSKTDVSSSVITSLGAIVSCYGKT
KCTASNKNRGIIKTFSNGCDYVSNKGVDTVSVGNTLYYVNKQEGKSLYVKGEPIINFYDPLVFPSDEFDA
SISQVNEKINQSLAFIRKSDELLHNVNAGKSTTNIMITTIIIVIIVILLALIAVGLLLYCKARSTPVTLS
KDQLSGINNIAFSN

>AGU13783.1
MELLIHRSSAIFLTLAINALYLTSSQNITEEFYQSTCSAVSRGYLSALRTGWYTSVITIELSNIKETKCN
GTDTKVKLIKQELDKYKNAVTELQLLMQNTPAANNRARREAPQYMNYTINTTKNLNVSISKKRKRRFLGF
LLGVGSAIASGIAVSKVLHLEGEVNKIKNALLSTNKAVVSLSNGVSVLTSKVLDLKNYINNQLLPIVNQQ
SCRISNIETVIEFQQKNSRLLEITREFSVNAGVTTPLSTYMLTNSELLSLINDMPITNDQKKLMSSNVQI
VRQQSYSIMSIIKEEVLAYVVQLPIYGVIDTPCWKLHTSPLCTTNIKEGSNICLTRTDRGWYCDNAGSVS
FFPQADTCKVQSNRVFCDTMNSLTLPSEVSLCNTDIFNSKYDCKIMTSKTDISSSVITSLGAIVSCYGKT
KCTASNKNRGIIKTFSNGCDYVSNKGVDTVSVGNTLYYVNKLEGKNLYVKGEPIINYYDPLVFPSDEFDA
SISQVNEKINQSLAFIRRSDELLHNVNTGKSTTNIMITAIIIVIIVVLLSLIAIGLLLYCKAKNTPVTLS
KDQLSGINNIAFSK

>AGT75357.1
MELLIHRSSAIFLTLAINALYLTSSQNITEEFYQSTCSAVSRGYLSALRTGWYTSVITIELSNIKETKCN
GTDTKVKLIKQELDKYKNAVTELQLLMQNTPAANNRARREAPQYMNYTINTTKNLNVSISKKRKRRFLGF
LLGVGSAIASGIAVSKVLHLEGEVNKIKNALLSTNKAVVSLSNGVSVLTSKVLDLKNYINNQLLPIVNQQ
SCRISNIETVIEFQQKNSRLLEITREFSVNAGVTIPLSTYMLTNSELLSLINDMPITNDQKKLMSSNVQI
VRQQSYSIMSIIKEEVLAYVVQLPIYGVIDTPCWKLHTSPLCTTNIKEGSNICLTRTDRGWYCDNAGSVS
FFPQADTCKVQSNRVFCDTMNSLTLPSEVSLCNTDIFNSKYDCKIMTSKTDISSSVITSLGAIVSCYGKT
KCTASNKNRGIIKTFSNGCDYVSNKGVDTVSVGNTLYYVNKLEGKNLYVKGEPIINYYDPLVFPSDEFDA
SISQVNEKINQSLAFIRRSDELLHNVNTGKSTTNIMITVIIIVIIVVLLSLIAIGLLLYCKAKNTPVTLS
KDQLSGINNIAFSK

>AGT75368.1
MELLIHRSSAIFLTLAINALYLTSSQNITEEFYQSTCSAVSRGYLSALRTGWYTSVITIELSNIKETKCN
GTDTKVKLIKQELDKYKNAVTELQLLMQNTPAANNRARREAPQYMNYTINTTKNLNVSISKKRKRRFLGF
LLGVGSAIASGIAVSKVLHLEGEVNKIKNALLSTNKAVVSLSNGVSVLTSKVLDLKNYINNQLLPIVNQQ
SCRISNIETVIEFQQKNSRLLEITREFSVNAGVTTPLSTYMLTNSELLSLINDMPITNDQKKLMSSNVQI
VRQQSYSIMSIIKEEVLAYVVQLPIYGVIDTPCWKLHTSPLCTTNIKEGSNICLTRTDRGWYCDNAGSVS
FFPQADTCKVQSNRVFCDTMNSLTLPSEVSLCNTDIFNSKYDCKIMTSKTDISSSVITSLGAIVSCYGKT
KCTASNKNRGIIKTFSNGCDYVSNKGVDTVSVGNTLYYVNKLEGKNLYVKGEPIINYYDPLVFPSDEFDA
SISQVNEKINQSLAFIRRSDELLHNVNTGKSTTNIMITVIIIVIIVVLLSLIAIGLLLYCKAKNTPVTLS
KDQLSGINNIAFSK

>AHA83641.1
MELLIHRSSAIFLTLAINALYLTSSQNITEEFYQSTCSAVSRGYLSALRTGWYTSVITIELSNIKETKCN
GTDTKVKLIKQELDKYKNAVTELQLLMQNTPAANNRARREAPQYMNYTINTTKNLNVSISKKRKRRFLGF
LLGVGSAIASGIAVSKVLHLEGEVNKIKNALLSTNKAVVSLSNGVSVLTSKVLDLKNYINNQLLPIVNQQ
SCRISNIETVIEFQQKNSRLLEITREFSVNAGVTTPLSTYMLTNSELLSLINDMPITNDQKKLMSSNVQI
VRQQSYSIMSIIKEEVLAYVVQLPIYGVIDTPCWKLHTSPLCTTNIKEGSNICLTRTDRGWYCDNAGSVS
FFPQADTCKVQSNRVFCDTMNSLTLPSEVSLCNTDIFNSKYDCKIMTSKTDISSSVITSLGAIVSCYGKT
KCTASNKNRGIIKTFSNGCDYVSNKGVDTVSVGNTLYYVNKLEGKNLYVKGEPIINYYDPLVFPSDEFDA
SISQVNEKINQSLAFIRRSDELLHNVNTGKSTTNIMITVIIIVIIVVLLSLIAIGLLLYCKAKNTPVTLS
KDQLSGINNIAFSK

>AHA83650.1
MELLIHRSSAIFLTLAINALYLTSSQNITEEFYQSTCSAVSRGYLSALRTGWYTSVITIELSNIKETKCN
GTDTKVKLIKQELDKYKNAVTELQLLMQNTPAANNRARREAPQYMNYTINTTKNLNVSISKKRKRRFLGF
LLGVGSAIASGIAVSKVLHLEGEVNKIKNALLSTNKAVVSLSNGVSVLTSKVLDLKNYINNQLLPIVNQQ
SCRISNIETVIEFQQKNSRLLEITREFSVNAGVTTPLSTYMLTNSELLSLINDMPITNDQKKLMSSNVQI
VRQQSYSIMSIIKEEVLAYVVQLPIYGVIDTPCWKLHTSPLCTTNIKEGSNICLTRTDRGWYCDNAGSVS
FFPQADTCKVQSNRVFCDTMNSLTLPSEVSLCNTDIFNSKYDCKIMTSKTDISSSVITSLGAIVSCYGKT
KCTASNKNRGIIKTFSNGCDYVSNKGVDTVSVGNTLYYVNKLEGKNLYVKGEPIINYYDPLVFPSDEFDA
SISQVNEKINQSLAFIRRSDELLHNVNTGKSTTNIMITVIIIVIIVVLLSLIAIGLLLYCKAKNTPVTLS
KDQLSGINNIAFSK

>AHA83672.1
MELLIHRSSAIFLTLAINALYLTSSQNITEEFYQSTCSAVSRGYLSALRTGWYTSVITIELSNIKETKCN
GTDTKVKLIKQELDKYKNAVTELQLLMQNTPAANNRARREAPQYMNYTINTTKNLNVSISKKRKRRFLGF
LLGVGSAIASGIAVSKVLHLEGEVNKIKNALLSTNKAVVSLSNGVSVLTSKVLDLKNYINNQLLPIVNQQ
SCRISNIETVIEFQQKNSRLLEITREFSVNAGVTTPLSTYMLTNSELLSLINDMPITNDQKKLMSSNVQI
VRQQSYSIMSIIKEEVLAYVVQLPIYGVIDTPCWKLHTSPLCTTNIKEGSNICLTRTDRGWYCDNAGSVS
FFPQADTCKVQSNRVFCDTMNSLTLPSEVSLCNTDIFNSKYDCKIMTSKTDISSSVITSLGAIVSCYGKT
KCTASNKNRGIIKTFSNGCDYVSNKGVDTVSVGNTLYYVNKLEGKNLYVKGEPIINYYDPLVFPSDEFDA
SISQVNEKINQSLAFIRRSDELLHNVNTGKSTTNIMITAIIIVIIVVLLSLIAIGLLLYCKAKNTPVTLS
KDQLSGINNIAFSK

>AHA83705.1
MELLIHRSSAIFLTFAINALYLTSSQNITEEFYQSTCSAVSRGYLSALRTGWYTSVITIELSNIKETKCN
GTDTKVKLIKQELDKYKNAVTELQLLMQNTPAANNRARREAPQYMNYTINTTKNLNVSISKKRKRRFLGF
LLGVGSAIASGIAVSKVLHLEGEVNKIKNALLSTNKAVVSLSNGVSVLTSKVLDLKNYINNQLLPIVNQQ
SCRISNIETVIEFQQKNSRLLEITREFSVNAGVTTPLSTYMLTNSELLSLINDMPITNDQKKLMSSNVQI
VRQQSYSIMSIIKEEVLAYVVQLPIYGVIDTPCWKLHTSPLCTTNIKEGSNICLTRTDRGWYCDNAGSVS
FFPQADTCKVQSNRVFCDTMNSLTLPSEVSLCNTDIFNSKYDCKIMTSKTDISSSVITSLGAIVSCYGKT
KCTASNKNRGIIKTFSNGCDYVSNKGVDTVSVGNTLYYVNKLEGKNLYVKGEPIINYYDPLVFPSDEFDA
SISQVNEKINQSLAFIRRSDELLHNVNTGKSTTNIMITAIIIVIIVVLLSLIAIGLLLYCNAKNTPVTLS
KDQLSGINNIAFSK

>AHA83749.1
MELLIHRSSAIFLTLAINALYLTSSQNITEEFYQSTCSAVSRGYFSALRTGWYTSVITIELSNIKETKCN
GTDTKVKLIKQELDKYKNAVTELQLLMQNTPAANNRARREAPQYMNYTINTTKNLNVSISKKRKRRFLGF
LLGVGSAIASGIAVSKVLHLEGEVNKIKNALLSTNKAVVSLSNGVSVLTSKVLDLKNYINNQLLPIVNQQ
SCRISNIETVIEFQQKNSRLLEITREFSVNAGVTTPLSTYMLTNSELLSLINDMPITNDQKKLMSSNAQI
VRQQSYSIMSIIKEEVLAYVVQLPIYGVIDTPCWKLHTSPLCTTNTKEGSNICLTRTDRGWYCDNAGSVS
FFPQADTCKVQSNRVFCDTMNSLTLPSEVSLCNTDIFNSKYDCKIMTSKTDISSSVITSLGAIVSCYGKT
KCTASNKNRGIIKTFSNGCDYVSNKGVDTVSVGNTLYYVNKLEGKNLYVKGEPIINYYDPLVFPSDEFDA
SISQVNEKINQSLAFIRRSDELLHNVNTGKSTTNIMITAIIIVIIVVLLSLIAIGLLLYCKAKNTPVTLS
KDQLSGINNIAFSK

>AHA83804.1
MELLIHRSSAIFLTLAINALYLTSSQNITEEFYQSTCSAVSRGYLSALRTGWYTSVITIELSNIKETKCN
GTDTKVKLIKQELDKYKNAVTELQLLMQNTPAVNNRARREAPQYMNYTINTTKNLNVSISKKRKRRFLGF
LLGVGSAIASGIAVSKVLHLEGEVNKIKNALLSTNKAVVSLSNGVSVLTSKVLDLKNYINNQLLPIVNQQ
SCRISNIETVIEFQQKNSRLLEITREFSVNAGVTTPLSTYMLTNSELLSLINDMPITNDQKKLMSSNVQI
VRQQSYSIMSIIKEEVLAYVVQLPIYGVIDTPCWKLHTSPLCTTNIKEGSNICLTRTDRGWYCDNAGSVS
FFPQADTCKVQSNRVFCDTMNSLTLPSEVSLCNTDIFNSKYDCKIMTSKTDISSSVITSLGAIVSCYGKT
KCTASNKNRGIIKTFSNGCDYVSNKGVDTVSVGNTLYYVNKLEGKNLYVKGEPIINYYDPLVFPSDEFDA
SISQVNEKINQSLAFIRRSDELLHNVNTGKSTTNIMITAIIIVIIVVLLSLIAIGLLLYCKAKNTPVTLS
KDQLSGINNIAFSK

>AHA83815.1
MELLIHRSSAIFLTLAINALYLTSSQNITEEFYQSTCSAVSRGYLSALRTGWYTSVITIELSNIKETKCN
GTDTKVKLIKQELDKYKNAVTELQLLMQNTPAVNNRARREAPQYMNYTINTTKNLNVSISKKRKRRFLGF
LLGVGSAIASGIAVSKVLHLEGEVNKIKNALLSTNKAVVSLSNGVSVLTSKVLDLKNYINNQLLPIVNQQ
SCRISNIETVIEFQQKNSRLLEITREFSVNAGVTTPLSTYMLTNSELLSLINDMPITNDQKKLMSSNVQI
VRQQSYSIMSIIKEEVLAYVVQLPIYGVIDTPCWKLHTSPLCTTNIKEGSNICLTRTDRGWYCDNAGSVS
FFPQADTCKVQSNRVFCDTMNSLTLPSEVSLCNTDIFNSKYDCKIMTSKTDISSSVITSLGAIVSCYGKT
KCTASNKNRGIIKTFSNGCDYVSNKGVDTVSVGNTLYYVNKLEGKNLYVKGEPIINYYDPLVFPSDEFDA
SISQVNEKINQSLAFIRRSDELLHNVNTGKSTTNIMITAIIIVIIVVLLSLIAIGLLLYCKAKNTPVTLS
KDQLSGINNIAFSK

>AHA83858.1
MELLIHRSSAIFLTLAINALYLTSSQNITEEFYQSTCSAVSRGYFSALRTGWYTSVITIELSNIKETKCN
GTDTKVKLIKQELDKYKNAVTELQLLMQNTPAANNRARREAPQYMNYTINTTKNLNVSISKKRKRRFLGF
LLGVGSAIASGIAVSKVLHLEGEVNKIKNALLSTNKAVVSLSNGVSVLTSKVLDLKNYINNQLLPIVNQQ
SCRISNIETVIEFQQKNSRLLEITREFSVNAGVTTPLSTYMLTNSELLSLINDMPITNDQKKLMSSNVQI
VRQQSYSIMSIIKEEVLAYVVQLPIYGVIDTPCWKLHTSPLCTTNIKEGSNICLTRTDRGWYCDNAGSVS
FFPQADTCKVQSNRVFCDTMNSLTLPSEVSLCNTDIFNSKYDCKIMTSKTDISSSVITSLGAIVSCYGKT
KCTASNKNRGIIKTFSNGCDYVSNKGVDTVSVGNTLYYVNKLEGKNLYVKGEPIINYYDPLVFPSDEFDA
SISQVNEKINQSLAFIRRSDELLHNVNTGKSTTNIMITAIIIVIIVVLLSLIAIGLLLYCKAKNTPVTLS
KDQLSGINNIAFSK

>AHA83891.1
MELLIHRSSAIFLTLAINALYLTSSQNITEEFYQSTCSAVSRGYLSALRTGWYTSVITIELSNIKETKCN
GTDTKVKLIKQELDKYKNAVTELQLLMQNTPAANNRARREAPQYMNYTINATKNLNVSISKKRKRRFLGF
LLGVGSAIASGIAVSKVLHLEGEVNKIKNALLSTNKAVVSLSNGVSVLTSKVLDLKNYINNQLLPIVNQQ
SCRISNIETVIEFQQKNSRLLEITREFSVNAGVTTPLSTYMLTNSELLSLINDMPITNDQKKLMSSNVQI
VRQQSYSIMSIIKEEVLAYVVQLPIYGVIDTPCWKLHTSPLCTTNIKEGSNICLTRTDRGWYCDNAGSVS
FFPQADTCKVQSNRVFCDTMNSLTLPSEVSLCNTDIFNSKYDCKIMTSKTDISSSVITSLGAIVSCYGKT
KCTASNKNRGIIKTFSNGCDYVSNKGVDTVSVGNTLYYVNKLEGKNLYVKGEPIINYYDPLVFPSDEFDA
SISQVNEKINQSLAFIRRSDELLHNVNTGKSTTNIMITAIIIVIIVVLLSLIAIGLLLYCKAKNTPVTLS
KDQLSGINNIAFSK

>AHA83902.1
MELLIHRSSAIFLTLAINALYLTSSQNITEEFYQSTCSAVSRGYISALRTGWYTSVITIELSNIKETKCN
GTDTKVKLIKQELDKYKNAVTELQLLMQNTPAANNRARREAPQYMNYTINTTKNLNVSISKKRKRRFLGF
LLGVGSAIASGIAVSKVLHLEGEVNKIKNALLSTNKAVVSLSNGVSVLTSKVLDLKNYINNQLLPIVNQQ
SCRISNIETVIEFQQKNSRLLEITREFSVNAGVTTPLSTYMLTNSELLSLINDMPITNDQKKLMSSNVQI
VRQQSYSIMSIIKEEVLAYVVQLPIYGVIDTPCWKLHTSPLCTTNIKEGSNICLTRTDRGWYCDNAGSVS
FFPQADTCKVQSNRVFCDTMNSLTLPSEVSLCNTDIFNSKYDCKIMTSKTDISSSVITSLGAIVSCYGKT
KCTASNKNRGIIKTFSNGCDYVSNKGVDTVSVGNTLYYVNKLEGKNLYVKGEPIINYYDPLMFPSDEFDA
SISQVNEKINQSLAFIRRSDELLHNVNTGKSTTNIMITAIIIVIIVVLLSLIAIGLLLYCKAKNTPVTLS
KDQLSGINNIAFSK

>AHA83913.1
MELLIHRSSAIFLTLAINALYLTSSQNITEEFYQSTCSAVSRGYISALRTGWYTSVITIELSNIKETKCN
GTDTKVKLIKQELDKYKNAVTELQLLMQNTPAANNRARREAPQYMNYTINTTKNLNVSISKKRKRRFLGF
LLGVGSAIASGIAVSKVLHLEGEVNKIKNALLSTNKAVVSLSNGVSVLTSKVLDLKNYINNQLLPIVNQQ
SCRISNIETVIEFQQKNSRLLEITREFSVNAGVTTPLSTYMLTNSELLSLINDMPITNDQKKLMSSNVQI
VRQQSYSIMSIIKEEVLAYVVQLPIYGVIDTPCWKLHTSPLCTTNIKEGSNICLTRTDRGWYCDNAGSVS
FFPQADTCKVQSNRVFCDTMNSLTLPSEVSLCNTDIFNSKYDCKIMTSKTDISSSVITSLGAIVSCYGKT
KCTASNKNRGIIKTFSNGCDYVSNKGVDTVSVGNTLYYVNKLEGKNLYVKGEPIINYYDPLVFPSDEFDA
SISQVNEKINQSLAFIRRSDELLHNVNTGKSTTNIMITAIIIVIIVVLLSLIAIGLLLYCKAKNTPVTLS
KDQLSGINNIAFSK

>AHA83924.1
MELLIHRSSAIFLTLAINALYLTSSQNITEEFYQSTCSAVSRGYFSALRTGWYTSVITIELSNIKETKCN
GTDTKVKLIKQELDKYKNAVTELQLLMQNIPAANNRARREAPQYMNYTINTTKNLNVSISKKRKRRFLGF
LLGVGSAIASGIAVSKVLHLEGEVNKIKNALLSTNKAVVSLSNGVSVLTSKVLDLKNYINNQLLPIVNQQ
SCRISNIETVIEFQQKNSRLLEITREFSVNAGVTTPLSTYMLTNSELLSLINDMPITNDQKKLMSSNVQI
VRQQSYSIMSIIKEEVLAYVVQLPIYGVIDTPCWKLHTSPLCTTNIKEGSNICLTRTDRGWYCDNAGSVS
FFPQADTCKVQSNRVFCDTMNSLTLPSEVSLCNTDIFNSKYDCKIMTSKTDISSSVITSLGAIVSCYGKT
KCTASNKNRGIIKTFSNGCDYVSNKGVDTVSVGNTLYYVNKLEGKNFYVKGEPIINYYDPLVFPSDEFDA
SISQVNEKINQSLAFIRRSDELLHNVNTGKSTTNIMITAIIIVIIVVLLSLIAIGLLLYCKAKNTPVTLS
KDQLSGINNIAFSK

>AHA83990.1
MELLIHRSSAIFLTLAINALYLTSSQNITEEFYQSTCSAVSRGYLSALRTGWYTSVITIELSNIKETKCN
GTDTKVKLIKQELDKYKNAVTELQLLMQNTPAVNNRARREAPQYMNYTINTTKNLNVSISKKRKRRFLGF
LLGVGSAIASGIAVSKVLHLEGEVNKIKNALLSTNKAVVSLSNGVSVLTSKVLDLKNYINNQLLPIVNQQ
SCRIFNIETVIEFQQKNSRLLEITREFSVNAGVTTPLSTYMLTNSELLSLINDMPITNDQKKLMSSNVQI
VRQQSYSIMSIIKEEVLAYVVQLPIYGVIDTPCWKLHTSPLCTTNIKEGSNICLTRTDRGWYCDNAGSVS
FFPQADTCKVQSNRVFCDTMNSLTLPSEVSLCNTDIFNSKYDCKIMTSKTDISSSVITSLGAIVSCYGKT
KCTASNKNRGIIKTFSNGCDYVSNKGVDTVSVGNTLYYVNKLEGKNLYVKGEPIINYYDPLVFPSDEFDA
SISQVNEKINQSLAFIRRSDELLHNVNTGKSTTNIMITAIIIVIIVVLLSLIAIGLLLYCKAKNTPVTLS
KDQLSGINNIAFSK

>AHA84012.1
MELLIHRSSAIFLTLAINALYLTSSQNITEEFYQSTCSAVSRGYFSALRTGWYTSVITIELSNIQETKCN
GTDTKVKLIKQELDKYKNAVTELQLLMQNTPAANNRARREAPQYMNHTINTTKNLNVSISKKRKRRFLGF
LLGVGSAIASGIAVSKVLHLEGEVNKIKNALLSTNKAVVSLSNGVSVLTSKVLDLKNYINNQLLPIVNQQ
SCRISNIETVIEFQQKNSRLLEITREFSVNAGVTTPLSTYMLTNSELLSLINDMPITNDQKKLMSSNVQI
VRQQSYSIMSIIKEEVLAYVVQLPIYGVIDTPCWKLHTSPLCTTNIKEGSNICLTRTDRGWYCDNAGSVS
FFPQADTCKVQSNRVFCDTMNSLTLPSEVSLCNTDIFNSKYDCKIMTSKTDISSSVITSLGAIVSCYGKT
KCTASNKNRGIIKTFSNGCDYVSNKGVDTVSVGNTLYYVNKLEGKNLYVKGEPIINYYDPLVFPSDEFDA
SISQVNEKINQSLAFIRRSDELLHNVNTGKSTTNIMMTAIIIVIIVVLLSLIAIGLLLYCKAKNTPVTLS
KDQLSGINNIAFSK

>AHA84056.1
MELLIHRSSAIFLTLAINALYLTSSQNITEEFYQSTCSAVSRGYLSALRTGWYTSVITIELSNIKETKCN
GTDTKVKLIKQELDKYKNAVTELQLLMQNTPAVNNRARREAPQYMNYTINTTKNLNVSISKKRKRRFLGF
LLGVGSAIASGIAVSKVLHLEGEVNKIKNALLSTNKAVVSLSNGVSVLTSKVLDLKNYINNQLLPIVNQQ
SCRISNIETVIEFQQKNSRLLEITREFSVNAGVTTPLSTYMLTNSELLSLINDMPITNDQKKLMSSNVQI
VRQQSYSIMSIIKEEVLAYVVQLPIYGVIDTPCWKLHTSPLCTTNIKEGSNICLTRTDRGWYCDNAGSVS
FFPQADTCKVQSNRVFCDTMNSLTLPSEVSLCNTDIFNSKYDCKIMTSKTDISSSVITSLGAIVSCYGKT
KCTASNKNRGIIKTFSNGCDYVSNKGVDTVSVGNTLYYVNKLEGKNLYVKGEPIINYYDPLVFPSDEFDA
SISQVNEKINQSLAFIRRSDELLHNVNTGKSTTNIMITAIIIVIIVVLLSLIAIGLLLYCKAKNTPVTLS
KDQLSGINNIAFSK

```
>AHA84067.1
MELLIHRSSAIFLTLAINALYLTSSQNITEEFYQSTCSAVSRGYLSALRTGWYTSVITIELSNIKETKCN
GTDTKVKLIKQELDKYKNAVTELQLLMQNTPAANNRARREAPQYMNYTINTTKNLNVSISKKRKRRFLGF
LLGVGSAIASGIAVSKVLHLEGEVNKIKNALLSTNKAVVSLSNGVSVLTSKVLDLKNYINNQLLPIVNQQ
SCRISNIETVIEFQQKNSRLLEITREFSVNAGVTTPLSTYMLTNSELLSLINDMPITNDQKKLMSSNVQI
VRQQSYSIMSIIKEEVLAYVVQLPIYGVIDTPCWKLHTSPLCTTNIKEGSNICLTRTDRGWYCDNAGSVS
FFPQADTCKVQSNRVFCDTMNSLTLPSEVSLCNTDIFNSKYDCKIMTSKTDISSSVITSLGAIVSCYGKT
KCTASNKNRGIIKTFSNGCDYVSNKGVDTVSVGNTLYYVNKLEGKNLYVKGEPIINYYDPLVFPSDEFDA
SISQVNEKINQSLAFIRRSDELLHNVNTGKSTTNIMITAIIIVIIVVLLSLIAIGLLLYCKAKNTPVTLS
KDQLSGINNIAFSK
```

```
>AHA84078.1
MELLIHRSSAIFLTLAINALYLTSSQNITEEFYQSTCSAVSRGYLSALRTGWYTSVITIELSNIKETKCN
GTDTKVKLIKQELDKYKNAVTELQLLMQNTPAANNRARREAPQYMNYTINTTKNLNVSISKKRKRRFLGF
LLGVGSAIASGIAVSKVLHLEGEVNKIKNALLSTNKAVVSLSNGVSVLTSKVLDLKNYINNQLLPIVNQQ
SCRISNIETVIEFQQKNSRLLEITREFSVNAGVTTPLSTYMLTNSELLSLINDMPITNDQKKLMSSNVQI
VRQQSYSIMSIIKEEVLAYVVQLPIYGVIDTPCWKLHTSPLCTTNIKEGSNICLTRTDRGWYCDNAGSVS
FFPQADTCKVQSNRVFCDTMNSLTLPSEVSLCNTDIFNSKYDCKIMTSKTDISSSVITSLGAIVSCYGKT
KCTASNKNRGIIKTFSNGCDYVSNKGVDTVSVGNTLYYVNKLEGKNLYVKGEPIINYYDPLVFPSDEFDA
SISQVNEKINQSLAFIRRSDELLHNVNTGKSTTNIMITVIIIVIIVVLLSLIAIGLLLYCKAKNTPVTLS
KDQLSGINNIAFSK
```

```
>AHA84089.1
MELLIHRSSAIFLTLAINALYLTSSQNITEEFYQSTCSAVSRGYLSALRTGWYTSVITIELSNIKETKCN
GTDTKVKLIKQELDKYKNAVTELQLLMQNTPAANNRARREAPQYMNYTINTTKNLNVSISKKRKRRFLGF
LLGVGSAIASGVAVSKVLHLEGEVNKIKNALLSTNKAVVSLSNGVSVLTSKVLDLKNYINNQLLPIVNQQ
SCRISNIETVIEFQQKNSRLLEITREFSVNAGVTTPLSTYMLTNSELLSLINDMPITNDQKKLMSSNVQI
VRQQSYSIMSIIKEEVLAYVVQLPIYGVIDTPCWKLHTSPLCTTNIKEGSNICLTRTDRGWYCDNAGSVS
FFPQADTCKVQSNRVFCDTMNSLTLPSEVSLCNTDIFNSKYDCKIMTSKTDISSSVITSLGAIVSCYGKT
KCTASNKNRGIIKTFSNGCDYVSNKGVDTVSVGNTLYYVNKLEGKNLYVKGEPIINYYDPLVFPSDEFDA
SISQVNEKINQSLAFIRRSDELLHNVNTGKSTTNIMITVIIIVIIVVLLSLIAIGLLLYCKAKNTPVTLS
KDQLSGINNIAFSK
```

Table S2. *Human Metapneumovirus* fusion protein

>YP_009513268.1<br>MSWKVVIIFSLLITPQHGLKESYLEESCSTITEGYLSVLRTGWYTNVFTLEVGDVENLTCADGPSLIKTE<br>LDLTKSALRELRTVSADQLAREEQIENPRQSRFVLGAIALGVATAAAVTAGVAIAKTIRLESEVTAIKNA<br>LKKTNEAVSTLGNGVRVLATAVRELKDFVSKNLTRAINKNKCDIADLKMAVSFSQFNRRFLNVVRQFSDN<br>AGITPAISLDLMTDAELARAVSNMPTSAGQIKLMLENRAMVRRKGFGFLIGVYGSSVIYMVQLPIFGVID<br>TPCWIVKAAPSCSGKKGNYACLLREDQGWYCQNAGSTVYYPNEKDCETRGDHVFCDTAAGINVAEQSKEC<br>NINISTTNYPCKVSTGRHPISMVALSPLGALVACYKGVSCSIGSNRVGIIKQLNKGCSYITNQDADTVTI<br>DNTVYQLSKVEGEQHVIKGRPVSSSFDPVKFPEDQFNVALDQVFESIENSQALVDQSNRILSSAEKGNTG<br>FIIVIILIAVLGSTMILVSVFIIIKKTKKPTGAPPELSGVTNNGFIPHN
>AHV79975.1<br>MSWKVVIIFSLLITPQHGLKESYLEESCSTITEGYLSVLRTGWYTNVFTLEVGDVENLTCADGPSLIKTE<br>LDLTKSALRELKTVSADQLAREEQIENPRQSRFVLGAIALGVATAAAVTAGVAIAKTIRLESEVTAIKNA<br>LKKTNEAVSTLGNGVRVLATAVRELKDFVSKNLTRAINKNKCDIDDLKMAVSFSQFNRRFLNVVRQFSDN<br>AGITPAISLDLMTDAELARAVSNMPTSAGQIKLMLENRAMVRRKGFGILIGVYGSSVIYMVQLPIFGVID<br>TPCWIVKAAPSCSEKKGNYACLLREDQGWYCQNAGSTVYYPNEKDCETRGDHVFCDTAAGINVAEQSKEC<br>NINISTTNYPCKVSTGRHPISMVALSPLGALVACYKGVSCSIGSNRVGIIKQLNKGCSYITNQDADTVTI<br>DNTVYQLSKVEGEQHVIKGRPVSSSFDPVKFPEDQFNVALDQVFENIENSQALVDQSNRILSSAEKGNTG<br>FIIVIILIAVLGSSMILVSVFIIIKKTKKPTGAPPELSGVTNNGFIPHS
>AHV79966.1<br>MSWKVVIIFSLLITPQHGLKESYLEESCSTITEGYLSVLRTGWYTNVFTLEVGDVENLTCADGPSLIKTE<br>LDLTKSALRELKTVSADQLAREEQIENPRQSRFVLGAIALGVATAAAVTAGVAIAKTIRLESEVTAIKNA<br>LKKTNEAVSTLGNGVRVLATAVRELKDFVSKNLTRAINKNKCDIDDLKMAVSFSQFNRRFLNVVRQFSDN<br>AGITPAISLDLMTDAELARAVSNMPTSAGQIKLMLENRAMVRRKGFGILIGVYGSSVIYMVQLPIFGVID<br>TPCWIVKAAPSCSEKKGNYACLLREDQGWYCQNAGSTVYYPNEKDCETRGDHVFCDTAAGINVAEQSKEC<br>NINISTTNYPCKVSTGRHPISMVALSPLGALVACYKGVSCSIGSNRVGIIKQLNKGCSYITNQDADTVTI<br>DNTVYQLSKVEGEQHVIKGRPVSSSFDPVKFPEDQFNVALDQVFENIENSQALVDQSNRILSSAEKGNTG<br>FIIVIILIAVLGSSMILVSVFIIIKKTKKPTGAPPELSGVTNNGFIPHS
>AHV79957.1<br>MSWKVMIIISLLITPQHGLKESYLEESCSTITEGYLSVLRTGWYTNVFTLEVGDVENLTCTDGPSLIKTE<br>LDLTKSALRELKTVSADQLAREEQIENPRQSRFVLGAIALGVATAAAVTAGIAIAKTIRLESEVNAIKGA<br>LKQTNEAVSTLGNGVRVLATAVRELKEFVSKNLTSAINRNKCDIADLKMAVSFSQFNRRFLNVVRQFSDN<br>AGITPAISLDLMTDAELARAVSYMPTSAGQIKLMLENRAMVRRKGFGILIGVYGSSVIYMVQLPIFGVID<br>TPCWIIKAAPSCSEKNGNYACLLREDQGWYCKNAGSTVYYPNEKDCETRGDHVFCDTAAGINVAEQSREC<br>NINISTTNYPCKVSTGRHPISMVALSPLGALVACYKGVSCSIGSNRVGIIKQLPKGCSYITNQDADTVTI<br>DNTVYQLSKVEGEQHVIKGRPVSSSFDPIRFPEDQFNVALDQVFESIENSQALVEQSNKILNSAEKGNTG<br>FIIVIILVAVLGLTMISVSIIIIIKKTRKPTGAPPELNGVTNGGFIPHS
>AHV79948.1<br>MSWKVVIIFSLLITPQHGLKESYLEESCSTITEGYLSVLRTGWYTNVFTLEVGDVENLTCADGPSLIKTE<br>LDLTKSALRELKTVSADQLAREEQIENPRQSRFVLGAIALGVATAAAVTAGVAIAKTIRLESEVTAIKNA<br>LKKTNEAVSTLGNGVRVLATAVRELKDFVSKNLTRAINKNKCDIDDLKMAVSFSQFNRRFLNVVRQFSDN<br>AGITPAISLDLMTDAELARAVSNMPTSAGQIKLMLENRAMVRRKGFGILIGVYGSSVIYMVQLPIFGVID<br>TPCWIVKAAPSCSEKKGNYACLLREDQGWYCQNAGSTVYYPNEKDCETRGDHVFCDTAAGINVAEQSKEC<br>NINISTTNYPCKVSTGRHPISMVALSPLGALVACYKGVSCSIGSNRVGIIKQLNKGCSYITNQDADTVTI<br>DNTVYQLSKVEGEQHVIKGRPVSSSFDPVKFPEDQFNVALDQVFENIENSQALVDQSNRILSSAEKGNTG<br>FIIVIILIAVLGSSMILVSVFIIIKKTKKPTGAPPELSGVTNNGFIPHS

>AHV79939.1]
MSWKVVIIFSLLITPQHGLKESYLEESCSTITEGYLSVLRTGWYTNVFTLEVGDVENLTCSDGPSLIKTE
LDLTKSALRELKTVSADQLAREEQIENPRQSRFVLGAIALGVATAAAVTAGVAIAKTIRLEGEVTAIKNA
LKTTNEAVSTLGNGVRVLATAVRELKDFVSKNLTRAINKNKCDIDDLKMAVSFSQFNRRFLNVVRQFSDN
AGITPAISLDLMTDAELARAVSNMPTSAGQIKLMLENRAMVRRKGFGILIGVYGSSVIYMVQLPIFGVID
TPCWIVKAAPSCSEKKGNYACLLREDQGWYCQNAGSTVYYPNEKDCETRGDHVFCDTAAGINVAEQSKEC
NINISTTNYPCKVSTGRHPISMVALSPLGALVACYKGVSCSIGSNRVGIIKQLNKGCSYITNQDADTVTI
DNTVYQLSKVEGEQHVIKGRPVSSSFDPIKFPEDQFNVALDQVFENIENSQALVDQSNRILSSAEKGNTG
FIIVIILIAVLGSSMILVSIFIIIKKTKKPTGAPPELSGVTNNGFIPHS

7. >AHV79930.1 fusion glycoprotein [human metapneumovirus]
MSWKVMIIISLLITPQHGLKESYLEESCSTITEGYLSVLRTGWYTNVFTLEVGDVENLTCTDGPSLIKTE
LDLTKSALRELKTVSADQLAREEQIENPRQSRFVLGAIALGVATAAAVTAGIAIAKTIRLESEVNAIKGA
LKTTNEAVSTLGNGVRVLATAVRELKEFVSKNLTSAINKNKCDIADLKMAVSFSQFNRRFLNVVRQFSDN
AGITPAISLDLMNDAELARAVSYMPTSAGQIKLMLENRAMVRRKGFGILIGVYGSSVIYMVQLPIFGVIN
TPCWIIKAAPSCSEKDGNYACLLREDQGWYCKNAGSTVYYPNEKDCETRGDHVFCDTAAGINVAEQSREC
NINISTTNYPCKVSTGRHPISMVALSPLGALVACYKGVSCSTGSNQVGIIKQLPKGCSYITNQDADTVTI
DNTVYQLSKVEGEQHVIKGRPVSSSFDPIRFPEDQFNVALDQVFESIENSQALVDQSNKILNSAEKGNTG
FIIVIILIAVLGLTMISVSIIIIIKKTRKPTGAPPELNGVTNGGFIPHS

>AHV79921.1
MSWKVMIIISLLITPQHGLKESYLEESCSTITEGYLSVLRTGWYTNVFTLEVGDVENLTCTDGPSLIKTE
LDLTKSALRELKTVSADQLAREEQIENPRQSRFVLGAIALGVATAAAVTAGIAIAKTIRLESEVNAIKGA
LKQTNEAVSTLGNGVRVLATAVRELKEFVSKNLTSAINRNKCDIADLKMAVSFSQFNRRFLNVVRQFSDN
AGITPAISLDLMTDAELARAVSYMPTSAGQIKLMLENRAMVRRKGFGILIGVYGSSVIYMVQLPIFGVID
TPCWIIKAAPSCSEKNGNYACLLREDQGWYCKNAGSTVYYPNEKDCETRGDHVFCDTAAGINVAEQSREC
NINISTTNYPCKVSTGRHPISMVALSPLGALVACYKGVSCSIGSNRVGIIKQLPKGCSYITNQDADTVTI
DNTVYQLSKVEGEQHVIKGRPVSSSFDPIRFPEDQFNVALDQVFESIENSQALVEQSNKILNSAEKGNTG
FIIVIILVAVLGLTMISVSIIIIIKKTRKPTGAPPELNGVTNGGFIPHS

>AHV79912.1
MSWKVVIIFSLLITPQHGLKESYLEESCSTITEGYLSVLRTGWYTNVFTLEVGDVENLTCADGPSLIKTE
LDLTKSALRELKTVSADQLAREEQIENPRQSRFVLGAIALGVATAAAVTAGVAIAKTIRLESEVTAIKNA
LKKTNEAVSTLGNGVRVLATAVRELKDFVSKNLTRAINKNKCDIDDLKMAVSFSQFNRRFLNVVRQFSDN
AGITPAISLDLMTDAELARAVSNMPTSAGQIKLMLENRAMVRRKGFGILIGVYGSSVIYMVQLPIFGVID
TPCWIVKAAPSCSEKKGNYACLLREDQGWYCQNAGSTVYYPNEKDCETRGDHVFCDTAAGINVAEQSKEC
NINISTTNYPCKVSTGRHPISMVALSPLGALVACYKGVSCSIGSNRVGIIKQLNKGCSYITNQDADTVTI
DNTVYQLSKVEGEQHVIKGRPVSSSFDPVKFPEDQFNVALDQVFENIENSQALVDQSNRILSSAEKGNTG
FIIVIILIAVLGSSMILVSVFIIIKKTKKPTGAPPELSGVTNNGFIPHS

>AHV79903.1
MSWKVVIIFSLLITPQHGLKESYLEESCSTITEGYLSVLRTGWYTNVFTLEVGDVENLTCADGPSLIKTE
LDLTKSALRELKTVSADQLAREEQIENPRQSRFVLGAIALGVATAAAVTAGVAIAKTIRLESEVTAIKNA
LKKTNEAVSTLGNGVRVLATAVRELKDFVSKNLTRAINKNKCDIDDLKMAVSFSQFNRRFLNVVRQFSDN
AGITPAISLDLMTDAELARAVSNMPTSAGQIKLMLENRAMVRRKGFGILIGVYGSSVIYMVQLPIFGVID
TPCWIVKAAPSCSEKKGNYACLLREDQGWYCQNAGSTVYYPNEKDCETRGDHVFCDTAAGINVAEQSKEC
NINISTTNYPCKVSTGRHPISMVALSPLGALVACYKGVSCSIGSNRVGIIKQLNKGCSYITNQDADTVTI
DNTVYQLSKVEGEQHVIKGRPVSSSFDPVKFPEDQFNVALDQVFENIENSQALVDQSNRILSSAEKGNTG
FIIVIILIAVLGSSMILVSVFIIIKKTKKPTGAPPELSGVTNNGFIPHS

>AHV79894.1
MSWKVVIIFSLLITPQHGLKESYLEESCSTITEGYLSVLRTGWYTNVFTLEVGDVENLTCSDGPSLIKTE
LDLTKSALRELKTVSADQLAREEQIENPRQSRFVLGAIALGVATAAAVTAGVAIAKTIRLEGEVTAIKNA
LKTTNEAVSTLGNGVRVLATAVRELKDFVSKNLTRAINKNKCDIDDLKMAVSFSQFNRRFLNVVRQFSDN
AGITPAISLDLMTDAELARAVSNMPTSAGQIKLMLENRAMVRRKGFGILIGVYGSSVIYMVQLPIFGVID
TPCWIVKAAPSCSEKKGNYACLLREDQGWYCQNAGSTVYYPNEKDCETRGDHVFCDTAAGINVAEQSKEC
NINISTTNYPCKVSTGRHPISMVALSPLGALVACYKGVSCSIGSNRVGIIKQLNKGCSYITNQDADTVTI
DNTVYQLSKVEGEQHVIKGRPVSSSFDPIKFPEDQFNVALDQVFENIENSQALVDQSNRILSSAEKGNTG
FIIVIILIAVLGSSMILVSIFIIIKKTKKPTGAPPELSGVTNNGFIPHS

>AHV79885.1
MSWKVVIIFSLLITPQHGLKESYLEESCSTITEGYLSVLRTGWYTNVFTLEVGDVENLTCSDGPSLIKTE
LDLTKSALRELKTVSADQLAREEQIENPRQSRFVLGAIALGVATAAAVTAGVAIAKTIRLEGEVTAIKNA
LKTTNEAVSTLGNGVRVLATAVRELKDFVSKNLTRAINKNKCDIDDLKMAVSFSQFNRRFLNVVRQFSDN
AGITPAISLDLMTDAELARAVSNMPTSAGQIKLMLENRAMVRRKGFGILIGVYGSSVIYMVQLPIFGVID
TPCWIVKAAPSCSEKKGNYACLLREDQGWYCQNAGSTVYYPNEKDCETRGDHVFCDTAAGINVAEQSKEC
NINISTTNYPCKVSTGRHPISMVALSPLGALVACYKGVSCSIGSNRVGIIKQLNKGCSYITNQDADTVTI
DNTVYQLSKVEGEQHVIKGRPVSSSFDPIKFPEDQFNVALDQVFENIENSQALVDQSNRILSSAEKGNTG
FIIVIILIAVLGSSMILVSIFIIIKKTKKPTGAPPELSGVTNNGFIPHS

>AHV79876.1
MSWKVVIIFSLLITPQHGLKESYLEESCSTITEGYLSVLRTGWYTNVFTLEVGDVENLTCADGPSLIKTE
LDLTKSALRELKTVSADQLAREEQIENPRQSRFVLGAIALGVATAAAVTAGVAIAKTIRLESEVTAIKNA
LKKTNEAVSTLGNGVRVLATAVRELKDFVSKNLTRAINKNKCDIDDLKMAVSFSQFNRRFLNVVRQFSDN
AGITPAISLDLMTDAELARAVSNMPTSAGQIKLMLENRAMVRRKGFGILIGVYGSSVIYMVQLPIFGVID
TPCWIVKAAPSCSEKKGNYACLLREDQGWYCQNAGSTVYYPNEKDCETRGDHVFCDTAAGINVAEQSKEC
NINISTTNYPCKVSTGRHPISMVALSPLGALVACYKGVSCSIGSNRVGIIKQLNKGCSYITNQDADTVTI
DNTVYQLSKVEGEQHVIKGRPVSSSFDPVKFPEDQFNVALDQVFENIENSQALVDQSNRILSSAEKGNTG
FIIVIILIAVLGSSMILVSVFIIIKKTKKPTGAPPELSGVTNNGFIPHS

>AHV79867.1
MSWKVVIIFSLLITPQHGLKESYLEESCSTITEGYLSVLRTGWYTNVFTLEVGDVENLTCSDGPSLIKTE
LDLTKSALRELKTVSADQLAREEQIENPRQSRFVLGAIALGVATAAAVTAGVAIAKTIRLEGEVTAIKNA
LKTTNEAVSTLGNGVRVLATAVRELKDFVSKNLTRAINKNKCDIDDLKMAVSFSQFNRRFLNVVRQFSDN
AGITPAISLDLMTDAELARAVSNMPTSAGQIKLMLENRAMVRRKGFGILIGVYGSSVIYMVQLPIFGVID
TPCWIVKAAPSCSEKRGNYACLLREDQGWYCQNAGSTVYYPNEKDCETRGDHVFCDTAAGINVAEQSKEC
NINISTTNYPCKVSTGRHPISMVALSPLGALVACYKGVSCSIGSNRVGIIKQLNKGCSYITNQDADTVTI
DNTVYQLSKVEGEQHVIKGRPVSSSFDPIKFPEDQFNVALDQVFENIENSQALVDQSNRILSSAEKGNTG
FIIVIILIAVLGSSMILVSIFIIIKKTKKPTGAPPELSGVTNNGFIPHS

>AHV79858.1
MSWKVVIIFSLLITPQHGLKESYLEESCSTITEGYLSVLRTGWYTNVFTLEVGDVENLTCADGPSLIKTE
LDLTKSALRELKTVSADQLAREEQIENPRQSRFVLGAIALGVATAAAVTAGVAIAKTIRLESEVTAIKNA
LKKTNEAVSTLGNGVRVLATAVRELKDFVSKNLTRAINKNKCDIDDLKMAVSFSQFNRRFLNVVRQFSDN
AGITPAISLDLMTDAELARAVSNMPTSAGQIKLMLENRAMVRRKGFGILIGVYGSSVIYMVQLPIFGVID
TPCWIVKAAPSCSEKKGNYACLLREDQGWYCQNAGSTVYYPNEKDCETRGDHVFCDTAAGINVAEQSKEC
NINISTTNYPCKVSTGRHPISMVALSPLGALVACYKGVSCSIGSNRVGIIKQLNKGCSYITNQDADTVTI
DNTVYQLSKVEGEQHVIKGRPVSSSFDPVKFPEDQFNVALDQVFENIENSQALVDQSNRILSSAEKGNTG
FIIVIILIAVLGSSMILVSVLIIIKKTKKPTGAPPELSGVTNNGFIPHS

>AHV79849.1
MSWKVVIIFSLLITPQHGLKESYLEESCSTITEGYLSVLRTGWYTNVFTLEVGDVENLTCSDGPSLIKTE
LDLTKSALRELKTVSADQLAREEQIENPRQSRFVLGAIALGVATAAAVTAGVAIAKTIRLEGEVTAIKNA
LKTTNEAVSTLGNGVRVLATAVRELKDFVSKNLTRAINKNKCDIDDLKMAVSFSQFNRRFLNVVRQFSDN
AGITPAISLDLMTDAELARAVSNMPTSAGQIKLMLENRAMVRRKGFGILIGVYGSSVIYMVQLPIFGVID
TPCWIVKAAPSCSEKKGNYACLLREDQGWYCQNAGSTVYYPNEKDCETRGDHVFCDTAAGINVAEQSKEC
NINISTTNYPCKVSTGRHPISMVALSPLGALVACYKGVSCSIGSNRVGIIKQLNKGCSYITNQDADTVTI
DNTVYQLSKVEGEQHVIKGRPVSSSFDPIKFPEDQFNVALDQVFENIENSQALVDQSNRILSSAEKGNTG
FIIVIILIAVLGSSMILVSIFIIIKKTKKPTGAPPELSGVTNNGFIPHS

>AHV79840.1
MSWKVVIIFSLLITPQHGLKESYLEESCSTITEGYLSVLRTGWYTNVFTLEVGDVENLTCADGPSLIKTE
LDLTKSALRELKTVSADQLAREEQIENPRQSRFVLGAIALGVATAAAVTAGVAIAKTIRLESEVTAIKNA
LKKTNEAVSTLGNGVRVLATAVRELKDFVSKNLTRAINKNKCDIDDLKMAVSFSQFNRRFLNVVRQFSDN
AGITPAISLDLMTDAELARAVSNMPTSAGQIKLMLENRAMVRRKGFGILIGVYGSSVIYMVQLPIFGVID
TPCWIVKAAPSCSEKKGNYACLLREDQGWYCQNAGSTVYYPNEKDCETRGDHVFCDTAAGINVAEQSKEC
NINISTTNYPCKVSTGRHPISMVALSPLGALVACYKGVSCSIGSNRVGIIKQLNKGCSYITNQDADTVTI
DNTVYQLSKVEGEQHVIKGRPVSSSFDPVKFPEDQFNVALDQVFENIENSQALVDQSNRILSSAEKGNTG
FIIVIILIAVLGSSMILVSVFIIIKKTKKPTGAPPELSGVTNNGFIPHS

>AHV79840.1
MSWKVVIIFSLLITPQHGLKESYLEESCSTITEGYLSVLRTGWYTNVFTLEVGDVENLTCADGPSLIKTE
LDLTKSALRELKTVSADQLAREEQIENPRQSRFVLGAIALGVATAAAVTAGVAIAKTIRLESEVTAIKNA
LKKTNEAVSTLGNGVRVLATAVRELKDFVSKNLTRAINKNKCDIDDLKMAVSFSQFNRRFLNVVRQFSDN
AGITPAISLDLMTDAELARAVSNMPTSAGQIKLMLENRAMVRRKGFGILIGVYGSSVIYMVQLPIFGVID
TPCWIVKAAPSCSEKKGNYACLLREDQGWYCQNAGSTVYYPNEKDCETRGDHVFCDTAAGINVAEQSKEC
NINISTTNYPCKVSTGRHPISMVALSPLGALVACYKGVSCSIGSNRVGIIKQLNKGCSYITNQDADTVTI
DNTVYQLSKVEGEQHVIKGRPVSSSFDPVKFPEDQFNVALDQVFENIENSQALVDQSNRILSSAEKGNTG
FIIVIILIAVLGSSMILVSVFIIIKKTKKPTGAPPELSGVTNNGFIPHS

>AHV79822.1
MSWKVVIIFSLLITPQHGLKESYLEESCSTITEGYLSVLRTGWYTNVFTLEVGDVENLTCADGPSLIKTE
LDLTKSALRELKTVSADQLAREEQIENPRQSRFVLGAIALGVATAAAVTAGVAIAKTIRLESEVTAIKNA
LKKTNEAVSTLGNGVRVLATAVRELKDFVSKNLTRAINKNKCDIDDLKMAVSFSQFNRRFLNVVRQFSDN
AGITPAISLDLMTDAELARAVSNMPTSAGQIKLMLENRAMVRRKGFGILIGVYGSSVIYMVQLPIFGVID
TPCWIVKAAPSCSEKKGNYACLLREDQGWYCQNAGSTVYYPNEKDCETRGDHVFCDTAAGINVAEQSKEC
NINISTTNYPCKVSTGRHPISMVALSPLGALVACYKGVSCSIGSNRVGIIKQLNKGCSYITNQDADTVTI
DNTVYQLSKVEGEQHVIKGRPVSSSFDPVKFPEDQFNVALDQVFENIENSQALVDQSNRILSSAEKGNTG
FIIVIILIAVLGSSMILVSVFIIIKKTKKPTGAPPELSGVTNNGFIPHS

>AHV79813.1
MSWKVVIIFSLLITPQHGLKESYLEESCSTITEGYLSVLRTGWYTNVFTLEVGDVENLTCSDGPSLIKTE
LDLTKSALRELKTVSADQLAREEQIENPRQSRFVLGAIALGVATAAAVTAGVAIAKTIRLEGEVTAIKNA
LKTTNEAVSTLGNGVRVLATAVRELKDFVSKNLTRAINKNKCDIDDLKMAVSFSQFNRRFLNVVRQFSDN
AGITPAISLDLMTDAELARAVSNMPTSAGQIKLMLENRAMVRRKGFGILIGVYGSSVIYMVQLPIFGVID
TPCWIVKAAPSCSEKKGNYACLLREDQGWYCQNAGSTVYYPNEKDCETRGDHVFCDTAAGINVAEQSKEC
NINISTTNYPCKVSTGRHPISMVALSPLGALVACYKGVSCSIGSNRVGIIKQLNKGCSYITNQDADTVTI
DNTVYQLSKVEGEQHVIKGRPVSSSFDPIKFPEDQFNVALDQVFENIENSQALVDQSNRILSSAEKGNTG
FIIVIILIAVLGSSMILVSIFIIIKKTKKPTGAPPELSGVTNNGFIPHS

>AHV79804.1
MSWKVVIIFSLLITPQHGLKESYLEESCSTITEGYLSVLRTGWYTNVFTLEVGDVENLTCSDGPSLIKTE
LDLTKSALRELKTVSADQLAREEQIENPRQSRFVLGAIALGVATAAAVTAGVAIAKTIRLEGEVTAIKNA
LKTTNEAVSTLGNGVRVLATAVRELKDFVSKNLTRAINKNKCDIDDLKMAVSFSQFNRRFLNVVRQFSDN
AGITPAISLDLMTDAELARAVSNMPTSAGQIKLMLENRAMVRRKGFGILIGVYGSSVIYMVQLPIFGVID
TPCWIVKAAPSCSEKKGNYACLLREDQGWYCQNAGSTVYYPNEKDCETRGDHVFCDTAAGINVAEQSKEC
NINISTTNYPCKVSTGRHPISMVALSPLGALVACYKGVSCSIGSNRVGIIKQLNKGCSYITNQDADTVTI
DNTVYQLSKVEGEQHVIKGRPVSSSFDPIKFPEDQFNVALDQVFENIENSQALVDQSNRILSSAEKGNTG
FIIVIILIAVLGSSMILVSIFIIIKKTKKPTGAPPELSGVTNNGFIPHS

>AHV79795.1
MSWKVVIIFSLLITPQHGLKESYLEESCSTITEGYLSVLRTGWYTNVFTLEVGDVENLTCADGPSLIKTE
LDLTKSALRELKTVSADQLAREEQIENPRQSRFVLGAIALGVATAAAVTAGVAIAKTIRLESEVTAIKNA
LKKTNEAVSTLGNGVRVLATAVRELKDFVSKNLTRAINKNKCDIDDLKMAVSFSQFNRRFLNVVRQFSDN
AGITPAISLDLMTDAELARAVSNMPTSAGQIKLMLENRAMVRRKGFGILIGVYGSSVIYMVQLPIFGVID
TPCWIVKAAPSCSEKKGNYACLLREDQGWYCQNAGSTVYYPNEKDCETRGDHVFCDTAAGINVAEQSKEC
NINISTTNYPCKVSTGRHPISMVALSPLGALVACYKGVSCSIGSNRVGIIKQLSKGCSYITNQDADTVTI
DNTVYQLSKVEGEQHVIKGRPVSSSFDPVKFPEDQFNVALDQVFENIENSQALVDQSNRILSSAEKGNTG
FIIVIILIAVLGSSMILVSVFIIIKKTKKPTGAPPELSGVTNNGFIPHS

>AHV79786.1
MSWKVVIIFSLLITPQHGLKESYLEESCSTITEGYLSVLRTGWYTNVFTLEVGDVENLTCSDGPSLIKTE
LDLTKSALRELKTVSADQLAREEQIENPRQSRFVLGAIALGVATAAAVTAGVAIAKTIRLEGEVTAIKNA
LKTTNEAVSTLGNGVRVLATAVRELKDFVSKNLTRAINKNKCDIDDLKMAVSFSQFNRRFLNVVRQFSDN
AGITPAISLDLMTDAELARAVSNMPTSAGQIKLMLENRAMVRRKGFGILIGVYGSSVIYMVQLPIFGVID
TPCWIVKAAPSCSEKKGNYACLLREDQGWYCQNAGSTVYYPNEKDCETRGDHVFCDTAAGINVAEQSKEC
NINISTTNYPCKVSTGRHPISMVALSPLGALVACYKGVSCSIGSNRVGIIKQLNKGCSYITNQDADTVTI
DNTVYQLSKVEGEQHVIKGRPVSSSFDPIKFPEDQFNVALDQVFENIENSQALVDQSNRILSSAEKGNTG
FIIVIILIAVLGSSMILVSIFIIIKKTKKPTGAPPELSGVTNNGFIPHS

>AHV79777.1
MSWKVVIIFSLLITPQHGLKESYLEESCSTITEGYLSVLRTGWYTNVFTLEVGDVENLTCSDGPSLIKTE
LDLTKSALRELKTVSADQLAREEQIENPRQSRFVLGAIALGVATAAAVTAGVAIAKTIRLEGEVTAIKNA
LKTTNEAVSTLGNGVRVLATAVRELKDFVSKNLTRAINKNKCDIDDLKMAVSFSQFNRRFLNVVRQFSDN
AGITPAISLDLMTDAELARAVSNMPTSAGQIKLMLENRAMVRRKGFGILIGVYGSSVIYMVQLPIFGVID
TPCWIVKAAPSCSEKKGNYACLLREDQGWYCQNAGSTVYYPNEKDCETRGDHVFCDTAAGINVAEQSKEC
NINISTTNYPCKVSTGRHPISMVALSPLGALVACYKGVSCSIGSNRVGIIKQLNKGCSYITNQDADTVTI
DNTVYQLSKVEGEQHVIKGRPVSSSFDPIKFPEDQFNVALDQVFENIENSQALVDQSNRILSSAEKGNTG
FIIVIILIAVLGSSMILVSIFIIIKKTKKPTGAPPELSGVTNNGFIPHS

>AHV79759.1
MSWKVVIIFSLLITPQHGLKESYLEESCSTITEGYLSVLRTGWYTNVFTLEVGDVENLTCSDGPSLIKTE
LDLTKSALRELKTVSADQLAREEQIENPRQSRFVLGAIALGVATAAAVTAGVAIAKTIRLEGEVTAIKNA
LKTTNEAVSTLGNGVRVLATAVRELKDFVSKNLTRAINKNKCDIDDLKMAVSFSQFNRRFLNVVRQFSDN
AGITPAISLDLMTDAELARAVSNMPTSAGQIKLMLENRAMVRRKGFGILIGVYGSSVIYMVQLPIFGVID
TPCWIVKAAPSCSEKKGNYACLLREDQGWYCQNAGSTVYYPNEKDCETRGDHVFCDTAAGINVAEQSKEC
NINISTTNYPCKVSTGRHPISMVALSPLGALVACYKGVSCSIGSNRVGIIKQLNKGCSYITNQDADTVTI
DNTVYQLSKVEGEQHVIKGRPVSSSFDPIKFPEDQFNVALDQVFENIENSQALVDQSNRILSSAEKGNTG
FIIVIILIAVLGSSMILVSIFIIIKKTKKPTGAPPELSGVTNNGFIPHS

>AHV79750.1
MSWKVVIIFSLLITPQHGLKESYLEESCSTITEGYLSVLRTGWYTNVFTLEVGDVENLTCADGPSLIKTE
LDLTKSALRELKTVSADQLAREEQIENPRQSRFVLGAIALGVATAAAVTAGVAIAKTIRLESEVTAIKNA
LKKTNEAVSTLGNGVRVLATAVRELKDFVSKNLTRAINKNKCDIDDLKMAVSFSQFNRRFLNVVRQFSDN
AGITPAISLDLMTDAELARAVSNMPTSAGQIKLMLENRAMVRRKGFGILIGVYGSSVIYMVQLPIFGVID
TPCWIVKAAPSCSEKKGNYACLLREDQGWYCQNAGSTVYYPNEKDCETRGDHVFCDTAAGINVAEQSKEC
NINISTTNYPCKVSTGRHPISMVALSPLGALVACYKGVSCSIGSNRVGIIKQLNKGCSYITNQDADTVTI
DNTVYQLSKVEGEQHVIKGRPVSSSFDPVKFPEDQFNVALDQVFENIENSQALVDQSNRILSSAEKGNTG
FIIVIILIAVLGSSMILVSVFIIIKKTKKPTGAPPELSGVTNNGFIPHS

>AHV79741.1
MSWKVVVIFSLLITPQHGLKESYLEESCSTITEGYLSVLRTGWYTNVFTLEVGDVENLTCADGPSLIKTE
LDLTKSALRELKTVSADQLAREEQIENPRQSRFVLGAIALGVATAAAVTAGVAIAKTIRLESEVTAIKNA
LKKTNEAVSTLGNGVRVLATAVRELKDFVSKNLTRAINKNKCDIDDLKMAVSFSQFNRRFLNVVRQFSDN
AGITPAISLDLMTDAELARAVSNMPTSAGQIKLMLENRAMVRRKGFGILIGVYGSSVIYMVQLPIFGVID
TPCWIVKAAPSCSEKKGNYACLLREDQGWYCQNAGSTVYYPNEKDCETRGDHVFCDTAAGINVAEQSKEC
NINISTTNYPCKVSTGRHPISMVALSPLGALVACYKGVSCSIGSNRVGIIKQLNKGCSYITNQDADTVTI
DNTVYQLSKVEGEQHVIKGRPVSSSFDPVKFPEDQFNVALDQVFENIENSQALVDQSNRILSSAEKGNTG
FIIVIILIAVLGSSMILVSVFIIIKKTKKPTGAPPELSGVTNNGFIPHS

>AHV79732.1
MSWKVVIIFSLLITPQHGLKESYLEESCSTITEGYLSVLRTGWYTNVFTLEVGDVENLTCADGPSLIKTE
LDLTKSALRELKTVSADQLAREEQIENPRQSRFVLGAIALGVATAAAVTAGVAIAKTIRLESEVTAIKNA
LKKTNEAVSTLGNGVRVLATAVRELKDFVSKNLTRAINKNKCDIDDLKMAVSFSQFNRRFLNVVRQFSDN
AGITPAISLDLMTDAELARAVSNMPTSAGQIKLMLENRAMVRRKGFGILIGVYGSSVIYMVQLPIFGVID
TPCWIVKAAPSCSEKKGNYACLLREDQGWYCQNAGSTVYYPNEKDCETRGDHVFCDTAAGINVAEQSKEC
NINISTTNYPCKVSTGRHPISMVALSPLGALVACYKGVSCSIGSNRVGIIKQLNKGCSYITNQDADTVTI
DNTVYQLSKVEGEQHVIKGRPVSSSFDPVKFPEDQFNVALDQVFENIENSQALVDQSNRILSSAEKGNTG
FIIVIILIAVLGSSMILVSVFIIIKKTKKPTGAPPELSGVTNNGFIPHS

>AHV79723.1
MSWKVVIIFSLLITPQHGLKESYLEESCSTITEGYLSVLRTGWYTNVFTLEVGDVENLTCADGPSLIKTE
LDLTKSALRELKTVSADQLAREEQIENPRQSRFVLGAIALGVATAAAVTAGVAIAKTIRLESEVTAIKNA
LKKTNEAVSTLGNGVRVLATAVRELKDFVSKNLTRAINKNKCDIDDLKMAVSFSQFNRRFLNVVRQFSDN
AGITPAISLDLMTDAELARAVSNMPTSAGQIKLMLENRAMVRRKGFGILIGVYGSSVIYMVQLPIFGVID
TPCWIVKAAPSCSEKKGNYACLLREDQGWYCQNAGSTVYYPNEKDCETRGDHVFCDTAAGINVAEQSKEC
NINISTTNYPCKVSTGRHPISMVALSPLGALVACYKGVSCSIGSNRVGIIKQLNKGCSYITNQDADTVTI
DNTVYQLSKVEGEQHVIKGRPVSSSFDPVKFPEDQFNVALDQVFENIENSQALVDQSNRILSSAEKGNTG
FIIVIILIAVLGSSMILVSVFIIIKKTKKPTGAPPELSGVTNNGFIPHS

>AHV79714.1
MSWKVVIIFSLLITPQHGLKESYLEESCSTITEGYLSVLRTGWYTNVFTLEVGDVENLTCADGPSLIKTE
LDLTKSALRELKTVSADQLAREEQIENPRQSRFVLGAIALGVATAAAVTAGVAIAKTIRLESEVTAIKNA
LKKTNEAVSTLGNGVRVLATAVRELKDFVSKNLTRAINKNKCDIDDLKMAVSFSQFNRRFLNVVRQFSDN
AGITPAISLDLMTDAELARAVSNMPTSAGQIKLMLENRAMVRRKGFGILIGVYGSSVIYMVQLPIFGVID
TPCWIVKAAPSCSEKKGNYACLLREDQGWYCQNAGSTVYYPNEKDCETRGDHVFCDTAAGINVAEQSKEC
NINISTTNYPCKVSTGRHPISMVALSPLGALVACYKGVSCSIGSNRVGIIKQLNKGCSYITNQDADTVTI
DNTVYQLSKVEGEQHVIKGRPVSSSFDPVKFPEDQFNVALDQVFENIENSQALVDQSNRILSSAEKGNTG
FIIVIILIAVLGSSMILVSVFIIIKKTKKPTGAPPELSGVTNNGFIPHS

>AHV79714.1
MSWKVVIIFSLLITPQHGLKESYLEESCSTITEGYLSVLRTGWYTNVFTLEVGDVENLTCADGPSLIKTE
LDLTKSALRELKTVSADQLAREEQIENPRQSRFVLGAIALGVATAAAVTAGVAIAKTIRLESEVTAIKNA
LKKTNEAVSTLGNGVRVLATAVRELKDFVSKNLTRAINKNKCDIDDLKMAVSFSQFNRRFLNVVRQFSDN
AGITPAISLDLMTDAELARAVSNMPTSAGQIKLMLENRAMVRRKGFGILIGVYGSSVIYMVQLPIFGVID
TPCWIVKAAPSCSEKKGNYACLLREDQGWYCQNAGSTVYYPNEKDCETRGDHVFCDTAAGINVAEQSKEC
NINISTTNYPCKVSTGRHPISMVALSPLGALVACYKGVSCSIGSNRVGIIKQLNKGCSYITNQDADTVTI

DNTVYQLSKVEGEQHVIKGRPVSSSFDPVKFPEDQFNVALDQVFENIENSQALVDQSNRILSSAEKGNTG<br>FIIVIILIAVLGSSMILVSVFIIIKKTKKPTGAPPELSGVTNNGFIPHS
>AHV79696.1<br>MSWKVVIIFSLLITPQHGLKESYLEESCSTITEGYLSVLRTGWYTNVFTLEVGDVENLTCADGPSLIKTE<br>LDLTKSALRELKTVSADQLAREEQIENPRQSRFVLGAIALGVATAAAVTAGVAIAKTIRLESEVTAIKNA<br>LKKTNEAVSTLGNGVRVLATAVRELKDFVSKNLTRAINKNKCDIDDLKMAVSFSQFNRRFLNVVRQFSDN<br>AGITPAISLDLMTDAELARAVSNMPTSAGQIKLMLENRAMVRRKGFGILIGVYGSSVIYMVQLPIFGVID<br>TPCWIVKAAPSCSEKKGNYACLLREDQGWYCQNAGSTVYYPNEKDCETRGDHVFCDTAAGINVAEQSKEC<br>NINISTTNYPCKVSTGRHPISMVALSPLGALVACYKGVSCSIGSNRVGIIKQLNKGCSYITNQDADTVTI<br>DNTVYQLSKVEGEQHVIKGRPVSSSFDPVKFPEDQFNVALDQVFENIENSQALVDQSNRILSSAEKGNTG<br>FIIVIILIAVLGSSMILVSVFIIIKKTKKPTGAPPELSGVTNNGFIPHS
>AHV79687.1<br>MSWKVVIIFSLLITPQHGLKESYLEESCSTITEGYLSVLRTGWYTNVFTLEVGDVENLTCADGPSLIKTE<br>LDLTKSALRELKTVSADQLAREEQIENPRQSRFVLGAIALGVATAAAVTAGVAIAKTIRLESEVTAIKNA<br>LKKTNEAVSTLGNGVRVLATAVRELKDFVSKNLTRAINKNKCDIDDLKMAVSFSQFNRRFLNVVRQFSDN<br>AGITPAISLDLMTDAELARAVSNMPTSAGQIKLMLENRAMVRRKGFGILIGVYGSSVIYMVQLPIFGVID<br>TPCWIVKAAPSCSEKKGNYACLLREDQGWYCQNAGSTVYYPNEKDCETRGDHVFCDTAAGINVAEQSKEC<br>NINISTTNYPCKVSTGRHPISMVALSPLGALVACYKGVSCSIGSNRVGIIKQLNKGCSYITNQDADTVTI<br>DNTVYQLSKVEGEQHVIKGRPVSSSFDPVKFPEDQFNVALDQVFENIENSQALVDQSNRILSSAEKGNTG<br>FIIVIILIAVLGSSMILVSVFIIIKKTKKPTGAPPELSGVTNNGFIPHS
>AHV79669.1<br>MSWKVVIIFSLLITPQHGLKESYLEESCSTITEGYLSVLRTGWYTNVFTLEVGDVENLTCADGPSLIKTE<br>LDLTKSALRELKTVSADQLAREEQIENPRQSRFVLGAIALGVATAAAVTAGVAIAKTIRLESEVTAIKNA<br>LKKTNEAVSTLGNGVRVLATAVRELKDFVSKNLTRAINKNKCDIDDLKMAVSFSQFNRRFLNVVRQFSDN<br>AGITPAISLDLMTDAELARAVSNMPTSAGQIKLMLENRAMVRRKGFGILIGVYGSSVIYMVQLPIFGVID<br>TPCWIVKAAPSCSEKKGNYACLLREDQGWYCQNAGSTVYYPNEKDCETRGDHVFCDTAAGINVAEQSKEC<br>NINISTTNYPCKVSTGRHPISMVALSPLGALVACYKGVSCSIGSNRVGIIKQLNKGCSYITNQDADTVTI<br>DNTVYQLSKVEGEQHVIKGRPVSSSFDPVKFPEDQFNVALDQVFENIENSQALVDQSNRILSSAEKGNTG<br>FIIVIILIAVLGSSMILVSVFIIIKKTKKPTGAPPELSGVTNNGFIPHS
>AHV79660.1<br>MSWKVVIIFSLLITPQHGLKESYLEESCSTITEGYLSVLRTGWYTNVFTLEVGDVENLTCADGPSLIKTE<br>LDLTKSALRELKTVSADQLAREEQIENPRQSRFVLGAIALGVATAAAVTAGVAIAKTIRLESEVTAIKNA<br>LKKTNEAVSTLGNGVRVLATAVRELKDFVSKNLTRAINKNKCDIDDLKMAVSFSQFNRRFLNVVRQFSDN<br>AGITPAISLDLMTDAELARAVSNMPTSAGQIKLMLENRAMVRRKGFGILIGVYGSSVIYMVQLPIFGVID<br>TPCWIVKAAPSCSEKKGNYACLLREDQGWYCQNAGSTVYYPNEKDCETRGDHVFCDTAAGINVAEQSKEC<br>NINISTTNYPCKVSTGRHPISMVALSPLGALVACYKGVSCSIGSNRVGIIKQLNKGCSYITNQDADTVTI<br>DNTVYQLSKVEGEQHVIKGRPVSSSFDPVKFPEDQFNVALDQVFENIENSQALVDQSNRILSSAEKGNTG<br>FIIVIILIAVLGSSMILVSVFIIIKKTKKPTGAPPELSGVTNNGFIPHS
>AHV79660.1<br>MSWKVVIIFSLLITPQHGLKESYLEESCSTITEGYLSVLRTGWYTNVFTLEVGDVENLTCADGPSLIKTE<br>LDLTKSALRELKTVSADQLAREEQIENPRQSRFVLGAIALGVATAAAVTAGVAIAKTIRLESEVTAIKNA<br>LKKTNEAVSTLGNGVRVLATAVRELKDFVSKNLTRAINKNKCDIDDLKMAVSFSQFNRRFLNVVRQFSDN<br>AGITPAISLDLMTDAELARAVSNMPTSAGQIKLMLENRAMVRRKGFGILIGVYGSSVIYMVQLPIFGVID<br>TPCWIVKAAPSCSEKKGNYACLLREDQGWYCQNAGSTVYYPNEKDCETRGDHVFCDTAAGINVAEQSKEC<br>NINISTTNYPCKVSTGRHPISMVALSPLGALVACYKGVSCSIGSNRVGIIKQLNKGCSYITNQDADTVTI<br>DNTVYQLSKVEGEQHVIKGRPVSSSFDPVKFPEDQFNVALDQVFENIENSQALVDQSNRILSSAEKGNTG<br>FIIVIILIAVLGSSMILVSVFIIIKKTKKPTGAPPELSGVTNNGFIPHS

>AHV79633.1
MSWKVVIIFSLLITPQHGLKESYLEESCSTITEGYLSVLRTGWYTNVFTLEVGDVENLTCADGPSLIKTE
LDLTKSALRELKTVSADQLAREEQIENPRQSRFVLGAIALGVATAAAVTAGVAIAKTIRLESEVTAIKNA
LKKTNEAVSTLGNGVRVLATAVRELKDFVSKNLTRAINKNKCDIDDLKMAVSFSQFNRRFLNVVRQFSDN
AGITPAISLDLMTDAELARAVSNMPTSAGQIKLMLENRAMVRRKGFGILIGVYGSSVIYMVQLPIFGVID
TPCWIVKAAPSCSEKKGNYACLLREDQGWYCQNAGSTVYYPNEKDCETRGDHVFCDTAAGINVAEQSKEC
NINISTTNYPCKVSTGRHPISMVALSPLGALVACYKGVSCSIGSNRVGIIKQLNKGCSYITNQDADTVTI
DNTVYQLSKVEGEQHVIKGRPVSSSFDPVKFPEDQFNVALDQVFENIENSQALVDQSNRILSSAEKGNTG
FIIVIILIAVLGSSMILVSVFIIIKKTKKPTGAPPELSGVTNNGFIPHS

>AHV79624.1
MSWKVVIIFSLLITPQHGLKESYLEESCSTITEGYLSVLRTGWYTNVFTLEVGDVENLTCSDGPSLIKTE
LDLTKSALRELKTVSADQLAREEQIENPRQSRFVLGAIALGVATAAAVTAGVAIAKTIRLEGEVTAIKNA
LKTTNEAVSTLGNGVRVLATAVRELKDFVSKNLTRAINKNKCDIDDLKMAVSFSQFNRRFLNVVRQFSDN
AGITPAISLDLMTDAELARAVSNMPTSAGQIKLMLENRAMVRRKGFGILIGVYGSSVIYMVQLPIFGVID
TPCWIVKAAPSCSEKKGNYACLLREDQGWYCQNAGSTVYYPNEKDCETRGDHVFCDTAAGINVAEQSKEC
NINISTTNYPCKVSTGRHPISMVALSPLGALVACYKGVSCSIGSNRVGIIKQLNKGCSYITNQDADTVTI
DNTVYQLSKVEGEQHVIKGRPVSSSFDPIKFPEDQFNVALDQVFENIENSQALVDQSNRILSSAEKGNTG
FIIVIILIAVLGSSMILVSIFIIIKKTKKPTGAPPELSGVTNNGFIPHS

>AHV79606.1
MSWKVVVIFSLLITPQHGLKESYLEESCSTITEGYLSVLRTGWYTNVFTLEVGDVENLTCADGPSLIKTE
LDLTKSALRELKTVSADQLAREEQIENPRQSRFVLGAIALGVATAAAVTAGVAIAKTIRLESEVTAIKNA
LKKTNEAVSTLGNGVRVLATAVRELKDFVSKNLTRAINKNKCDIDDLKMAVSFSQFNRRFLNVVRQFSDN
AGITPAISLDLMTDAELARAVSNMPTSAGQIKLMLENRAMVRRKGFGILIGVYGSSVIYMVQLPIFGVID
TPCWIVKAAPSCSEKKGNYACLLREDQGWYCQNAGSTVYYPNEKDCETRGDHVFCDTAAGINVAEQSKEC
NINISTTNYPCKVSTGRHPISMVALSPLGALVACYKGVSCSIGSNRVGIIKQLNKGCSYITNQDADTVTI
DNTVYQLSKVEGEQHVIKGRPVSSSFDPVKFPEDQFNVALDQVFENIENSQALVDQSNRILSSAEKGNTG
FIIVIILIAVLGSSMILVSVFIIIKKTKKPTGAPPELSGVTNNGFIPHS

>AHV79606.1
MSWKVVVIFSLLITPQHGLKESYLEESCSTITEGYLSVLRTGWYTNVFTLEVGDVENLTCADGPSLIKTE
LDLTKSALRELKTVSADQLAREEQIENPRQSRFVLGAIALGVATAAAVTAGVAIAKTIRLESEVTAIKNA
LKKTNEAVSTLGNGVRVLATAVRELKDFVSKNLTRAINKNKCDIDDLKMAVSFSQFNRRFLNVVRQFSDN
AGITPAISLDLMTDAELARAVSNMPTSAGQIKLMLENRAMVRRKGFGILIGVYGSSVIYMVQLPIFGVID
TPCWIVKAAPSCSEKKGNYACLLREDQGWYCQNAGSTVYYPNEKDCETRGDHVFCDTAAGINVAEQSKEC
NINISTTNYPCKVSTGRHPISMVALSPLGALVACYKGVSCSIGSNRVGIIKQLNKGCSYITNQDADTVTI
DNTVYQLSKVEGEQHVIKGRPVSSSFDPVKFPEDQFNVALDQVFENIENSQALVDQSNRILSSAEKGNTG
FIIVIILIAVLGSSMILVSVFIIIKKTKKPTGAPPELSGVTNNGFIPHS

>AHV79590.1
MSWKVVIIFSLLITPQHGLKESYLEESCSTITEGYLSVLRTGWYTNVFTLEVGDVENLTCSDGPSLIKTE
LDLTKSALRELKTVSADQLAREEQIENPRQSRFVLGAIALGVATAAAVTAGVAIAKTIRLEGEVTAIKNA
LKTTNEAVSTLGNGVRVLATAVRELKDFVSKNLTRAINKNKCDIDDLKMAVSFSQFNRRFLNVVRQFSDN
AGITPAISLDLMTDAELARAVSNMPTSAGQIKLMLENRAMVRRKGFGILIGVYGSSVIYMVQLPIFGVID
TPCWIVKAAPSCSEKKGNYACLLREDQGWYCQNAGSTVYYPNEKDCETRGDHVFCDTAAGINVAEQSKEC
NINISTTNYPCKVSTGRHPISMVALSPLGALVACYKGVSCSIGSNRVGIIKQLNKGCSYITNQDADTVTI
DNTVYQLSKVEGEQHVIKGRPVSSSFDPIKFPEDQFNVALDQVFENIENSQALVDQSNRILSSAEKGNTG
FIIVIILIAVLGSSMILVSIFIIIKKTKKPTGAPPELSGVTNNGFIPHS

>AHV79581.1<br>MSWKVVIIFSLLITPQHGLKESYLEESCSTITEGYLSVLRTGWYTNVFTLEVGDVENLTCADGPSLIKTE<br>LDLTKSALRELKTVSADQLAREEQIENPRQSRFVLGAIALGVATAAAVTAGVAIAKTIRLESEVTAIKNA<br>LKKTNEAVSTLGNGVRVLATAVRELKDFVSKNLTRAINKNKCDIDDLKMAVSFSQFNRRFLNVVRQFSDN<br>AGITPAISLDLMTDAELARAVSNMPTSAGQIKLMLENRAMVRRKGFGILIGVYGSSVIYMVQLPIFGVID<br>TPCWIVKAAPSCSEKKGNYACLLREDQGWYCQNAGSTVYYPNEKDCETRGDHVFCDTAAGINVAEQSKEC<br>NINISTTNYPCKVSTGRHPISMVALSPLGALVACYKGVSCSIGSNRVGIIKQLNKGCSYITNQDADTVTI<br>DNTVYQLSKVEGEQHVIKGRPVSSSFDPVKFPEDQFNVALDQVFENIENSQALVDQSNRILSSAEKGNTG<br>FIIVIILIAVLGSSMILVSVFIIIKKTKKPTGAPPELSGVTNNGFIPHS
>AHV79572.1<br>MSWKVVIIFSLLITPQHGLKESYLEESCSTITEGYLSVLRTGWYTNVFTLEVGDVENLTCADGPSLIKTE<br>LDLTKSALRELKTVSADQLAREEQIENPRQSRFVLGAIALGVATAAAVTAGVAIAKTIRLESEVTAIKNA<br>LKKTNEAVSTLGNGVRVLATAVRELKDFVSKNLTRAINKNKCDIDDLKMAVSFSQFNRRFLNVVRQFSDN<br>AGITPAISLDLMTDAELARAVSNMPTSAGQIKLMLENRAMVRRKGFGILIGVYGSSVIYMVQLPIFGVID<br>TPCWIVKAAPSCSEKKGNYACLLREDQGWYCQNAGSTVYYPNEKDCETRGDHVFCDTAAGINVAEQSKEC<br>NINISTTNYPCKVSTGRHPISMVALSPLGALVACYKGVSCSIGSNRVGIIKQLNKGCSYITNQDADTVTI<br>DNTVYQLSKVEGEQHVIKGRPVSSSFDPVKFPEDQFNVALDQVFENIENSQALVDQSNRILSSAEKGNTG<br>FIIVIILIAVLGSSMILVSVFIIIKKTKKPTGAPPELSGVTNNGFIPHS
>AHV79554.1<br>MSWKVVIIFSLLITPQHGLKESYLEESCSTITEGYLSVLRTGWYTNVFTLEVGDVENLTCSDGPSLIKTE<br>LDLTKSALRELKTVSADQLAREEQIENPRQSRFVLGAIALGVATAAAVTAGVAIAKTIRLEGEVTAIKNA<br>LKTTNEAVSTLGNGVRVLATAVRELKDFVSKNLTRAINKNKCDIDDLKMAVSFSQFNRRFLNVVRQFSDN<br>AGITPAISLDLMTDAELARAVSNMPTSAGQIKLMLENRAMVRRKGFGILIGVYGSSVIYMVQLPIFGVID<br>TPCWIVKAAPSCSEKKGNYACLLREDQGWYCQNAGSTVYYPNEKDCETRGDHVFCDTAAGINVAEQSKEC<br>NINISTTNYPCKVSTGRHPISMVALSPLGALVACYKGVSCSIGSNRVGIIKQLNKGCSYITNQDADTVTI<br>DNTVYQLSKVEGEQHVIKGRPVSSSFDPIKFPEDQFNVALDQVFENIENSQALVDQSNRILSSAEKGNTG<br>FIIVIILIAVLGSSMILVSIFIIIKKTKKPTGAPPELSGVTNNGFIPHS
>AHV79545.1<br>MSWKVVVIFSLLITPQHGLKESYLEESCSTITEGYLSVLRTGWYTNVFTLEVGDVENLTCADGPSLIKTE<br>LDLTKSALRELKTVSADQLAREEQIENPRQSRFVLGAIALGVATAAAVTAGVAIAKTIRLESEVTAIKNA<br>LKKTNEAVSTLGNGVRVLATAVRELKDFVSKNLTRAINKNKCDIDDLKMAVSFSQFNRRFLNVVRQFSDN<br>AGITPAISLDLMTDAELARAVSNMPTSAGQIKLMLENRAMVRRKGFGILIGVYGSSVIYMVQLPIFGVID<br>TPCWIVKAAPSCSEKKGNYACLLREDQGWYCQNAGSTVYYPNEKDCETRGDHVFCDTAAGINVAEQSKEC<br>NINISTTNYPCKVSTGRHPISMVALSPLGALVACYKGVSCSIGSNRVGIIKQLNKGCSYITNQDADTVTI<br>DNTVYQLSKVEGEQHVIKGRPVSSSFDPVKFPEDQFNVALDQVFENIENSQALVDQSNRILSSAEKGNTG<br>FIIVIILIAVLGSSMILVSVFIIIKKTKKPTGAPPELSGVTNNGFIPHS
>AHV79545.1<br>MSWKVVVIFSLLITPQHGLKESYLEESCSTITEGYLSVLRTGWYTNVFTLEVGDVENLTCADGPSLIKTE<br>LDLTKSALRELKTVSADQLAREEQIENPRQSRFVLGAIALGVATAAAVTAGVAIAKTIRLESEVTAIKNA<br>LKKTNEAVSTLGNGVRVLATAVRELKDFVSKNLTRAINKNKCDIDDLKMAVSFSQFNRRFLNVVRQFSDN<br>AGITPAISLDLMTDAELARAVSNMPTSAGQIKLMLENRAMVRRKGFGILIGVYGSSVIYMVQLPIFGVID<br>TPCWIVKAAPSCSEKKGNYACLLREDQGWYCQNAGSTVYYPNEKDCETRGDHVFCDTAAGINVAEQSKEC<br>NINISTTNYPCKVSTGRHPISMVALSPLGALVACYKGVSCSIGSNRVGIIKQLNKGCSYITNQDADTVTI<br>DNTVYQLSKVEGEQHVIKGRPVSSSFDPVKFPEDQFNVALDQVFENIENSQALVDQSNRILSSAEKGNTG<br>FIIVIILIAVLGSSMILVSVFIIIKKTKKPTGAPPELSGVTNNGFIPHS

>AHV79527.1<br>MSWKVVIIFSLLITPQHGLKESYLEESCSTITEGYLSVLRTGWYTNVFTLEVGDVENLTCADGPSLIKTE<br>LDLTKSALRELKTVSADQLAREEQIENPRQSRFVLGAIALGVATAAAVTAGVAIAKTIRLESEVTAIKNA<br>LKKTNEAVSTLGNGVRVLATAVRELKDFVSKNLTRAINKNKCDIDDLKMAVSFSQFNRRFLNVVRQFSDN<br>AGITPAISLDLMTDAELARAVSNMPTSAGQIKLMLENRAMVRRKGFGILIGVYGSSVIYMVQLPIFGVID<br>TPCWIVKAAPSCSEKKGNYACLLREDQGWYCQNAGSTVYYPNEKDCETRGDHVFCDTAAGINVAEQSKEC<br>NINISTTNYPCKVSTGRHPISMVALSPLGALVACYKGVSCSIGSNRVGIIKQLNKGCSYITNQDADTVTI<br>DNTVYQLSKVEGEQHVIKGRPVSSSFDPVKFPEDQFNVALDQVFENIENSQALVDQSNRILSSAEKGNTG<br>FIIVIILIAVLGSSMILVSVFIIIKKTKKPTGAPPELSGVTNNGFIPHS
>AHV79518.1<br>MSWKVVIIFSLLITPQHGLKESYLEESCSTITEGYLSVLRTGWYTNVFTLEVGDVENLTCADGPSLIKTE<br>LDLTKSALRELKTVSADQLAREEQIENPRQSRFVLGAIALGVATAAAVTAGVAIAKTIRLESEVTAIKNA<br>LKKTNEAVSTLGNGVRVLATAVRELKDFVSKNLTRAINKNKCDIDDLKMAVSFSQFNRRFLNVVRQFSDN<br>AGITPAISLDLMTDAELARAVSNMPTSAGQIKLMLENRAMVRRKGFGILIGVYGSSVIYMVQLPIFGVID<br>TPCWIVKAAPSCSEKKGNYACLLREDQGWYCQNAGSTVYYPNEKDCETRGDHVFCDTAAGINVAEQSKEC<br>NINISTTNYPCKVSTGRHPISMVALSPLGALVACYKGVSCSIGSNRVGIIKQLNKGCSYITNQDADTVTI<br>DNTVYQLSKVEGEQHVIKGRPVSSSFDPVKFPEDQFNVALDQVFENIENSQALVDQSNRILSSAEKGNTG<br>FIIVIILIAVLGSSMILVSVFIIIKKTKKPTGAPPELSGVTNNGFIPHS
>AHV79509.1
MSWKVVIIFSLLITPQHGLKESYLEESCSTITEGYLSVLRTGWYTNVFTLEVGDVENLTCADGPSLIKTE
LDLTKSALRELKTVSADQLAREEQIENPRQSRFVLGAIALGVATAAAVTAGVAIAKTIRLESEVTAIKNA
LKKTNEAVSTLGNGVRVLATAVRELKDFVSKNLTRAINKNKCDIDDLKMAVSFSQFNRRFLNVVRQFSDN
AGITPAISLDLMTDAELARAVSNMPTSAGQIKLMLENRAMVRRKGFGILIGVYGSSVIYMVQLPIFGVID
TPCWIVKAAPSCSEKKGNYACLLREDQGWYCQNAGSTVYYPNEKDCETRGDHVFCDTAAGINVAEQSKEC
NINISTTNYPCKVSTGRHPISMVALSPLGALVACYKGVSCSIGSNRVGIIKQLNKGCSYITNQDADTVTI
DNTVYQLSKVEGEQHVIKGRPVSSSFDPVKFPEDQFNVALDQVFENIENSQALVDQSNRILSSAEKGNTG
FIIVIILIAVLGSSMILVSVFIIIKKTKKPTGAPPELSGVTNNGFIPHS
>AHV79500.1
MSWKVVIIFSLLITPQHGLKESYLEESCSTITEGYLSVLRTGWYTNVFTLEVGDVENLTCADGPSLIKTE
LDLTKSALRELKTVSADQLAREEQIENPRQSRFVLGAIALGVATAAAVTAGVAIAKTIRLESEVTAIKNA
LKKTNEAVSTLGNGVRVLATAVRELKDFVSKNLTRAINKNKCDIDDLKMAVSFSQFNRRFLNVVRQFSDN
AGITPAISLDLMTDAELARAVSNMPTSAGQIKLMLENRAMVRRKGFGILIGVYGSSVIYMVQLPIFGVID
TPCWIVKAAPSCSEKKGNYACLLREDQGWYCQNAGSTVYYPNEKDCETRGDHVFCDTAAGINVAEQSKEC
NINISTTNYPCKVSTGRHPISMVALSPLGALVACYKGVSCSIGSNRVGIIKQLNKGCSYITNQDADTVTI
DNTVYQLSKVEGEQHVIKGRPVSSSFDPVKFPEDQFNVALDQVFENIENSQALVDQSNRILSSAEKGNTG
FIIVIILIAVLGSSMILVSVFIIIKKTKKPTGAPPELSGVTNNGFIPHS
>AHV79491.1<br>MSWKVMIIISLLITPQHGLKESYLEESCSTITEGYLSVLRTGWYTNVFTLEVGDVENLTCTDGPSLIKTE<br>LDLTKSALRELKTVSADQLAREEQIENPRQSRFVLGAIALGVATAAAVTAGIAIAKTIRLESEVNAIKGA<br>LKQTNEAVSTLGNGVRVLATAVRELKEFVSKNLTSAINRNKCDIADLKMAVSFSQFNRRFLNVVRQFSDN<br>AGITPAISLDLMTDAELARAVSYMPTSAGQIKLMLENRAMVRRKGFGILIGVYGSSVIYMVQLPIFGVID<br>TPCWIIKAAPSCSEKNGNYACLLREDQGWYCKNAGSTVYYPNEKDCETRGDHVFCDTAAGINVAEQSREC<br>NINISTTNYPCKVSTGRHPISMVALSPLGALVACYKGVSCSIGSNRVGIIKQLPKGCSYITNQDADTVTI<br>DNTVYQLSKVEGEQHVIKGRPVSSSFDPIRFPEDQFNVALDQVFESIENSQALVEQSNKILNSAEKGNTG<br>FIIVIILVAVLGLTMISVSIIIIIKKTKKPTGAPPELNGVTNGGFIPHS

>AHV79482.1
MSWKVVIIFSLLITPQHGLKESYLEESCSTITEGYLSVLRTGWYTNVFTLEVGDVENLTCADGPSLIKTE
LDLTKSALRELKTVSADQLAREEQIENPRQSRFVLGAIALGVATAAAVTAGVAIAKTIRLESEVTAIKNA
LKKTNEAVSTLGNGVRVLATAVRELKDFVSKNLTRAINKNKCDIDDLKMAVSFSQFNRRFLNVVRQFSDN
AGITPAISLDLMTDAELARAVSNMPTSAGQIKLMLENRAMVRRKGFGILIGVYGSSVIYMVQLPIFGVID
TPCWIVKAAPSCSEKKGNYACLLREDQGWYCQNAGSTVYYPNEKDCETRGDHVFCDTAAGINVAEQSKEC
NINISTTNYPCKVSTGRHPISMVALSPLGALVACYKGVSCSIGSNRVGIIKQLNKGCSYITNQDADTVTI
DNTVYQLSKVEGEQHVIKGRPVSSSFDPVKFPEDQFNVALDQVFENIENSQALVDQSNRILSSAEKGNTG
FIIVIILIAVLGSSMILVSVFIIIKKTKKPTGAPPELSGVTNNGFIPHS

>AHV79473.1
MSWKVVVIFSLLITPQHGLKESYLEESCSTITEGYLSVLRTGWYTNVFTLEVGDVENLTCADGPSLIKTE
LDLTKSALRELKTVSADQLAREEQIENPRQSRFVLGAIALGVATAAAVTAGVAIAKTIRLESEVTAIKNA
LKKTNEAVSTLGNGVRVLATAVRELKDFVSKNLTRAINKNKCDIDDLKMAVSFSQFNRRFLNVVRQFSDN
AGITPAISLDLMTDAELARAVSNMPTSAGQIKLMLENRAMVRRKGFGILIGVYGSSVIYMVQLPIFGVID
TPCWIVKAAPSCSEKKGNYACLLREDQGWYCQNAGSTVYYPNEKDCETRGDHVFCDTAAGINVAEQSKEC
NINISTTNYPCKVSTGRHPISMVALSPLGALVACYKGVSCSIGSNRVGIIKQLNKGCSYITNQDADTVTI
DNTVYQLSKVEGEQHVIKGRPVSSSFDPVKFPEDQFNVALDQVFENIENSQALVDQSNRILSSAEKGNTG
FIIVIILIAVLGSSMILVSVFIIIKKTKKPTGAPPELSGVTNNGFIPHS

>AHV79464.1
MSWKVVIIFSLLITPQHGLKESYLEESCSTITEGYLSVLRTGWYTNVFTLEVGDVENLTCADGPSLIKTE
LDLTKSALRELKTVSADQLAREEQIENPRQSRFVLGAIALGVATAAAVTAGVAIAKTIRLESEVTAIKNA
LKKTNEAVSTLGNGVRVLATAVRELKDFVSKNLTRAINKNKCDIDDLKMAVSFSQFNRRFLNVVRQFSDN
AGITPAISLDLMTDAELARAVSNMPTSAGQIKLMLENRAMVRRKGFGILIGVYGSSVIYMVQLPIFGVID
TPCWIVKAAPSCSEKKGNYACLLREDQGWYCQNAGSTVYYPNEKDCETRGDHVFCDTAAGINVAEQSKEC
NINISTTNYPCKVSTGRHPISMVALSPLGALVACYKGVSCSIGSNRVGIIKQLNKGCSYITNQDADTVTI
DNTVYQLSKVEGEQHVIKGRPVSSSFDPVKFPEDQFNVALDQVFENIENSQALVDQSNRILSSAEKGNTG
FIIVIILIAVLGSSMILVSVFIIIKKTKKPTGAPPELSGVTNNGFIPHS

>AHV79455.1
MSWKVVIIFSLLITPQHGLKESYLEESCSTITEGYLSVLRTGWYTNVFTLEVGDVENLTCADGPSLIKTE
LDLTKSALRELKTVSADQLAREEQIENPRQSRFVLGAIALGVATAAAVTAGVAIAKTIRLESEVTAIKNA
LKKTNEAVSTLGNGVRVLATAVRELKDFVSKNLTRAINKNKCDINDLKMAVSFSQFNRRFLNVVRQFSDN
AGITPAISLDLMTDAELARAVSNMPTSAGQIKLMLENRAMVRRKGFGILIGVYGSSVIYMVQLPIFGVID
TPCWIVKAAPSCSEKKGNYACLLREDQGWYCQNAGSTVYYPNEKDCETRGDHVFCDTAAGINVAEQSKEC
NINISTTNYPCKVSTGRHPISMVALSPLGALVACYKGVSCSIGSNRVGIIKQLNKGCSYITNQDADTVTI
DNTVYQLSKVEGEQHVIKGRPVSSSFDPVKFPEDQFNVALDQVFENIENSQALVDQSNRILSSAEKGNTG
FIIVIILIAVLGSSMILVSVFIIIKKTKKPTGAPPELSGVTNNGFIPHS

>AHV79446.1
MSWKVVIIFSLLITPQHGLKESYLEESCSTITEGYLSVLRTGWYTNVFTLEVGDVENLTCSDGPSLIKTE
LDLTKSALRELKTVSADQLAREEQIENPRQSRFVLGAIALGVATAAAVTAGVAIAKTIRLEGEVTAIKNA
LKTTNEAVSTLGNGVRVLATAVRELKDFVSKNLTRAINKNKCDIDDLKMAVSFSQFNRRFLNVVRQFSDN
AGITPAISLDLMTDAELARAVSNMPTSAGQIKLMLENRAMVRRKGFGILIGVYGSSVIYMVQLPIFGVID
TPCWIVKAAPSCSEKKGNYACLLREDQGWYCQNAGSTVYYPNEKDCETRGDHVFCDTAAGINVAEQSKEC
NINISTTNYPCKVSTGRHPISMVALSPLGALVACYKGVSCSIGSNRVGIIKQLNKGCSYITNQDADTVTI
DNTVYQLSKVEGEQHVIKGRPVSSSFDPIKFPEDQFNVALDQVFENIENSQALVDQSNRILSSAEKGNTG
FIIVIVLIAVLGSSMILVSIFIIIKKTKKPTGAPPELSGVTNNGFIPHS

>AHV79438.1
MSWKVVIIFSLLITPQHGLKESYLEESCSTITEGYLSVLRTGWYTNVFTLEVGDVENLTCADGPSLIKTE
LDLTKSALRELKTVSADQLAREEQIENPRQSRFVLGAIALGVATAAAVTAGVAIAKTIRLESEVTAIKNA
LKKTNEAVSTLGNGVRVLATAVRELKDFVSKNLTRAINKNKCDIDDLKMAVSFSQFNRRFLNVVRQFSDN
AGITPAISLDLMTDAELARAVSNMPTSAGQIKLMLENRAMVRRKGFGILIGVYGSSVIYMVQLPIFGVID
TPCWIVKAAPSCSEKKGNYACLLREDQGWYCQNAGSTVYYPNEKDCETRGDHVFCDTAAGINVAEQSKEC
NINISTTNYPCKVSTGRHPISMVALSPLGALVACYKGVSCSIGSNRVGIIKQLNKGCSYITNQDADTVTI
DNTVYQLSKVEGEQHVIKGRPVSSSFDPVKFPEDQFNVALDQVFENIENSQALVDQSNRILSSAEKGNTG
FIIVIILIAVLGSSMILVSVFIIIKKTKKPTGAPPELSGVTNNGFIPHS

>AHV79768.1]
MSWKVMIIISLLITPQHGLKESYLEESCSTITEGYLSVLRTGWYTNVFTLEVGDVENLTCTDGPSLIKTE
LDLTKSALRELKTVSADQLAREEQIENPRQSRFVLGAIALGVATAAAVTAGIAIAKTIRLESEVNAIKGA
LKTTNEAVSTLGNGVRVLATAVRELKEFVSKNLTSAINKNKCDIADLKMAVSFSQFNRRFLNVVRQFSDN
AGITPAISLDLMNDAELARAVSYMPTSAGQIKLMLENRAMVRRKGFGILIGVYGSSVIYMVQLPIFGVIN
TPCWIIKAAPSCSEKDGNYACLLREDQGWYCKNAGSTVYYPNEKDCETRGDHVFCDTAAGINVAEQSREC
NINISTTNYPCKVSTGRHPISMVALSPLGALVACYKGVSCSTGSNQVGIIKQLPKGCSYITNQDADTVTI
DNTVYQLSKVEGEQHVIKGRPVSSSFDPIRFPEDQFNVALDQVFESIENSQALVDQSNKILNSAEKGNTG
FIIVIILIAVLGLTMISVSIIIIIKKTRKPTGAPPELNGVTNGGFIPHS

>AHV79678.1
MSWKVVIIFSLLITPQHGLKESYLEESCSTITEGYLSVLRTGWYTNVFTLEVGDVENLTCADGPSLIKTE
LDLTKSALRELKTVSADQLAREEQIENPRQSRFVLGAIALGVATAAAVTAGVAIAKTIRLESEVTAIKNA
LKKTNEAVSTLGNGVRVLATAVRELKDFVSKNLTRAINKNKCDIDDLKMAVSFSQFNRRFLNVVRQFSDN
AGITPAISLDLMTDAELARAVSNMPTSAGQIKLMLENRAMVRRKGFGILIGVYGSSVIYMVQLPIFGVID
TPCWIVKAAPSCSEKKGNYACLLREDQGWYCQNAGSTVYYPNEKDCETRGDHVFCDTAAGINVAEQSKEC
NINISTTNYPCKVSTGRHPISMVALSPLGALVACYKGVSCSIGSNRVGIIKQLNKGCSYITNQDADTVTI
DNTVYQLSKVEGEQHVIKGRPVSSSFDPVKFPEDQFNVALDQVFENIENSQALVDQSNRILSSAEKGNTG
FIIVIILIAVLGSSMILVSVFIIIKKTKKPTGAPPELSGVTNNGFIPHS

>AHV79642.1
MSWKVMIIISLLITPQHGLKESYLEESCSTITEGYLSVLRTGWYTNVFTLEVGDVENLTCTDGPSLIKTE
LDLTKSALRELKTVSADQLAREEQIENPRQSRFVLGAIALGVATAAAVTAGIAIAKTIRLESEVNAIKGA
LKTTNEAVSTLGNGVRVLATAVRELKEFVSKNLTSAINKNKCDIADLKMAVSFSQFNRRFLNVVRQFSDN
AGITPAISLDLMNDAELARAVSYMPTSAGQIKLMLENRAMVRRKGFGILIGVYGSSVIYMVQLPIFGVIN
TPCWIIKAAPSCSEKDGNYACLLREDQGWYCKNAGSTVYYPNEKDCETRGDHVFCDTAAGINVAEQSREC
NINISTTNYPCKVSTGRHPISMVALSPLGALVACYKGVSCSTGSNQVGIIKQLPKGCSYITNQDADTVTI
DNTVYQLSKVEGEQHVIKGRPVSSSFDPIRFPEDQFNVALDQVFESIENSQALVDQSNKILNSAEKGNTG
FIIVIILIAVLGLTMISVSIIIIIKKTRKPTGAPPELNGVTNGGFIPHS

>AHV79615.1
MSWKVMIIISLLITPQHGLKESYLEESCSTITEGYLSVLRTGWYTNVFTLEVGDVENLTCTDGPSLIKTE
LDLTKSALRELKTVSADQLAREEQIENPRQSRFVLGAIALGVATAAAVTAGIAIAKTIRLESEVNAIKGA
LKTTNEAVSTLGNGVRVLATAVRELKEFVSKNLTSAINKNKCDIADLKMAVSFSQFNRRFLNVVRQFSDN
AGITPAISLDLMNDAELARAVSYMPTSAGQIKLMLENRAMVRRKGFGILIGVYGSSVIYMVQLPIFGVIN
TPCWIIKAAPSCSEKDGNYACLLREDQGWYCKNAGSTVYYPNEKDCETRGDHVFCDTAAGINVAEQSREC
NINISTTNYPCKVSTGRHPISMVALSPLGALVACYKGVSCSTGSNQVGIIKQLPKGCSYITNQDADTVTI
DNTVYQLSKVEGEQHVIKGRPVSSSFDPIRFPEDQFNVALDQVFESIENSQALVDQSNKILNSAEKGNTG
FIIVIILIAVLGLTMISVSIIIIIKKTRKPTGAPPELNGVTNGGFIPHS

>AGT75042.1
MSWKVMIIISLLITPQHGLKESYLEESCSTITEGYLSVLRTGWYTNVFTLEVGDVENLTCTDGPSLIKTE
LDLTKSALRELKTVSADQLAREEQIENPRQSRFVLGAIALGVATAAAVTAGIAIAKTIRLESEVNAIKGA
LKQTNEAVSTLGNGVRVLATAVRELKEFVSKNLTSAINRNKCDIADLKMAVSFSQFNRRFLNVVRQFSDN
AGITPAISLDLMTDAELARAVSYMPTSAGQIKLMLENRAMVRRKGFGILIGVYGSSVIYMVQLPIFGVID
TPCWIIKAAPSCSEKNGNYACLLREDQGWYCKNAGSTVYYPNEKDCETRGDHVFCDTAAGINVAEQSREC
NINISTTNYPCKVSTGRHPISMVALSPLGALVACYKGVSCSIGSNRVGIIKQLPKGCSYITNQDADTVTI
DNTVYQLSKVEGEQHVIKGRPVSSSFDPIRFPEDQFNVALDQVFESIENSQALVEQSNKILNSAEKGNTG
FIIVIILVAVLGLTIISVSIIIIIKKTRKPTGAPPELNGVTNGGFIPHS

>AGT75033.1
MSWKVMIIISLLITPQHGLKEGYLEESCSTITEGYLSVLRTGWYTNVFTLEVGDVENLTCTDGPSLIKTE
LDLTKSALRELKTVSADQLAREEQIENPRQSRFVLGAIALGVATAAAVTAGIAIAKTIRLESEVNAIKGA
LKTTNEAVSTLGNGVRVLATAVRELKEFVSKNLTSAINKNKCDIADLKMAVSFSQFNRRFLNVVRQFSDN
AGITPAISLDLMTDAELARAVSYMPTSAGQIKLMLENRAMVRRKGFGILIGVYGSSVIYMVQLPIFGVID
TPCWIIKAAPSCSEKDGNYACLLREDQGWYCKNAGSTVYYPNEKDCETRGDHVFCDTAAGINVAEQSREC
NINISTTNYPCKVSTGRHPISMVALSPLGALVACYKGVSCSIGSNRVGIIKQLPKGCSYITNQDADTVTI
DNTVYQLSKVEGEQHVIKGRPVSSSFDPIKFPEDQFNVALDQVFESIENSQALVDQSNKILNSAEKGNTG
FIIVIILIAVLGLTMISVSIIIIIKKTRKPTGAPPELNGVTNGGFIPHS

>AGT75024.1
MSWKVMIIISLLITPQHGLKESYLEESCSTITEGYLSVLRTGWYTNVFTLEVGDVENLTCTDGPSLIKTE
LDLTKSALRELKTVSADQLAREEQIENPRQSRFVLGAIALGVATAAAVTAGIAIAKTIRLESEVNAIKGA
LKTTNEAVSTLGNGVRVLATAVRELKEFVSKNLTSAINKNKCDIADLKMAVSFSQFNRRFLNVVRQFSDN
AGITPAISLDLMTDAELARAVSYMPTSAGQIKLMLENRAMVRRKGFGILIGVYGSSVIYMVQLPIFGVID
TPCWIIKAAPSCSEKDGNYACLLREDQGWYCKNAGSTVYYPNEKDCETRGDHVFCDTAAGINVAEQSREC
NINISTTNYPCKVSTGRHPISMVALSPLGALVACYKGVSCSIGSNRVGIIKQLPKGCSYITNQDADTVTI
DNTVYQLSKVEGEQHVIKGRPVSSSFDPIKFPEDQFNVALDQVFESIENSQALVDQSNKILNSAEKGNTG
FIIVIILIAVLGLTMISVSIIIIIKKTRKPTGAPPELNGVTNGGFIPHS

>AGT75010.1
MSWKVMIIISLLITPQHGLKESYLEESCSTITEGYLSVLRTGWYTNVFTLEVGDVENLTCTDGPSLIKTE
LDLTKSALRELKTVSADQLAREEQIENPRQSRFVLGAIALGVATAAAVTAGIAIAKTIRLESEVNAIKGA
LKQTNEAVSTLGNGVRVLATAVRELKEFVSKNLTSAINRNKCDIADLKMAVSFSQFNRRFLNVVRQFSDN
AGITPAISLDLMTDAELARAVSYMPTSAGQIKLMLENRAMVRRKGFGILIGVYGSSVIYMVQLPIFGVID
TPCWIIKAAPSCSEKNGNYACLLREDQGWYCKNAGSTVYYPNEKDCETRGDHVFCDTAAGINVAEQSREC
NINISTTNYPCKVSTGRHPISMVALSPLGALVACYKGVSCSIGSNRVGIIKQLPKGCSYITNQDADTVTI
DNTVYQLSKVEGEQHVIKGRPVSSSFDPIRFPEDQFNVALDQVFESIENSQALVEQSNKILNSAEKGNTG
FIIVIILVAVLGLTMISVSIIIIIKKTRKPTGAPPELNGVTNGGFIPHS

>AGT74999.1
MSWKVMIIISLLITPQHGLKESYLEESCSTITEGYLSVLRTGWYTNVFTLEVGDVENLTCTDGPSLIKTE
LDLTKSALRELKTVSADQLAREEQIENPRQSRFVLGAIALGVATAAAVTAGIAIAKTIRLESEVNAIKGA
LKQTNEAVSTLGNGVRVLATAVRELKEFVSKNLTSAINRNKCDIADLKMAVSFSQFNRRFLNVVRQFSDN
AGITPAISLDLMTDAELARAVSYMPTSAGQIKLMLENRAMVRRKGFGILIGVYGSSVIYMVQLPIFGVID
TPCWIIKAAPSCSEKNGNYACLLREDQGWYCKNAGSTVYYPNEKDCETRGDHVFCDTAAGINVAEQSREC
NINISTTNYPCKVSTGRHPISMVALSPLGALVACYKGVSCSIGSNRVGIIKQLPKGCSYITNQDADTVTI
DNTVYQLSKVEGEQHVIKGRPVSSSFDPIRFPEDQFNVALDQVFESIENSQALVEQSNKILNSAEKGNTG
FIIVIILVAVLGLTMISVSIIIIIKKTRKPTGAPPELNGVTNGGFIPHS

>AGT74990.1<br>MSWKVMIIISLLITPQHGLKESYLEESCSTITEGYLSVLRTGWYTNVFTLEVGDVENLTCTDGPSLIKTE<br>LDLTKSALRELKTVSADQLAREEQIENPRQSRFVLGAIALGVATAAAVTAGIAIAKTIRLESEVNAIKGA<br>LKQTNEAVSTLGNGVRVLATAVRELKEFVSKNLTSAINRNKCDIADLKMAVSFSQFNRRFLNVVRQFSDN<br>AGITPAISLDLMTDAELARAVSYMPTSAGQIKLMLENRAMVRRKGFGILIGVYGSSVIYMVQLPIFGVID<br>TPCWIIKAAPSCSEKNGNYACLLREDQGWYCKNAGSTVYYPNEKDCETRGDHVFCDTAAGINVAEQSREC<br>NINISTTNYPCKVSTGRHPISMVALSPLGALVACYKGVSCSIGSNRVGIIKQLPKGCSYITNQDADTVTI<br>DNTVYQLSKVEGEQHVIKGRPVSSSFDPIRFPEDQFNVALDQVFESIENSQALVEQSNKILNSAEKGNTG<br>FIIVIILVAVLGLTMISVSIIIIIKKTRKPTGAPPELNGVTNGGFIPHS
>AGT74981.1<br>MSWKVMIIISLLITPQHGLKESYLEESCSTITEGYLSVLRTGWYTNVFTLEVGDVENLTCTDGPSLIKTE<br>LDLTKSALRELKTVSADQLAREEQIENPRQSRFVLGAIALGVATAAAVTAGIAIAKTIRLESEVNAIKGA<br>LKQTNEAVSTLGNGVRVLATAVRELKEFVSKNLTSAINRNKCDIADLKMAVSFSQFNRRFLNVVRQFSDN<br>AGITPAISLDLMTDAELARAVSYMPTSAGQIKLMLENRAMVRRKGFGILIGVYGSSVIYMVQLPIFGVID<br>TPCWIIKAAPSCSEKNGNYACLLREDQGWYCKNAGSTVYYPNEKDCETRGDHVFCDTAAGINVAEQSREC<br>NINISTTNYPCKVSTGRHPISMVALSPLGALVACYKGVSCSIGSNRVGIIKQLPKGCSYITNQDADTVTI<br>DNTVYQLSKVEGEQHVIKGRPVSSSFDPIRFPEDQFNVALDQVFESIENSQALVEQSNKILNSAEKGNTG<br>FIIVIILVAVLGLTMISVSIIIIIKKTRKPTGAPPELNGVTNGGFIPHS
>AGT74972.1<br>MSWKVMIIISLLITPQHGLKESYLEESCSTITEGYLSVLRTGWYTNVFTLEVGDVENLTCTDGPSLIKTE<br>LDLTKSALRELKTVSADQLAREEQIENPRQSRFVLGAIALGVATAAAVTAGIAIAKTIRLESEVNAIKGA<br>LKQTNEAVSTLGNGVRVLATAVRELKEFVSKNLTSAINKNKCDIADLKMAVSFSQFNRRFLNVVRQFSDN<br>AGITPAISLDLMTDAELARAVSYMPTSAGQIKLMLENRAMVRRKGFGILIGVYGSSVIYMVQLPIFGVID<br>TPCWIIKAAPSCSEKNGNYACLLREDQGWYCKNAGSTVYYPNEKDCETRGDHVFCDTAAGINVAEQSREC<br>NINISTTNYPCKVSTGRHPISMVALSPLGALVACYKGVSCSIGSNRVGIIKQLPKGCSYITNQDADTVTI<br>DNTVYQLSKVEGEQHVIKGRPVSSSFDPIRFPEDQFNVALDQVFESIENSQALVEQSNKILNSAEKGNTG<br>FIIVIILVAVLGLTMISVSIIIIIKKTRKPTGAPPELNGVTNGGFIPHS
>AGT74957.1<br>MSWKVMIIISLLITPQHGLKESYLEESCSTITEGYLSVLRTGWYTNVFTLEVGDVENLTCTDGPSLIKTE<br>LDLTKSALRELKTVSADQLAREEQIENPRQSRFVLGAIALGVATAAAVTAGIAIAKTIRLESEVNAIKGA<br>LKTTNEAVSTLGNGVRVLATAVRELKEFVSKNLTSAINKNKCDIADLKMAVSFSQFNRRFLNVVRQFSDN<br>AGITPAISLDLMTDAELARAVSYMPTSAGQIKLMLENRAMVRRKGFGILIGVYGSSVIYMVQLPIFGVID<br>TPCWIIKAAPSCSEKDGNYACLLREDQGWYCKNAGSTVYYPNEKDCETRGDHVFCDTAAGINVAEQSREC<br>NINISTTNYPCKVSTGRHPISMVALSPLGALVACYKGVSCSIGSNRVGIIKQLPKGCSYITNQDADTVTI<br>DNTVYQLSKVEGEQHVIKGRPVSSSFDPIKFPEDQFNVALDQVFESIENSQALVDQSNKILNSAEKGNTG<br>FIIVIILIAVLGLTMISVSIIIIIKKTRKPTGAPPELNGVTNGGFIPHS
>UZU57849.1<br>MSWKVMIIISLLITPQHGLKESYLEESCSTITEGYHSVLRTGWYTNVFTLEVGDVENLTCTDGPSLIKTE<br>LDLTKSALRELKTVSADQLAREEQIENPRQSRFVLGAIALGVATAAAVTAGIAIAKTIRLESEVNAIKGA<br>LKTTNEAVSTLGNGVRVLATAVRELKEFVSKNLTSAINRNKCDIADLKMAVSFSQFNRRFLNVVRQFSDN<br>AGITPAISLDLMNDAELARAVSYMPTSAGQIKLMLENRAMVRRKGFGILIGVYGSSVIYMVQLPIFGVIN<br>TPCWIIKAAPSCSEKDGNYACLLREDQGWYCKNAGSTVYYPNEKDCETRGDHVFCDTAAGINVAEQSREC<br>NINISTTNYPCKVSTGRHPISMVALSPLGALVACYKGVSCSTGSNQVGIIKQLPKGCSYITNQDADTVTI<br>DNTVYQLSKVEGEQHVIKGRPVSNSFDPIRFPEDQFNVALDQVFESIENSQALVDQSNKILNSAEKGNTG<br>FIIVIILIAVLGLTMISVSIIIIIKKTTRKPAGAPPELNGVTNGGFIPHS

>UZU57843.1<br>MSWKVVIIFSLLITPQHGLKESYLEESCSTITEGYLSVLRTGWYTNVFTLEVGDVENLTCADGPSLIKTE<br>LDLTKSALRELKTVSADQLAREEQIENPRQSRFVLGAIALGVATAAAVTAGVAIAKTIRLESEVTAIKNA<br>LKKTNEAVSTLGNGVRVLATAVRELKDFVSKNLTRAINKNKCDIDDLKMAVSFSQFNRRFLNVVRQFSDN<br>AGITPAISLDLMTDAELARAVSNMPTSAGQIKLMLENRAMVRRKGFGILIGVYGSSVIYMVQLPIFGVID<br>TPCWIVKAAPSCSEKKGNYACLLREDQGWYCQNAGSTVYYPNEKDCETRGDHVFCDTAAGINVAEQSKEC<br>NINISTTNYPCKVSTGRHPISMVALSPLGALVACYKGVSCSIGSNRVGIIKQLNKGCSYITNQDADTVTI<br>DNTVYQLSKVEGEQHVIKGRPVSSSFDPVKFPEDQFNVALDQVFENIENSQALVDQSNRILSSAEKGNTG<br>FIIVIILIAVLGSSMILVSIFIIIKKTKKPTGAPPELSGVTNNGFIPHS
>UZU57837.1<br>MSWKVVIIFSLLITPQHGLKESYLEESCSTITEGYLSVLRTGWYTNVFTLEVGDVENLTCADGPSLIKTE<br>LDLTKSALRELKTVSADQLAREEQIENPRQSRFVLGAIALGVATAAAVTAGVAIAKTIRLESEVTAIKNA<br>LKKTNEAVSTLGNGVRVLATAVRELKDFVSKNLTRAINKNKCDIDDLKMAVSFSQFNRRFLNVVRQFSDN<br>AGITPAISLDLMTDAELARAVSNMPTSAGQIKLMLENRAMVRRKGFGILIGVYGSSVIYMVQLPIFGVID<br>TPCWIVKAAPSCSEKKGNYACLLREDQGWYCQNAGSTVYYPNEKDCETRGDHVFCDTAAGINVAEQSKEC<br>NINISTTNYPCKVSTGRHPISMVALSPLGALVACYKGVSCSIGSNRVGIIKQLNKGCSYITNQDADTVTI<br>DNTVYQLSKVEGEQHVIKGRPVSSSFDPVKFPEDQFNVALDQVFENIENSQALVDQSNRILSSAEKGNTG<br>FIIVIILIAVLGSSMILVSIFIIIKKTRKPTGAPPELSGVTNNGFIPHS
>UZU57831.1<br>MSWKVVIIFSLLITPQHGLKESYLEESCSTITEGYLSVLRTGWYTNVFTLEVGDVENLTCADGPSLIKTE<br>LDLTKSALRELKTVSADQLAREEQIENPRQSRFVLGAIALGVATAAAVTAGVAIAKTIRLESEVTAIKNA<br>LKKTNEAVSTLGNGVRVLATAVRELKDFVSRNLTRAINKNKCDIDDLKMAVSFSQFNRRFLNVVRQFSDN<br>AGITPAISLDLMTDAELARAVSNMPTSAGQIKLMLENRAMVRRKGFGILIGVYGSSVIYMVQLPIFGVID<br>TPCWIVKAAPSCSEKKGNYACLLREDQGWYCQNAGSTVYYPNEKDCETRGDHVFCDTAAGINVAEQSKEC<br>NINISTTNYPCKVSTGRHPISMVALSPLGALVACYKGVSCSIGSNRVGIIKQLNKGCSYITNQDADTVTI<br>DNTVYQLSKVEGEQHVIKGRPVSSSFDPVKFPEDQFNVALDQVFENIENSQALVDQSNRILSSAEKGNTG<br>FIIVIILIAVLGSSMILVSIFIIIKKTRKPTGAPPELSGVTNNGFIPHS
>UZU57825.1<br>MSWKVVIIFSLLITPQHGLKESYLEESCSTITEGYLSVLRTGWYTNVFTLEVGDVENLTCADGPSLIKTE<br>LDLTKSALRELKTVSADQLAREEQIENPRQSRFVLGAIALGVATAAAVTAGVAIAKTIRLESEVTAIKNA<br>LKKTNEAVSTLGNGVRVLATAVRELKDFVSKNLTRAINKNKCDIDDLKMAVSFSQFNRRFLNVVRQFSDN<br>AGITPAISLDLMTDAELARAVSNMPTSAGQIKLMLENRAMVRRKGFGILIGVYGSSVIYMVQLPIFGVID<br>TPCWIVKAAPSCSEKKGNYACLLREDQGWYCQNAGSTVYYPNEKDCETRGDHVFCDTAAGINVAEQSKEC<br>NINISTTNYPCKVSTGRHPISMVALSPLGALVACYKGVSCSIGSNRVGIIKQLNKGCSYITNQDADTVTI<br>DNTVYQLSKVEGEQHVIKGRPVSSSFDPVKFPEDQFNVALDQVFENIENSQALVDQSNRILSSAEKGNTG<br>FIIVIILIAVLGSSMILVSIFIIIKKTRKPTGAPPELSGVTNNGFIPHS
>UZU57819.1<br>MSWKVVIIFSLLITPQHGLKESYLEESCSTITEGYLSVLRTGWYTNVFTLEVGDVENLTCADGPSLIKTE<br>LDLTKSALRELKTVSADQLAREEQIENPRQSRFVLGAIALGVATAAAVTAGVAIAKTIRLESEVTAIKNA<br>LKKTNEAVSTLGNGVRVLATAVRELKDFVSKNLTRAINKNKCDIDDLKMAVSFSQFNRRFLNVVRQFSDN<br>AGITPAISLDLMTDAELARAVSNMPTSAGQIKLMLENRAMVRRKGFGILIGVYGSSVIYMVQLPIFGVID<br>TPCWIVKAAPSCSEKKGNYACLLREDQGWYCQNAGSTVYYPNEKDCETRGDHVFCDTAAGINVAEQSKEC<br>NINISTTNYPCKVSTGRHPISMVALSPLGALVACYKGVSCSIGSNRVGIIKQLNKGCSYITNQDADTVTI<br>DNTVYQLSKVEGEQHVIKGRPVSSSFDPVKFPEDQFNVALDQVFENIENSQALVDQSNRILSSAEKGNTG<br>FIIVIILIAVLGSSMILVSIFIIIKKTRKPTGAPPELSGVTNNGFIPHS

>UZU57813.1
MSWKVVIIFSLLITPQHGLKESYLEESCSTITEGYLSVLRTGWYTNVFTLEVGDVENLTCADGPSLIKTE
LDLTKSALRELKTVSADQLAREEQIENPRQSRFVLGAIALGVATAAAVTAGVAIAKTIRLESEVTAIKNA
LKKTNEAVSTLGNGVRVLATAVRELKDFVSKNLTRAINKNKCDIDDLKMAVSFSQFNRRFLNVVRQFSDN
AGITPAISLDLMTDAELARAVSNMPTSAGQIKLMLENRAMVRRKGFGILIGVYGSSVIYMVQLPIFGVID
TPCWIVKAAPSCSEKKGNYACLLREDQGWYCQNAGSTVYYPNEKDCETRGDHVFCDTAAGINVAEQSKEC
NINISTTNYPCKVSTGRHPISMVALSPLGALVACYKGVSCSIGSNRVGIIKQLNKGCSYITNQDADTVTI
DNTVYQLSKVEGEQHVIKGRPVSSSFDPVKFPEDQFNVALDQVFENIENSQALVDQSNRILSSAEKGNTG
FIIVIILIAVLGSSMILVSILIIIKKTRKPTGAPPELSGVTNNGFIPHS

>UZU57807.1
MSWKVVIIFSLLITPQHGLKESYLEESCSTITEGYLSVLRTGWYTNVFTLEVGDVENLTCADGPSLIKTE
LDLTKSALRELKTVSADQLAREEQIENPRQSRFVLGAIALGVATAAAVTAGVAIAKTIRLESEVTAIKNA
LKKTNEAVSTLGNGVRVLATAVRELKDFVSKNLTRAINKNKCDIDDLKMAVSFSQFNRRFLNVVRQFSDN
AGITPAISLDLMTDAELARAVSNMPTSAGQIKLMLENRAMVRRKGFGILIGVYGSSVIYMVQLPIFGVID
TPCWIVKAAPSCSEKKGNYACLLREDQGWYCQNAGSTVYYPNEKDCETRGDHVFCDTAAGINVAEQSKEC
NINISTTNYPCKVSTGRHPISMVALSPLGALVACYKGVSCSIGSNRVGIIKQLNKGCSYITNQDADTVTI
DNTVYQLSKVEGEQHVIKGRPVSSSFDPVKFPEDQFNVALDQVFENIENSQALVDQSNRILSSAEKGNTG
FIIVIILIAVLGSSMILVSIFIIIKKTKKPTGAPPELSGVTNNGFIPHS

>UZU57801.1
MSWKVVIIFSLLITPQHGLKESYLEESCSTITEGYLSVLRTGWYTNVFTLEVGDVENLTCADGPSLIKTE
LDLTKSALRELKTVSADQLAREEQIENPRQSRFVLGAIALGVATAAAVTAGVAIAKTIRLESEVTAIKNA
LKKTNEAVSTLGNGVRVLATAVRELKDFVSKNLTRAINKNKCDIDDLKMAVSFSQFNRRFLNVVRQFSDN
AGITPAISLDLMTDAELARAVSNMPTSAGQIKLMLENRAMVRRKGFGILIGVYGSSVIYMVQLPIFGVID
TPCWIVKAAPSCSEKKGNYACLLREDQGWYCQNAGSTVYYPNEKDCETRGDHVFCDTAAGINVAEQSKEC
NINISTTNYPCKVSTGRHPISMVALSPLGALVACYKGVSCSIGSNRVGIIKQLNKGCSYITNQDADTVTI
DNTVYQLSKVEGEQHVIKGRPVSSSFDPVKFPEDQFNVALDQVFENIENSQALVDQSNRILSSAEKGNTG
FIIVIILIAVLGSSMILVSIFIIIKKTRKPTGAPPELSGVTNNGFIPHS

>UZU57795.1
MSWKVVIIFSLLITPQHGLKESYLEESCSTITEGYLSVLRTGWYTNVFTLEVGDVENLTCADGPSLIKTE
LDLTKSALRELKTVSADQLAREEQIENPRQSRFVLGAIALGVATAAAVTAGVAIAKTIRLESEVTAIKNA
LKKTNEAVSTLGNGVRVLATAVRELKDFVSRNLTRAINKNKCDIDDLKMAVSFSQFNRRFLNVVRQFSDN
AGITPAISLDLMTDAELARAVSNMPTSAGQIKLMLENRAMVRRKGFGILIGVYGSSVIYMVQLPIFGVID
TPCWIVKAAPSCSEKKGNYACLLREDQGWYCQNAGSTVYYPNEKDCETRGDHVFCDTAAGINVAEQSKEC
NINISTTNYPCKVSTGRHPISMVALSPLGALVACYKGVSCSIGSNRVGIIKQLNKGCSYITNQDADTVTI
DNTVYQLSKVEGEQHVIKGRPVSSSFDPVKFPEDQFNVALDQVFENIENSQALVDQSNRILSSAEKGNTG
FIIVIILIAVLGSSMILVSIFIIIKKTRKPTGAPPELSGVTNNGFIPHS

>UZU57789.1
MSWKVVIIFSLLITPQHGLKESYLEESCSTITEGYLSVLRTGWYTNVFTLEVGDVENLTCADGPSLIKTE
LDLTKSALRELKTVSADQLAREEQIENPRQSRFVLGAIALGVATAAAVTAGVAIAKTIRLESEVTAIKNA
LKKTNEAVSTLGNGVRVLATAVRELKDFVSKNLTRAINKNKCDIDDLKMAVSFSQFNRRFLNVVRQFSDN
AGITPAISLDLMTDAELARAVSNMPTSAGQIKLMLENRAMVRRKGFGILIGVYGSSVIYMVQLPIFGVID
TPCWIVKAAPSCSEKKGNYACLLREDQGWYCQNAGSTVYYPNEKDCETRGDHVFCDTAAGINVAEQSKEC
NINISTTNYPCKVSTGRHPISMVALSPLGALVACYKGVSCSIGSNRVGIIKQLNKGCSYITNQDADTVTI
DNTVYQLSKVEGEQHVIKGRPVSSSFDPVKFPEDQFNVALDQVFENIENSQALVDQSNRILSSAEKGNTG
FIIVIILIAVLGSSMILVSIFIIIKKTRKPTGAPPELSGVTNNGFIPHS

>UZU57783.1<br>MSWKVVIIFSLLITPQHGLKESYLEESCSTITEGYLSVLRTGWYTNVFTLEVGDVENLTCADGPSLIKTE<br>LDLTKSALRELKTVSADQLAREEQIENPRQSRFVLGAIALGVATAAAVTAGVAIAKTIRLESEVTAIKNA<br>LKKTNEAVSTLGNGVRVLATAVRELKDFVSKNLTRAINKNKCDIDDLKMAVSFSQFNRRFLNVVRQFSDN<br>AGITPAISLDLMTDAELARAVSNMPTSAGQIKLMLENRAMVRRKGFGILIGVYGSSVIYMVQLPIFGVID<br>TPCWIVKAAPSCSEKKGNYACLLREDQGWYCQNAGSTVYYPNEKDCETRGDHVFCDTAAGINVAEQSKEC<br>NINISTTNYPCKVSTGRHPISMVALSPLGALVACYKGVSCSIGSNRVGIIKQLNKGCSYITNQDADTVTI<br>DNTVYQLSKVEGEQHVIKGRPVSSSFDPVKFPEDQFNVALDQVFENIENSQALVDQSNRILSSAEKGNTG<br>FIIVTILIAVLGSSMILVSIFIIIKKTRKPTGAPPELSGVTNNGFIPHS
>UZU57777.1<br>MSWKVVIIFSLLITPQHGLKESYLEESCSTITEGYLSVLRTGWYTNVFTLEVGDVENLTCADGPSLIKTE<br>LDLTKSALRELKTVSADQLAREEQIENPRQSRFVLGAIALGVATAAAVTAGVAIAKTIRLESEVTAIKNA<br>LKKTNEAVSTLGNGVRVLATAVRELKDFVSKNLTRAINKNKCDIDDLKMAVSFSQFNRRFLNVVRQFSDN<br>AGITPAISLDLMTDAELARAVSNMPTSAGQIKLMLENRAMVRRKGFGILIGVYGSSVIYMVQLPIFGVID<br>TPCWIVKAAPSCSEKKGNYACLLREDQGWYCQNAGSTVYYPNEKDCETRGDHVFCDTAAGINVAEQSKEC<br>NINISTTNYPCKVSTGRHPISMVALSPLGALVACYKGVSCSIGSNRVGIIKQLNKGCSYITNQDADTVTI<br>DNTVYQLSKVEGEQHVIKGRPVSSSFDPVKFPEDQFNVALDQVFENIENSQALVDQSNRILSSAEKGNTG<br>FIIVIILIAVLGSSMILVSIFIIIKKTRKPTGAPPELSGVTNNGFIPHS
>UZU57771.1<br>MSWKVVIIFSLLITPQHGLKESYLEESCSTITEGYLSVLRTGWYTNVFTLEVGDVENLTCADGPSLIKTE<br>LDLTKSALRELKTVSADQLAREEQIENPRQSRFVLGAIALGVATAAAVTAGVAIAKTIRLESEVTAIKNA<br>LKKTNEAVSTLGNGVRVLATAVRELKDFVSKNLTRAINKNKCDIDDLKMAVSFSQFNRRFLNVVRQFSDN<br>AGITPAISLDLMTDAELARAVSNMPTSAGQIKLMLENRAMVRRKGFGILIGVYGSSVIYMVQLPIFGVID<br>TPCWIVKAAPSCSEKKGNYACLLREDQGWYCQNAGSTVYYPNEKDCETRGDHVFCDTAAGINVAEQSKEC<br>NINISTTNYPCKVSTGRHPISMVALSPLGALVACYKGVSCSIGSNRVGIIKQLNKGCSYITNQDADTVTI<br>DNTVYQLSKVEGEQHVIKGRPVSSSFDPVKFPEDQFNVALDQVFENIENSQALVDQSNRILSSAEKGNTG<br>FIIVIILIAVLGSSMILVSIFIIIKKTKKPTGAPPELSGVTNNGFIPHS

Table S3. *Influenza virus* neuraminidase protein

>NP_040981.1<br>MNPNQKIITIGSICLVVGLISLILQIGNIISIWISHSIQTGSQNHTGICNQNIITYKNSTWVKDTTSVIL<br>TGNSSLCPIRGWAIYSKDNSIRIGSKGDVFVIREPFISCSHLECRTFFLTQGALLNDRHSNGTVKDRSPY<br>RALMSCPVGEAPSPYNSRFESVAWSASACHDGMGWLTIGISGPDNGAVAVLKYNGIITETIKSWRKKILR<br>TQESECACVNGSCFTIMTDGPSDGLASYKIFKIEKGKVTKSIELNAPNSHYEECSCYPDTGKVMCVCRDN<br>WHGSNRPWVSFDQNLDYQIGYICSGVFGDNPRPKDGTGSCGPVYVDGANGVKGFSYRYGNGVWIGRTKSH<br>SSRHGFEMIWDPNGWTETDSKFSVRQDVVAMTDWSGYSGSFVQHPELTGLDCIRPCFWVELIRGRPKEKT<br>IWTSASSISFCGVNSDTVDWSWPDGAELPFTIDK
>ABP64723.1<br>MNPNQKIITIGSICMVVGLISLILQIGNIISIWISHSIQTGSQNHTGICNQNIITYKNSTWVKDTTSVIL<br>TGNSSLCPIRGWAIYSKDNSIRIGSKGDVFVIREPFISCSHLECRTFFLTQGALLNDRHSNGTVKDRSPY<br>RALMSCPVGEAPSPYNSRFESVAWSASACHDGMGWLTIGISGPDNGAVAVLKYNGIITETIKSWRKKILR<br>TQESECACVNGSCFTIMTDGPSDGLASYKIFKIEKGKVTKSIELNAPNSHYEECSCYPDTGKVMCVCRDN<br>WHGSNRPWVSFDQNLDYQIGYICSGVFGDNPRPKDGTGSCGPVYVDGANGVKGFSYRYGNGVWIGRTKSH<br>SSRHGFEMIWDPNGWTETDSEFSVRQDVVAMTDWSGYSGSFVQHPELTGLDCIRPCFWVELIRGRPKEKT<br>IWTSASSISFCGVNSDTVDWSWPDGAELPFTIDK
>AGO00364.1<br>MNPNQKIITIGSICMVVGLISLILQIGNIISIWISHSIQTGSQNHTGICNQNIITYKNSTWVKDTTSVIL<br>TGNSSLCPIRGWAIYSKDNSIRIGSKGDVFVIREPFISCSHLECRTFFLTQGALLNDRHSNGTVKDRSPY<br>RALMSCPVGEAPSPYNSRFESVAWSASACHDGMGWLTIGISGPDNGAVAVLKYNGIITETIKSWRKKILR<br>TQESECACVNGSCFTIMTDGPSDGLASYKIFKIEKGKVTKSIELNAPNSHYEECSCYPDTGKVMCVCRDN<br>WHGSNRPWVSFDQNLDYQIGYICSGVFGDNPRPKDGTGSCGPVYVDGANGVKGFSYRYGNGVWIGRTKSH<br>SSRHGFEMIWDPNGWTETDSKFSVRQDVVAMTDWSGYSGSFVQHPELTGLDCIRPCFWVELIRGRPKEKT<br>IWTSASSISFCGVNSDTVNWSWPDGAELPFTIDK
>ABD77678.1<br>MNPNQKIITIGSICLVVGLISLILQIGNIISIWISHSIQTGSQNHTGICNQNIITYKNSTWVKDTTSVIL<br>TGNSSLCPIRGWAIYSKDNSIRIGSKGDVFVIREPFISCSHLECRTFFLTQGALLNDKHSSGTVKDRSPY<br>RALMSCPVGEAPSPYNSRFESVAWSASACHDGMGWLTIGISGPDNGAVAVLKYNGIITETIKSWRKKILR<br>TQESECACVNGSCFTIMTDGPSDGLASYKIFKIEKGKVTKSIELNAPNSHYEECSCYPDTGKVMCVCRDN<br>WHGSNRPWVSFDQNLDYQIGYICSGVFGDNPRPEDGTGSCGPVYVDGANGVKGFSYRYGNGVWIGRTKSH<br>SSRHGFEMIWDPNGWTETDSKFSVRQDVVAMTDWSGYSGSFVQHPELTGLDCMRPCFWVELIRGRPKEKT<br>IWTSASSISFCGVNSDTVDWSWPDGAELPFSIDK

>AAM75160.1
MNPNQKITTIGSICLVVGLISLILQIGNIISIWISHSIQTGSQNHTGICNQNIITYKNSTWVKDTTSVIL
TGNSSLCPIRGWAIYSKDNSIRIGSKGDVFVIREPFISCSHLECRTFFLTQGALLNDKHSNGTVKDRSPY
RALMSCPVGEAPSPYNSRFESVAWSASACHDGMGWLTIGISGPDNGAVAVLKYNGIITETIKSWRKKILR
TQESECACVNGSCFTIMTDGPSDGLASYKIFKIEKGKVTKSIELNAPNSHYEECSCYPDTGKVMCVCRDN
WHGSNRPWVSFDQNLDYQIGYICSGVFGDNPRPEDGTGSCGPVYVDGANGVKGFSYRYGNGVWIGRTKSH
SSRHGFEMIWDPNGWTETDSKFSVRQDVVAMTDWSGYSGSFVQHPELTGLDCMRPCFWVELIRGRPKEKT
IWTSASSISFCGVNSDTVDWSWPDGAELPFSIDK

>ADX99945.1
MNPNQKIITIGSICLVVGLISLILQIGNIISIWISHSIQTGSQNHTGICNQNIITYKNSTWVKDTTSVIL
TGNSSLCPIRGWAIYSKDNSIRIGSKGDVFVIREPFISCSHLECRTFFLTQGALLNDKHSSGTVKDRSPY
RALMSCPVGEAPSPYNSRFESVAWSASACHDGMGWLTIGISGPDNGAVAVLKYNGIITETIKSWRKKILR
TQESECACVNGSCFTIMTDGPSDGLASYRIFKIEKGKVTKSIELNAPNSHYEECSCYPDTGKVMCVCRDN
WHGSNRPWVSFDQNLDYQIGYICSGVFGDNPRPEDGTGSCGPVYVDGANGVKGFSYRYGNGVWIGRTKSH
SSRHGFEMIWDPNGWTETDSKFSVRQDVVAMTDWSGYSGSFVQHPELTGLDCMRPCFWVELIRGRPKEKT
IWTSASSISFCGVNSDTVDWSWPDGAELPFSIDK

>AEX96926.1
MNPNQKIITIGSICLVVGLISLILQIGNIISIWISHSIQTGSQNHTGICNQNIITYKNSTWIKDTTSVIL
TGNSSLCPIRGWAIYSKDNSIRIGSKGDVFVIREPFISCSHLECRTFFLTQGALLNDKHSSGTVKDRSPY
RALMSCPVGEAPSPYNSRFESVAWSASACHDGMGWLTIGISGPDNGAVAVLKYNGIITETIKSWRKKILR
TQESECACVNGSCFTIMTDGPSDGLASYKIFKIEKGKVTKSIELNAPNSHYEECSCYPDTGKVMCVCRDN
WHGSNRPWVSFDQNLDYQIGYICSGVFGDNPRPEDGTGSCGPVYVDGANGVKGFSYRYGNGVWIGRTKSH
SSRHGFEMIWDPNGWTETDSKFSVRQDVVAMTDWSGYSGSFVQHPELTGLDCMRPCFWVELIRGRPKEKT
IWTSASSISFCGVNSDTVDWSWPDGAELPFSIDK

>BAV59613.1
MNPNQKIITIGSICLVVGLISLILQIGNIISIWISHSIQTGSQNHTGICNQNIITYKNSTWVKDTTSVIL
TGNSSLCPIRGWAIYSKDNSIRIGSKGDVFVIREPFISCSHLECRTFFLTQGALLNDKHSSGTVKDRSPY
RALMSCPVGEAPSPYNSRFESVAWSASACHDGMGWLTIGISGPDNGAVAVLKYNGIITETIKSWRKKILR
TQESECACVNGSCFTIMTDGPSDGLASYKIFKIEKGRVTKSIELNAPNSHYEECSCYPDTGKVMCVCRDN
WHGSNRPWVSFDQNLDYQIGYICSGVFGDNPRPEDGTGSCGPVYVDGANGVKGFSYRYGNGVWIGRTKSH
SSRHGFEMIWDPNGWTETDSKFSVRQDVVAMTDWSGYSGSFVQHPELTGLDCMRPCFWVELIRGRPKEKT
IWTSASSISFCGVNSDTVDWSWPDGAELPFSIDK

>ADX99912.1
MNPNQKIITIGSICLVVGLISLILQIGNIISIWISHSIQTGSQNHTGICNQNIITYKNSTWVKDTTSVIL
TGNSSLCPIRGWAIYSKDNSIRIGSKGDVFIIREPFISCSHLECRTFFLTQGALLNDKHSSGTVKDRSPY
RALMSCPVGEAPSPYNSRFESVAWSASACHDGMGWLTIGISGPDNGAVAVLKYNGIITETIKSWRKKILR
TQESECACVNGSCFTIMTDGPSDGLASYKIFKIEKGKVTKSIELNAPNSHYEECSCYPDTGKVMCVCRDN
WHGSNRPWVSFDQNLDYQIGYICSGVFGDNPRPEDGTGSCGPVYVDGANGVKGFSYRYGNGVWIGRTKSH
SSRHGFEMIWDPNGWTETDSKFSVRQDVVAMTDWSGYSGSFVQHPELTGLDCMRPCFWVELIRGRPKEKT
IWTSASSISFCGVNSDTVDWSWPDGAELPFSIDK

>ACR15351.1

MNPNQKIITIGSICLVVGLISLILQIGNIISIWISHSIQTGSQNHTGICNQNIITYKNSTWIKDTTSVIL
TGNSSLCPIRGWAIYSKDNSIRIGSKGDVFVIREPFISCSHLECRTFFLTQGALLNDKHSSGTVKDRSPY
RALMSCPVGEAPSPYNSRFESVAWSASACHDGMGWLTIGISGPDNGAVAVLKYNGIITETIKSWRKKILR
TQESECACVNGSCFTIMTDGPSDGLASYKIFKIEKGKVTKSIELNAPNSHYEECSCYPDTGKVMCVCRDN
WHGSNRPWVSFDQNLDYQIGYICSGVFGDNPRPEDGTGSCGPVYVDGANGVKGFSYRYGNGVWIGRTKSH
SSRHGFEMIWDPNGWTETDSKFSVRQDVVAMADWSGYSGSFVQHPELTGLDCMRPCFWVELIRGRPKEKT
IWTSASSISFCGVNSDTVDWSWPDGAELPFSIDK

>AGQ47993.1

MNPNQKIITIGSICLVVGLISLILQIGNIISIWISHSIQTGSQNHTGICNQNIITYKNSTWVKDTTSVIL
TGNSSLCPIRGWAIYSKDNSIRIGSKGDVFVIREPFISCSHLECRTFFLTQGALLNDKHSSGTVKDRSPY
RALMSCPVGEAPSPYNSRFESVAWSASACHDGMGWLTIGISGPDNGAVAVLKYNGIITETIKSWRKKILR
TQESECACVNGSCFTIMTDGPSDGLASYKIFKIEKGKVTKSIELNAPNSHYEECSCYPDTGKVMCVCRDN
WHGSNRPWVSFDQNLDYQIGYICSGVFGDNPRPEDGTGSCGPVYVDGANGVKGFSYRYSNGVWIGRTKSH
SSRHGFEMIWDPNGWTETDSKFSVRQDVVAMTDWSGYSGSFVQHPELTGLDCMRPCFWVELIRGRPKEKT
IWTSASSISFCGVNSDTVDWSWPDGAELPFSIDK

>AVY92599.1

MNPNQKIITIGSICLVVGLISLILQIGNIISIWISHSIQTGSQNHTGICNQNIITYKNSTWVKDTTSVIL
TGNSSLCPIRGWAIYSKDNSIRIGSKGDVFVIREPFISCSHLECRTFFLTQGALLNDKHSSGTVKDRSPY
RALMSCPVGEAPSPYNSRFESVAWSASACHDGMGWLTIGISGPDNGAVAVLKYNSIITETIKSWRKKILR
TQESECACVNGSCFTIMTDGPSDGLASYKIFKIEKGKVTKSIELNAPNSHYEECSCYPDTGKVMCVCRDN
WHGSNRPWVSFDQNLDYQIGYICSGVFGDNPRPEDGTGSCGPVYVDGANGVKGFSYRYGNGVWIGRTKSH
SSRHGFEMIWDPNGWTETDSKFSVRQDVVAMTDWSGYSGSFVQHPELTGLDCMRPCFWVELIRGRPKEKT
IWTSASSISFCGVNSDTVDWSWPDGAELPFSIDK

>ACV49548.1

MNPNQKIITIGSICLVVGLISLILQIGNIISIWISHSIQTGSQNHTGICNQNIITYKNSTWVKDTTSVIL
TGNSSLCPIRGWAIYSKDNSIRIGSKGDVFVIREPFISCSHLECRTFFLTQGALLNDKHSSGTVKDRSPY
RALMSCPVGEAPSPYNSRFESVAWSASACHDGMGWLTIGISGPDNGAVAVLKYNGIITETIKSWRKKILR
TQESECACVNGSCFTIMTDGPSDGLASYKIFKIEKGKVTKSIELNAPNSHYEECSCYPDTGKVMCVCRDN
WHGSNRPWVSFDQNLDYQIGYICSGVFGDNPRPEDGTGSCGPVYVDGANGVKGFSYRYGNGVWIGRTKSH
SSRHGFEMIWDPNGWTETDSKFSVRQDVVAMTDWSGYSGSFVQHPELTGLDCMRPCFWVELIRGRPKEGT
IWTSASSISFCGVNSDTVDWSWPDGAELPFSIDK

>ADX99661.1

MNPNQKIITIGSICLVVGLISLILQIGNIISIWISHSIQTGSQNHTGICNQNIITYKNSTWVKDTTSVIL
TGNSSLCPIRGWAIYSKDNSIRIGSKGDVFVIREPFISCSHLECRTFFLTQGALLNDKHSSGTVKDRSPY
RALMSCPVGEAPSPYNSRFESVAWSASACHDGMGWLTIGISGPDNGAVAVLKYNGIITETIKSWRKKILR
TQESECACVNGSCFTIMTDGPSDGLASYKIFKIEKGKVTKSIELNAPNSNYEECSCYPDTGKVMCVCRDN
WHGSNRPWVSFDQNLDYQIGYICSGVFGDNPRPEDGTGSCGPVYVDGANGVKGFSYRYGNGVWIGRTKSH
SSRHGFEMIWDPNGWTETDSKFSVRQDVVAMTDWSGYSGSFVQHPELTGLDCMRPCFWVELIRGRPKEKT
IWTSASSISFCGVNSDTVDWSWPDGAELPFSIDK

>QGJ97648.1<br>MNPNQKIITIGSICLVVGLISLILQIGNIISIWISHSIQTGSQNHTGICNQNIITYKNSTWVKDTTSVIL<br>TGNSSLCPIRGWAIYSKDNSIRIGSKGDVFVIREPFISCSHLECRTFFLTQGALLNDKHSSGTVKDRSPY<br>RALMSCPVGEAPSPYNSRFESVAWSASACHDGMGWLTIGISGPDNGAVAVLKYNGIITETIKSWRKKILR<br>TQESECACVNGSCFTIMTDGPSDGLASYKIFKIEKGKVTKSIELNAPNSHYEECSCYPDTGKVMCVCRDN<br>WHGSNRPWVSFDQNLDYQIGYICSGVFGDNPRPEDGTGSCGPVYVDGANGVKGFSYRYGNGVWIGRTKGH<br>SSRHGFEMIWDPNGWTETDSKFSVRQDVVAMTDWSGYSGSFVQHPELTGLDCMRPCFWVELIRGRPKEKT<br>IWTSASSISFCGVNSDTVDWSWPDGAELPFSIDK
>UKC63131.1<br>MNPNQKIITIGSICLVVGLISLILQIGNIISIWISHSIQTGSQNHTGICNQNIITYKNSTWVKGTTSVIL<br>TGNSSLCPIRGWAIYSKDNSIRIGSKGDVFVIREPFISCSHLECRTFFLTQGALLNDKHSSGTVKDRSPY<br>RALMSCPVGEAPSPYNSRFESVAWSASACHDGMGWLTIGISGPDNGAVAVLKYNGIITETIKSWRKKILR<br>TQESECACVNGSCFTIMTDGPSDGLASYKIFKIEKGKVTKSIELNAPNSHYEECSCYPDTGKVMCVCRDN<br>WHGSNRPWVSFDQNLDYQIGYICSGVFGDNPRPEDGTGSCGPVYVDGANGVKGFSYRYGNGVWIGRTKGH<br>SSRHGFEMIWDPNGWTETDSKFSVRQDVVAMTDWSGYSGSFVQHPELTGLDCMRPCFWVELIRGRPKEKT<br>IWTSASSISFCGVNSDTVDWSWPDGAELPFSIDK
>AYA81845.1<br>MNPNQKIITIGSICMVVGLINLLLQIGNIISIWISHSIQTGSQNHTGICNQNIITYKNSTWVKDTTSVIL<br>TGNSSLCPIRGWAIYSKDNTIRIGSKGDVFVIREPFISCSHLECRTFFLTQGALLNDKHSNGTVKDRSPY<br>RALMSCPVGEAPSPYNSRFESVAWSASACHDGMGWLTIGISGPDNGAVAVLKYNGIITETIKSWRKKILR<br>TQESECACVNGSCFTIMTDGPSDGLASYKIFKIEKGKVTKSIELNARNFHYEECSCYPDTGKVMCVCRDN<br>WHGSNRPWVSFDQNLDYQIGYICSGVFGDNPRPKDGTGSCGPVYVDGANGVKGFSYRYGNGVWIGRTKSH<br>SSRHGFEMIWDPNGWTETDSKFSVRQDVVAMTDWSGYSGSFVQHPELTGLDCIRPCFWVELIRGRPKENT<br>IWTSASSISFCGVNSDTVDWSWPDGAELPFTIDK
>ACL12143.1<br>SNQKIITIGSICLVVGLISLILQIGNIISIWISHSIQTGSQNHTGICNQNIITYKNSTWVKDTTSVILTG<br>NSSLCPIRGWAIYSKDNSIRIGSKGDVFVIREPFISCSHLECRTFFLTQGALLNDKHSSGTVKDRSPYRA<br>LMSCPVGEAPSPYNSRFESVAWSASACHDGMGWLTIGISGPDNGAVAVLKYNGIITETIKSWRKKILRTQ<br>ESECACVNGSCFTIMTDGPSDGLASYKIFKIEKGKVTKSIELNAPNSHYEECSCYPDTGKVMCVCRDNWH<br>GSNRPWVSFDQNLDYQIGYICSGVFGDNPRPEDGTGSCGPVYVDGANGVKGFSYRYGNGVWIGRTKSHSS<br>RHGFEMIWDPNGWTETDSKFSVRQDVVAMTDWSGYSGSFVQHPELTGLDCMRPCFWVELIRGRPKEKTIW<br>TSASSISFCGVNSDTVDWSWPDGAELPFSIDK

```
>ULA27163.1
MNPNQKIITIGSICLVVGPISLILQIGNIISIWISHSIQTGSQNHTGICNQNIITYKNSTWVNQTYVNIS
NTNVVAGKDTTSVILTGNSSLCPIRGWAIYSKDNSIRIGSKGDVFVIREPFISCSHLECRTFFLTQGALL
NDKHSNGTVKDRSPYRALMSCPVGEAPSPYNSRFESVAWSASACHDGMGWLTIGISGPDNGAVAVLKYNG
IITETIKSWRKKILRTQESECACVNGSCFTIMTDGPSDGLASYKIFKIEKGKVTKSIELNAPNSHYEECS
CYPDTGKVMCVCRDNWHGSNRPWVSFDQNLDYQIGYICSGVFGDNPRPEDGTGSCGPVYVDGANGVKGFS
YRYGNGVWIGRTKSHSSRHGFEMIWDPNGWTETDSKFSVRQDVVAMTDWSGYSGSFVQHPELTGLDCMRP
CFWVELIRGRPKEKTTIWTSASSISFCGVNSDTVDWSWPDGAELPFSIDK
```

```
>AEX96923.1
TIGSICLVVGLISLILQIGNIISIWISHSIQTGSQNHTGICNQNIITYKNSTWVKDTTSVILTGNSSLCP
IRGWAIYSKDNSIRIGSKGDVFVIREPFISCSHLECRTFFLTQGALLNDKHSSGTVKDRSPYRALMSCPV
GEAPSPYNSRFESVAWSASACHDGMGWLTIGISGPDNGAVAVLKYNGIITETIKSWRKKILRTQESECAC
VNGSCFTIMTDGPSDGLASYKIFKIEKGKVTKSIELNAPNSHYEECSCYPDTGKVMCVCRDNWHGSNRPW
VSFDQNLDYQIGYICSGVFGDNPRPEDGTGSCGPVYVDGANGVKGFSYRYGNGVWIGRTKSHSSRHGFEM
IWDPNGWTETDSKFSVRQDVVAMTDWSGYSGSFVQHPELTGLDCMRPCFWVELIRGRPKEKTIWTSASSI
SFCGVNSDTVDWSWPDGAELPFSIDK
```

Table S4. Different protein from hMPV, RSV, and influenza A virus.

Virus	Protein	Antigenic value	Antigenicity
**Human Metapneumovirus (hMPV)**	N	0.5243	Antigenic
	P	0.5131	Antigenic
	M	0.3939	Non- Antigenic
	F	0.4775	Antigenic
	M2-1	0.4212	Antigenic
	M2-2S	0.4285	Antigenic
	SH	0.3756	Non- Antigenic
	G	0.6768	Antigenic
	L	0.471	Antigenic
**Respiratory syncytial virus (RSV)**	NS1	0.3602	Non- Antigenic
	NS2	0.5592	Antigenic
	N	0.4307	Antigenic
	P	0.5066	Antigenic
	M	0.5250	Antigenic
	SH	0.6297	Antigenic
	G	0.4949	Antigenic
	F	0.5219	Antigenic
	M2	0.4669	Antigenic
	L	0.4440	Antigenic
**Influenza A Virus**	PB1	0.4910	Antigenic
	PA	0.5121	Antigenic
	hemagglutinin	0.5471	Antigenic
	PB2	0.7418	Antigenic
	nucleocapsid protein	0.6080	Antigenic
	matrix protein 2	0.4644	Antigenic
	hemagglutinin	0.4466	Non- Antigenic
	neuraminidase	0.5024	Antigenic
	matrix protein 2	0.6037	Antigenic
	nuclear export protein	0.4252	Antigenic
	nonstructural protein 1	0.3328	Non- Antigenic
	matrix protein 1	0.4828	Antigenic
	nucleocapsid protein	0.7694	Antigenic
	nonstructural protein 1	0.3611	Non- Antigenic

Table S5. Predicted HTL epitopes. The table shows the specific allele against which the HTL epitopes are predicted. Furthermore, it also shows the methods and percentile rank of each peptide. The HTL epitopes shown in bold are finally selected epitopes for chimeric multi-epitopes vaccine constructed.

Virus	Protein	Allele	Start	End	Peptide Sequence	Percentile Rank	Antigenicity Score
**Human Metapneumovirus**	Fusion protein	HLA-DRB1*07:01	229	243	RAVSYMPTSAGQIKL	0.06	0.8830
		HLA-DRB1*15:01	119	133	TAGIAIAKTIRLESE	0.12	0.8439
		HLA-DRB5*01:01	131	145	ESEVNAIKGALKTTN	0.13	0.4978
		HLA-DRB1*15:01	162	176	VRELKEFVSKNLTSA	0.13	-0.0826
		HLA-DRB4*01:01	63	77	GPSLIKTELDLTKSA	0.18	0.5887
		HLA-DRB1*03:01	415	429	ADTVTIDNTVYQLSK	0.26	0.0778
		HLA-DRB1*03:01	218	232	SLDLMNDAELARAVS	0.26	0.4532
		HLA-DRB4*01:01	200	214	FLNVVRQFSDNAGIT	0.30	0.0926
		HLA-DRB5*01:01	152	166	GNGVRVLATAVRELK	0.33	0.3485
		HLA-DRB1*07:01	231	245	VSYMPTSAGQIKLML	0.60	0.5663
**Respiratory syncytial virus**	Fusion protein	HLA-DRB3*02:02	234	248	TREFSVNAGVTTPVS	0.04	0.2788
		HLA-DRB1*03:01	263	277	DMPITNDQKKLMSN	0.04	0.2076
		HLA-DRB3*02:02	238	252	GCDYVSNKGVDTVSV	0.11	0.6006
		HLA-DRB3*02:02	270	284	QKKLMSNNVQIVRQQ	0.12	0.2284
		HLA-DRB1*15:01	80	94	KQELDKYKSAVTELQ	0.13	-0.2571
		HLA-DRB4*01:01	75	89	KVKLIKQELDKYKSA	0.16	-0.7015
		HLA-DRB3*02:02	269	283	DQKKLMSNNVQIVRQ	0.16	0.0835
		HLA-DRB4*01:01	392	406	DCKIMTSKTDVSSSV	0.23	0.1144
		HLA-DRB1*15:01	472	486	EPIINFYDPLVFPSD	0.29	-0.0213
		HLA-DRB3*02:02	436	450	SNGCDYVSNKGVDTV	0.32	0.3386
**Infl uenz** [illegible]	ne ur a	HLA-DRB1*15:01	162	176	SDGLASYKIFKIEKG	0.04	0.1598

		HLA-DRB3*01:01	12	26	WAIYSKDNSIRIGSK	0.10	1.1726
		HLA-DRB3*01:01	249	263	SCGPVYVDGANGVKG	0.17	0.2163
		HLA-DRB3*02:02	178	192	VTKSIELNAPNSHYE	0.28	0.3843
		HLA-DRB1*15:01	116	130	GAVAVLKYNGIITET	0.41	0.4093
		HLA-DRB3*02:02	180	194	KSIELNAPNSHYEEC	0.66	0.1576
		HLA-DRB3*01:01	216	230	RPWVSFDQNLDYQIG	0.78	0.6706
		HLA-DRB1*07:01	44	58	CRTFFLTQGALLNDR	0.90	0.4648
		HLA-DRB1*07:01	119	133	AVLKYNGIITETIKS	1.20	0.1688
		HLA-DRB3*01:01	214	228	SNRPWVSFDQNLDYQ	1.30	0.6043
		HLA-DRB5*01:01	334	348	RPCFWVELIRGRPKE	2.20	0.9819

Table S6. Predicted HTL epitopes. The table shows the specific allele against which the HTL epitopes are predicted. Furthermore, it also shows the methods and percentile rank of each peptide. The HTL epitopes shown in bold are finally selected epitopes for chimeric multi-epitopes vaccine constructed.

Virus	Protein	Allele	Start	End	Peptide Sequence	Percentile Rank	Antigenicity Score
**Human Metapneumovirus**	Fusion protein	HLA-DRB1*07:01	229	243	RAVSYMPTSAGQIKL	0.06	0.8830
		HLA-DRB1*15:01	119	133	TAGIAIAKTIRLESE	0.12	0.8439
		HLA-DRB5*01:01	131	145	ESEVNAIKGALKTTN	0.13	0.4978
		HLA-DRB1*15:01	162	176	VRELKEFVSKNLTSA	0.13	-0.0826
		HLA-DRB4*01:01	63	77	GPSLIKTELDLTKSA	0.18	0.5887
		HLA-DRB1*03:01	415	429	ADTVTIDNTVYQLSK	0.26	0.0778
		HLA-DRB1*03:01	218	232	SLDLMNDAELARAVS	0.26	0.4532
		HLA-DRB4*01:01	200	214	FLNVVRQFSDNAGIT	0.30	0.0926
		HLA-DRB5*01:01	152	166	GNGVRVLATAVRELK	0.33	0.3485
		HLA-DRB1*07:01	231	245	VSYMPTSAGQIKLML	0.60	0.5663
**Respiratory syncytial virus**	Fusion protein	HLA-DRB3*02:02	234	248	TREFSVNAGVTTPVS	0.04	0.2788
		HLA-DRB1*03:01	263	277	DMPITNDQKKLMSN	0.04	0.2076
		HLA-DRB3*02:02	238	252	GCDYVSNKGVDTVSV	0.11	0.6006
		HLA-DRB3*02:02	270	284	QKKLMSNNVQIVRQQ	0.12	0.2284
		HLA-DRB1*15:01	80	94	KQELDKYKSAVTELQ	0.13	-0.2571
		HLA-DRB4*01:01	75	89	KVKLIKQELDKYKSA	0.16	-0.7015
		HLA-DRB3*02:02	269	283	DQKKLMSNNVQIVRQ	0.16	0.0835
		HLA-DRB4*01:01	392	406	DCKIMTSKTDVSSSV	0.23	0.1144
		HLA-DRB1*15:01	472	486	EPIINFYDPLVFPSD	0.29	-0.0213
		HLA-DRB3*02:02	436	450	SNGCDYVSNKGVDTV	0.32	0.3386
**Influenza A Virus**	Neuraminidase	HLA-DRB1*15:01	162	176	SDGLASYKIFKIEKG	0.04	0.1598
		HLA-DRB3*01:01	12	26	WAIYSKDNSIRIGSK	0.10	1.1726
		HLA-DRB3*01:01	249	263	SCGPVYVDGANGVKG	0.17	0.2163

		HLA-DRB3*02:02	178	192	VTKSIELNAPNSHYE	0.28	0.3843
		HLA-DRB1*15:01	116	130	GAVAVLKYNGIITET	0.41	0.4093
		HLA-DRB3*02:02	180	194	KSIELNAPNSHYEEC	0.66	0.1576
		HLA-DRB3*01:01	216	230	RPWVSFDQNLDYQIG	0.78	0.6706
		HLA-DRB1*07:01	44	58	CRTFFLTQGALLNDR	0.90	0.4648
		HLA-DRB1*07:01	119	133	AVLKYNGIITETIKS	1.20	0.1688
		HLA-DRB3*01:01	214	228	SNRPWVSFDQNLDYQ	1.30	0.6043
		HLA-DRB5*01:01	334	348	RPCFWVELIRGRPKE	2.20	0.9819

Table S7. Predicted linear B-Cell epitopes for the virus polypeptide. The predicted score for each peptide is given against each B-cell epitope.

Virus	Protein	Start Position	Epitope	Score	Antigenicity Score
Human Metapneumovirus	Fusion protein	510	IIIIIKKTRKPAGAPP	0.92	1.0870
		283	CWIIKAAPSCSEKDGN	0.92	0.8104
		301	CLLREDQGWYCKNAGS	0.89	0.5101
		238	AGQIKLMLENRAMVRR	0.89	0.0882
		92	EEQIENPRQSRFVLGA	0.88	0.2275
		434	QHVIKGRPVSNSFDPI	0.88	0.9931
		363	VSTGRHPISMVALSPL	0.87	0.9390
		312	KNAGSTVYYPNEKDCE	0.87	0.4609
		125	AKTIRLESEVNAIKGA	0.87	0.3283
		409	YITNQDADTVTIDNTV	0.86	0.3534
Respiratory syncytial virus	Fusion protein	479	DPLVFPSDEFDASISQ	0.92	0.0842
		106	RARRELPRFMNYTLNN	0.92	-0.0128
		306	YGVIDTPCWKLHTSPL	0.91	0.5064
		383	NIDIFNPKYDCKIMTS	0.89	0.7806
		334	TFSNGCDYVSNKGVDT	0.88	0.3822
		329	GSNICLTRTDRGWYCD	0.87	0.8391
		23	ASSQNITEEFYQTTCS	0.86	0.2400
		211	SCSISNIETVIEFQQK	0.86	1.0332
		196	KNYIDKQLLPIVNKQS	0.86	0.3791
		54	TSVITIELSNIKENKCd	0.86	0.6166
Influenza A Virus	neuraminidase	223	CFTIMTDGPSDGLASY	0.94	-0.2483
		315	DGTGSCGPVYVDGANG	0.93	-0.1810
		282	HGSNRPWVSFDQNLDY	0.93	0.2757
		356	FEMIWDPNGWTETDSK	0.92	0.1997
		35	SHSIQTGSQNHTGICN	0.92	0.6373
		299	IGYICSGVFGDNPRPK	0.91	0.5897
		273	VMCVCRDNWHGSNRPW	0.91	0.1602
		261	EECSCYPDTGKVMCVC	0.91	0.4128
		329	NGVKGFSYRYGNGVWI	0.90	0.3707
		131	NGTVKDRSPYRALMSC	0.89	0.6479
		251	SIELNAPNSHYEECSC	0.88	0.2283

Table S8. Key Hydrogen Bonds Between TLR4 and Vaccine Construct During MD Simulation.

TLR4 Residues	Vaccine Residues	Frames Observed	Bond Life	Average Distance (Å)	Standard Deviation (Å)
459-ND2	876-O	6261	62.60%	2.82	0.09
357-O	855-NH1	3808	38.10%	2.84	0.09
458-OE1	875-NZ	3486	34.90%	2.82	0.10
412-OE1	817-NH1	2851	28.50%	2.80	0.09
455-OG	877-OD1	2221	22.20%	2.77	0.13
409-OE1	817-NH1	2027	20.30%	2.81	0.09
433-NH2	877-OD1	457	4.57%	2.86	0.10
455-CB	877-OD1	204	2.04%	2.94	0.05
433-NH2	876-OD2	173	1.73%	2.81	0.10
433-NH1	876-OD2	167	1.67%	2.84	0.09
361-NZ	834-O	160	1.60%	2.81	0.10
